# Bridging BET bromodomain and immune checkpoint inhibitors through generative bioorganic frameworks for next-generation cancer immunotherapy

**DOI:** 10.1039/d6md00450d

**Published:** 2026-09-24

**Authors:** Mohamed K. Diab, Asmaa S. A. Yassen, Sherif Ashraf Fahmy, Heba F. Ashour, Mohamed S. Nafie

**Affiliations:** a Pest Physiology Department, Plant Protection Research Institute, Agricultural Research Center Giza 12311 Egypt; b Department of Medicinal Chemistry, Faculty of Pharmacy, Galala University New Galala 43713 Egypt; c Pharmaceutical Organic Chemistry Department, Faculty of Pharmacy, Suez Canal University Ismailia 41522 Egypt; d Department of Pharmacy, Institute of Pharmaceutics and Biopharmaceutics, Marburg University Marburg Germany sherif.fahmy@pharmazie.uni-marburg.de; e Department of Chemistry, College of Sciences, University of Sharjah P.O 27272 United Arab Emirates mohamed.elsayed@sharjah.ac.ae; f Bioinformatics and Functional Genomics Research Group, Research Institute of Sciences and Engineering (RISE), University of Sharjah Sharjah United Arab Emirates; g Department of Chemistry, Faculty of Science, Suez Canal University Ismailia 41522 Egypt mohamed_nafie@science.suez.edu.eg

## Abstract

Bromodomain and extraterminal domain (BET) proteins are epigenetic readers that couple chromatin acetylation to oncogenic and immune-regulatory gene transcription, making them attractive targets for combination with immune checkpoint inhibitors (ICIs) in precision oncology. This review synthesizes current mechanistic, medicinal-chemistry, and translational evidence on BET–ICI combination strategies. We describe how BET blockade reshapes tumor chromatin architecture, suppresses *PD-L1* expression, and enhances antigen presentation, thereby increasing tumor susceptibility to immune-mediated attack. We further summarize preclinical efficacy data for BET–ICI combinations, including systems-biology and agent-based computational models that predict substantially greater tumor-volume reduction with combination regimens relative to monotherapy in selected preclinical settings; however, early-phase clinical experience with these combinations remains limited and has not yet reproduced this magnitude of benefit. Beyond the mechanism, we review the medicinal chemistry underlying BET inhibitor design and the emerging landscape of BET–ICI hybrid and conjugate therapeutics, distinguishing combinations with direct experimental support from conceptual pairings that remain hypothesis-generating. Finally, we discuss how systems biology and generative, AI-guided molecular design may inform resistance-mechanism prediction, toxicity mitigation, and biomarker-guided, patient-specific compound selection. Collectively, this review outlines a framework for how the convergence of epigenetic reprogramming, immune modulation, and generative bioorganic chemistry could inform the rational design of next-generation BET–ICI combination therapeutics.

## Abstract 2

Generative bioorganic frameworks guide BET bromodomain inhibitor discovery and combination with immune checkpoint inhibitor (ICI) blockade, suppressing MYC/PD-L1 signaling and enhancing antitumor immune responses.

## Graphical abstract

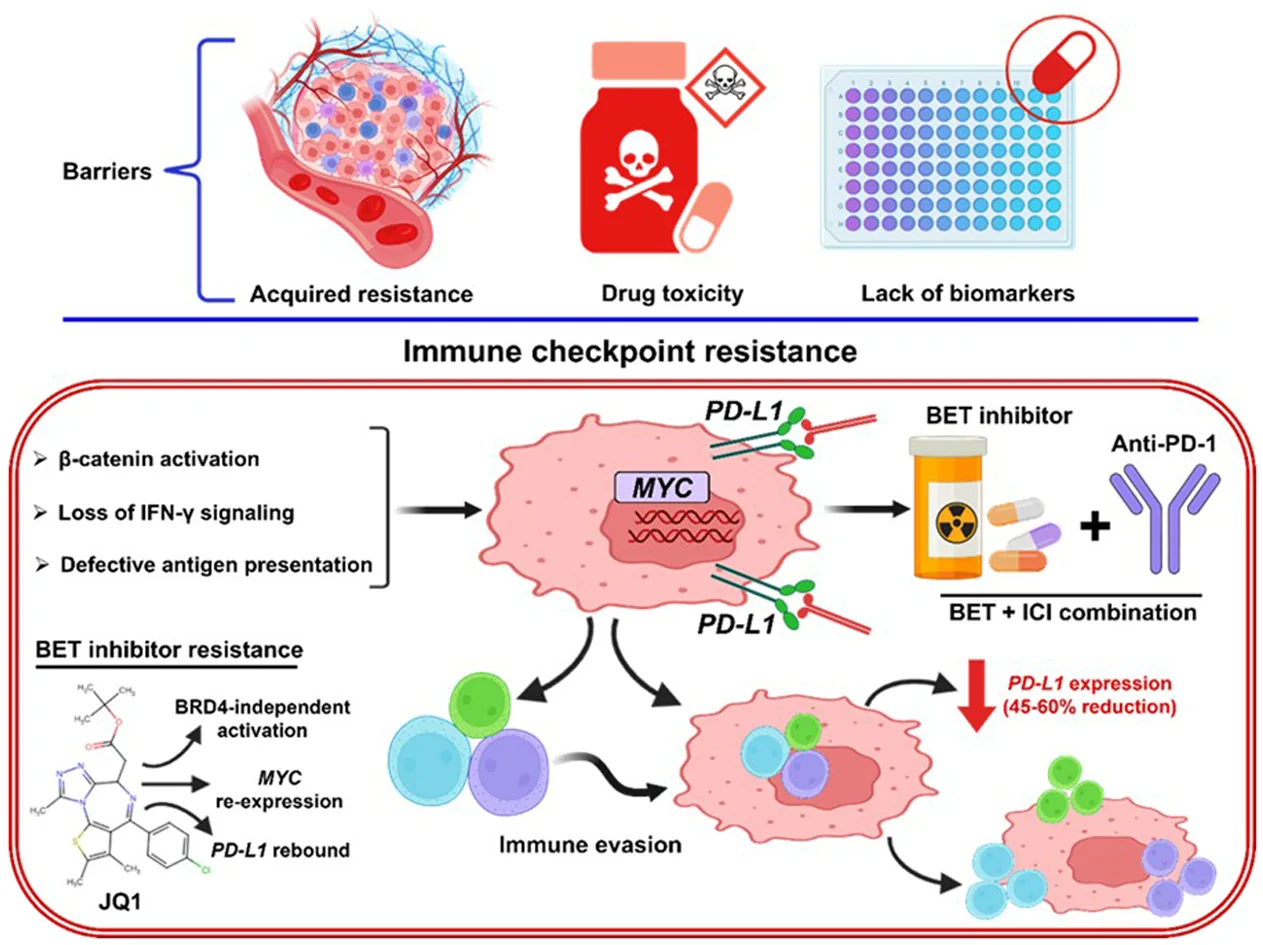

## 1. Introduction

Cancer is now widely acknowledged as a genetic and epigenetic disease, while abnormal chromatin remodeling and immune escape are among the hallmarks of cancer survival and therapy resistance. Epigenetic dysregulation, particularly abnormal histone modifications, DNA methylation, and chromatin remodeling, can significantly affect tumor immunogenicity by inhibiting antigen presentation or disrupting cytokine signaling pathways responsible for immune recognition and elimination of tumors.^1,2^ Immune checkpoint inhibitors (ICIs), which include anti-PD-1 and anti-CTLA-4, proved to be a breakthrough in cancer therapy by reinvigorating cytotoxic T cells; yet only a minority of patients have a sustainable response owing, largely, to epigenetically mediated immune exclusion and T-cell dysfunction.^3,4^

Epigenetic therapies can alter tumor immunogenicity by restoring antigen-processing pathways, modifying inflammatory signaling, and disrupting transcriptional programs that support immune escape. Among these agents, bromodomain and extraterminal domain (BET) inhibitors are of particular interest because BET proteins couple acetylated chromatin to the expression of oncogenic and immune-regulatory genes.^5,6^ This provides a mechanistic rationale for combining BET inhibition with immune checkpoint blockade, particularly in tumors with attributes of immune exclusion or adaptive resistance.^7^ However, preclinical success and use have been limited: uncertainties remain regarding tumor context, treatment sequence, pharmacodynamic biomarkers of effect, mechanisms of innate or acquired resistance to therapy, and the potential for overlapping toxicities related to BET inhibition.^8,9^ Throughout this review, the term “epigenetic” refers to the molecular mechanisms regulating chromatin structure and gene expression, whereas “epigenomic” is reserved for genome-wide profiling or characterization of these regulatory modifications. This review therefore focuses on the molecular mechanisms, medicinal chemistry, preclinical evidence, and emerging clinical opportunities of BET–ICI combinations with a view to showing how systems biology and generative molecular design may drive more rational patient and compound selection.

## 2. Functional diversity and oncogenic roles of BET proteins in tumor epigenetic remodeling

**Fig. 1:**
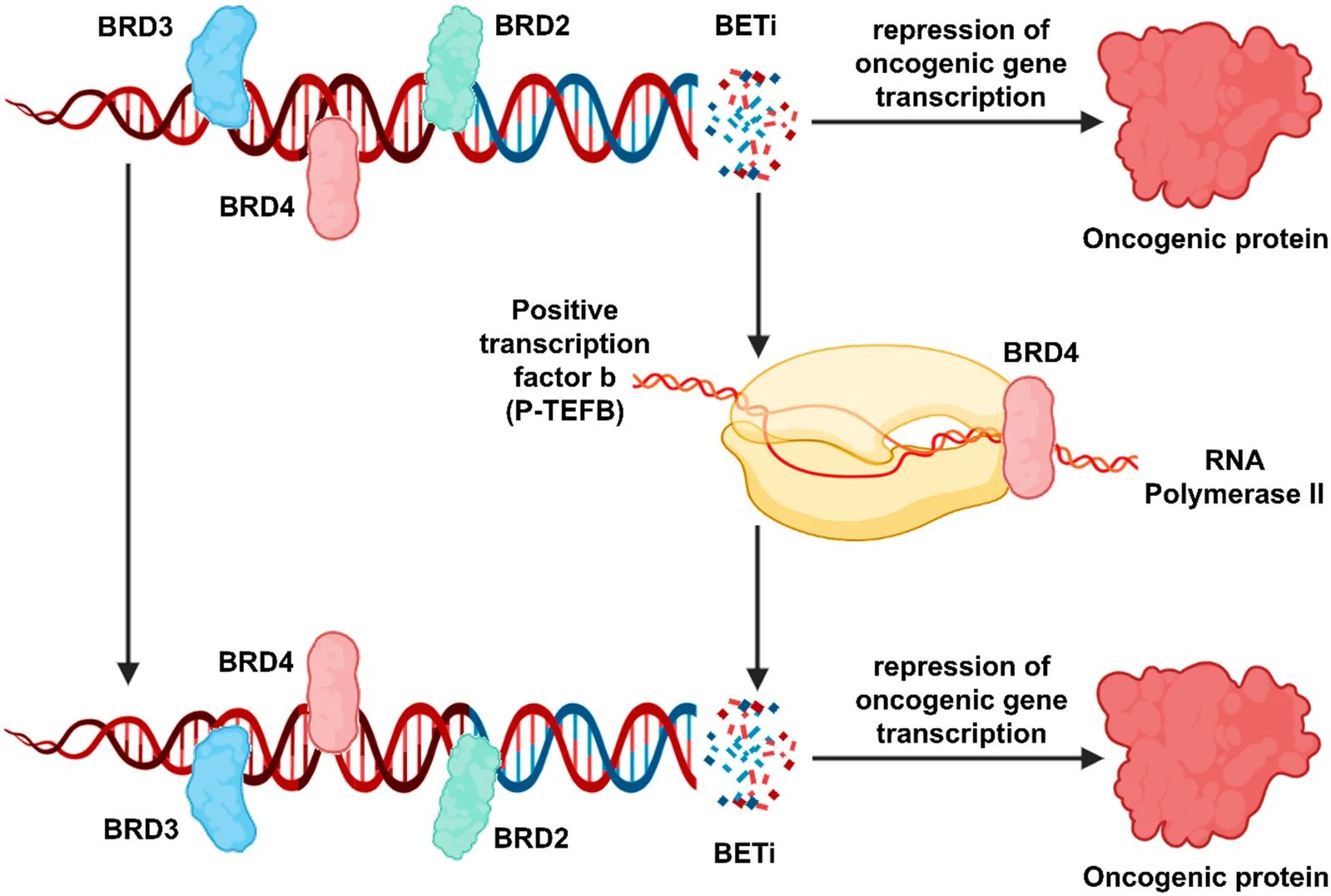
Basis of BET protein inhibition in tumor epigenetic remodeling. Partially created based on (https://www.biorender.com/).

The bromodomain and extraterminal domain (BET) family comprises BRD2, BRD3, BRD4, and the testis-restricted member BRDT. Each protein contains two tandem N-terminal bromodomains, BD1 and BD2, which recognize acetylated lysine residues on histones and other nuclear proteins, together with an extraterminal domain that mediates protein–protein interactions.^10^ Despite this conserved architecture, BET family members are not functionally identical. BRD2 is strongly associated with transcriptional regulation of cell-cycle progression and inflammatory signaling, whereas BRD3 contributes to lineage-specific transcriptional programs and interacts with selected acetylated transcription factors.^11,12^ BRD4 has a broader and better characterized role in enhancer-dependent transcription, transcriptional elongation, and maintenance of oncogenic super-enhancer programs. In contrast, BRDT is predominantly expressed in male germ cells and has a more restricted direct relevance to most somatic malignancies.^13^ BET proteins, including BRD2, BRD3, BRD4, and BRDT, are “epigenetic readers” targeted to acetylated lysine residues on histones H3 and H4 that open chromatin and activate transcription of the oncogenic drivers (Fig. 1).^14^

**Fig. 2:**
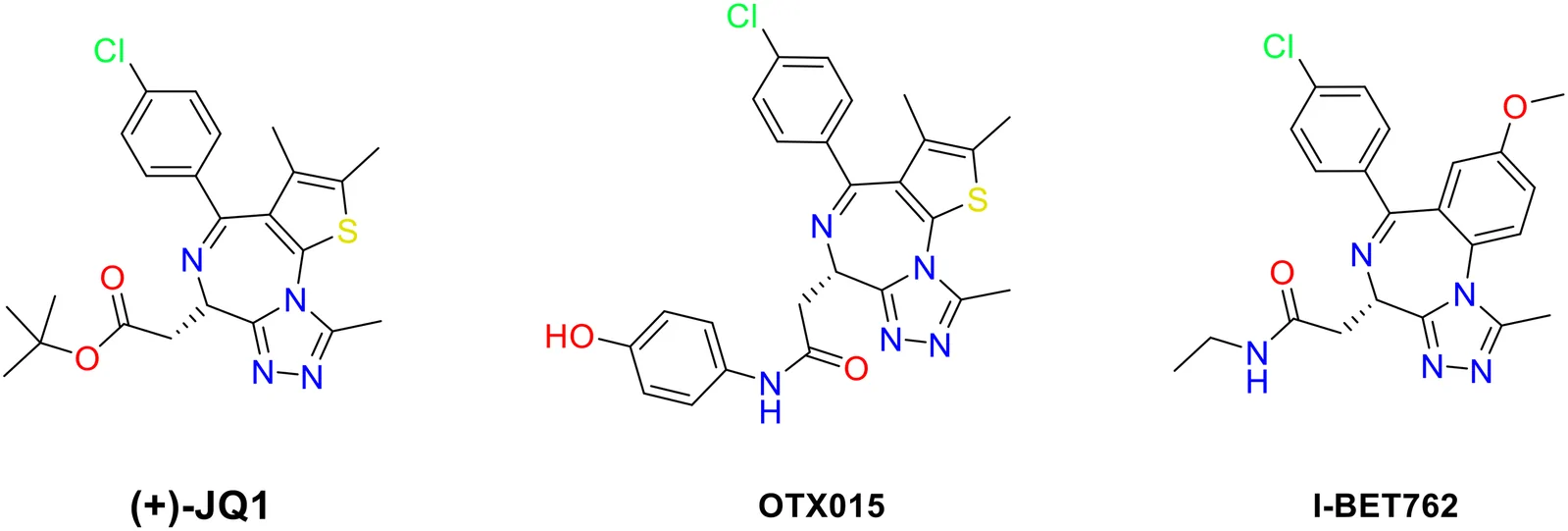
Structures of (+)-JQ1, OTX015, and I-BET762.

BET proteins are dysregulated across a wide range of hematological and solid tumors, where they support context-dependent transcriptional programs governing proliferation, survival, lineage identity, DNA-damage responses, inflammation, and immune evasion. Their cancer relevance therefore extends beyond a single tumor type, making BET proteins attractive pan-cancer therapeutic targets. However, the degree of dependence on individual BET family members differs among malignancies. It may be shaped by tumor lineage, enhancer architecture, oncogenic drivers, and the composition of the tumor microenvironment.^15,16^ Among the BET proteins, BRD4 has received the greatest attention in cancer research because of its well-established role in recruiting transcriptional machinery to promoters and super-enhancers, sustaining expression of oncogenic drivers such as *MYC*, *BCL2*, and *CCND1*, and regulating immune-relevant genes including *PD-L1*. The greater emphasis on BRD4 in this section therefore reflects the depth of available mechanistic and pharmacological evidence rather than an assumption that BRD2 and BRD3 are biologically redundant. Where evidence permits, the distinct contributions of BRD2, BRD3, and BRD4 are discussed separately.^15,17^ Suppression of these bromodomains, mainly through small molecules like JQ1, OTX015, and I-BET762 (Fig. 2), can disrupt interactions with BET-acetyl-lysine and consequently reduce the transcription of super-enhancer target genes (*MYC*, *BCL2*, and *CCND1*).^18,19^

In a more quantitative manner, JQ1 treatment caused preferential loss of BRD4 at *MYC*-associated super-enhancers in multiple myeloma models, where super-enhancers were occupied by up to 16-fold more BRD4 and 18-fold more mediator than typical enhancers, rendering *MYC* transcription disproportionately sensitive to BET bromodomain disruption.^20^ At the mechanistic level, BRD4 serves as a scaffold, bringing the positive transcription elongation factor b (PTEFb) complex to promoters, which, in turn, allows RNA polymerase II phosphorylation and productive elongation. Inhibition of BET protein recruitment prevents this effect, leading to genome-wide transcriptional stalling and specific suppression of the hyperacetylated enhancer-driven oncoproteins.^21^ The efficacy of BET inhibition depended on *GLI1*, as an inhibitor was similarly effective in reducing tumor growth and *GLI1* expression by >50% (*p* < 0.01) in pancreatic ductal adenocarcinoma (PDAC) through an effect on the Hedgehog pathway.^22^ In addition, when tested in osteosarcoma models, JQ1 decreased the tumor volume by 67% by blocking the BRD4-dependent RUNX2 transcription and bone remodeling.^23^ These results highlight BET inhibition as a quantitative and mechanistically defined approach to reshape tumor epigenomes. Collectively, these findings establish BET inhibition as a mechanistically defined strategy for disrupting enhancer-dependent oncogenic transcription. In this review, we will discuss how the chromatin modification mechanisms cause changes in tumor immune regulation and ultimately regulate *PD-L1* expression levels and antigen presentation.^24^ The genetic and pharmacological studies demonstrate that BET proteins influence tumor immunogenicity through multiple mechanisms, including antigen presentation, immune-checkpoint regulation, and remodeling of the tumor microenvironment, as summarized in Table 1.

**Table 1:** Genetic and pharmacological evidence linking BET proteins to tumor immunogenicity and microenvironment remodeling

Cancer context	Perturbation	Main immune-related effect	Translational implication	References
Head and neck squamous cell carcinoma	Genetic BRD4 depletion and/or BET inhibition	Increased MHC class I expression and CD8^+^ T-cell-dependent activity	Supports enhanced sensitivity to anti-PD-1 therapy	25
High-grade serous ovarian cancer	AZD5153	Reduced M2-like tumor-associated macrophage polarization	Indicates remodeling of the myeloid tumor microenvironment	26
*Myc*-driven lymphoma	Inducible BRD4 depletion and pharmacological BET inhibition	Reduced CD274/*PD-L1* transcription; maximal activity required an intact immune system	Supports direct BRD4 regulation of checkpoint signaling and combination with PD-1 blockade	27
Ovarian cancer	JQ1 or related BET inhibition	Reduced *PD-L1* and increased cytotoxic T-cell activity	Supports immune-mediated contribution to tumor control	28
Selected solid-tumor models	JQ1 or dual PI3K/BRD4 inhibition	Increased CD8^+^ T-cell infiltration and expression of IFN-γ and granzyme B	Supports broader immune activation, although effects may not be BRD4 specific	29
Triple-negative breast cancer	JQ1	Reduced *PD-L1* and altered T-cell subset distribution	Suggests combined tumor-intrinsic and systemic immune effects	30

## 3. Immune checkpoint inhibitors: molecular pathways and resistance mechanisms

**Fig. 3:**
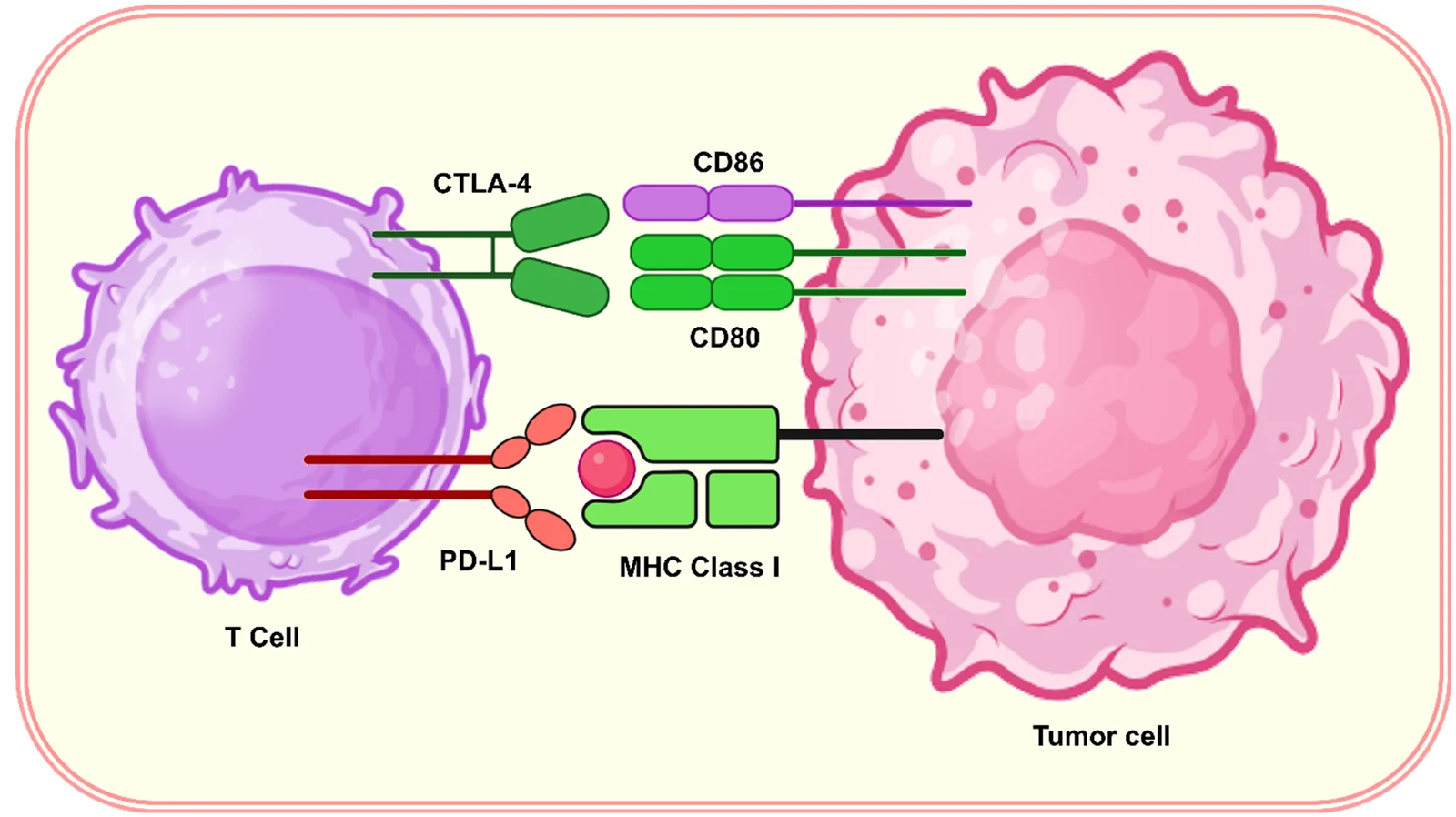
Immune checkpoint blockade and T-cell reinvigoration. Partially created based on (https://www.biorender.com/).

Immune checkpoint inhibitors (ICIs) have revolutionized contemporary oncology by reinvigorating T-cell–mediated antitumor immunity. The major molecular pathways being targeted are CTLA-4 and PD-1/*PD-L1*.^31^ CTLA-4 expressed on activated T cells also competes with CD28 for binding to CD80/CD86 on antigen-presenting cells and inhibits T-cell priming; its blockade by ipilimumab results in the potentiation of T-cell proliferation and effector activity, as shown in (Fig. 3).^32^

Likewise, PD-1–*PD-L1* blockades *via* nivolumab or pembrolizumab renew the effector function of exhausted cytotoxic T cells in the tumor microenvironment.^33^ ICIs have shown remarkable success as a therapeutic option in various malignancies when used clinically. For advanced melanoma, the estimated 5 year overall survival with pembrolizumab monotherapy was 34% in all treated patients and 41% in treatment-naive patients in the KEYNOTE-001 trial, substantially exceeding the historical 5 year survival rate of approximately 10% reported before the introduction of immune checkpoint inhibitors.^34^ Non-small-cell lung cancer (NSCLC) ORR was 19.2%, and median survival was improved from 9.4 to 12.2 months *versus* docetaxel controls with nivolumab.^35^ Also, with atezolizumab plus bevacizumab in hepatocellular carcinoma, the median overall survival was found to be 19.2 months (a 42% lower risk of death in comparison with sorafenib).^36^

Despite these successes, resistance does develop, both primary and acquired. Tumor-intrinsic changes, such as β2-microglobulin (B2M) loss, JAK1/2 mutations, or Wnt/β-catenin activation, reduce antigen presentation and T-cell infiltration.^37^ Additionally, tumor-extrinsic processes, including the induction of alternative checkpoints (TIM-3, LAG-3) and immunosuppressive cytokines (IL-10, TGF-β), further hinder immune activation by restricting the immune response.^38^ The emergence of combination therapies (epigenetic modifiers, metabolic modulators, and neoantigen vaccines) targets these resistance pathways in order to restore durable tumor immunogenicity.^39^Table 2 encompasses both clinically approved ICIs (nivolumab, pembrolizumab, and atezolizumab) and emerging ones (TIGIT, LAG-3, and TIM-3 inhibitors), which together define the current state of immune checkpoint modulation in cancer.

**Table 2:** Immune checkpoint inhibitors have been successful in immunotherapy

Inhibitor	Type	Targeted cancer	Molecular pathway	Molecular target	*K*_D_/IC_50_ value	References
Atezolizumab	Monoclonal antibody	NSCLC, urothelial cancer	PD-1/*PD-L1*	PD-1	1.0 nM	40
Avelumab	Monoclonal antibody	Merkel cell carcinoma	PD-1/*PD-L1*	*PD-L1*	NA	41
B7-H3 mAb (enoblituzumab)	Monoclonal antibody	Prostate cancer	B7-H3 pathway	B7-H3	NA	42
BT7480	Small molecule	NSCLC	Nectin-4/TIGIT	Nectin-4, NF-κB	6.0 nM	43
Cadonilimab	Bispecific antibody	Lung cancer	PD-1/CTLA-4 dual blockade	PD-1, CTLA-4	NA	44
CD276 inhibitor	Small molecule	Pancreatic cancer	B7-H3 pathway	CD276	NA	45
CDX-585	Bispecific antibody	NSCLC	PD-1/*PD-L1*, CTLA-4	PD-1, ILT-4	0.21	46
Cemiplimab	Monoclonal antibody	Cutaneous squamous cell carcinoma	PD-1/*PD-L1*	PD-1	12.5 nM	47
CPI-006	Monoclonal antibody	Solid tumors	CD73/adenosine	CD73	NA	48
CPI-444	Small molecule inhibitor	Colorectal cancer	A_2A_ receptor	A_2A_R	70.0 nM	49
[^89^Zr]Zr-DFO-durvalumab	Monoclonal antibody	NSCLC, bladder cancer	PD-1/*PD-L1*	*PD-L1*	NA	50
Favezelimab	Monoclonal antibody	Colorectal cancer	LAG-3/MHC II	LAG-3	NA	51
Galectin-9	Monoclonal antibody	NSCLC	Tim-3-galectin-9 pathway	TIM-3	NA	52, 53
GITR-ligand	Agonist antibody	Melanoma	GITR/TNFRSF18	GITR	NA	54
Gln(TrT)	Small molecule inhibitor	Colorectal cancer (MC38 model)	TIGIT/PVR, PD-1/*PD-L1*	TIGIT, PD-1	NA	55
Ipilimumab	Monoclonal antibody	Melanoma	CTLA-4/CD80/CD86	CTLA-4	18.2 nM	56
MBG453	Monoclonal antibody	AML, leukemia	TIM-3/galectin-9	TIM-3	NA	57
MEDI5752	Bispecific antibody	NSCLC	PD-1/CTLA-4 dual blockade	PD-1, CTLA-4	NA	58
MK-4280	Monoclonal antibody	Gastric cancer	LAG-3/MHC II	LAG-3, PD-1	6.6 nM	59
Nivolumab	Monoclonal antibody	Melanoma, NSCLC, cell renal carcinoma (RCC)	PD-1/*PD-L1*	PD-1	2.6 nM	60, 61
Ociperlimab	Monoclonal antibody	Lung cancer	TIGIT/PVR	TIGIT	NA	62
PCC0208025 (BMS202)	Small molecule inhibitor	Melanoma	PD-1/*PD-L1*	*PD-L1*	235 nM	63
PDI-1	Small molecule inhibitor	NSCLC, melanoma	PD-1/*PD-L1*	PD-1/*PD-L1*	NA	64
Pembrolizumab	Monoclonal antibody	Melanoma, NSCLC	PD-1/*PD-L1*	PD-1	NA	65
Relatlimab	Monoclonal antibody	Melanoma	LAG-3/MHC II	LAG-3	1.05 nM	66
Sintilimab	Monoclonal antibody	Hepatocellular carcinoma	PD-1/*PD-L1*	PD-1	4.4 μg mL^−1^	67, 68
TIM3	Monoclonal antibody	Solid tumors	TIM-3/galectin-9	TIM-3	NA	69
Tiragolumab	Monoclonal antibody	NSCLC, ovarian cancer	TIGIT/PVR	TIGIT	NA	70, 71
Toripalimab	Monoclonal antibody	Nasopharyngeal carcinoma	PD-1/*PD-L1*	PD-1, NF-κB	NA	72, 73
Tremelimumab	Monoclonal antibody	Mesothelioma	CTLA-4/CD80/CD86	CTLA-4	18.2 nM	74, 75
TSR-022	Monoclonal antibody	NSCLC	TIM-3/galectin-9	TIM-3	9.4 nM	76, 77
Urelumab	Agonist antibody	Lymphoma	CD137 pathway	4-1BB	NA	78
Utomilumab	Agonist antibody	Lymphoma	CD137 pathway	4-1BB	NA	78, 79
XmAb22841	Bispecific antibody	Melanoma	CTLA-4/LAG-3 dual blockade	CTLA-4, LAG-3	0.67 nM	51

### 3.1. Clinical activity and limitations of immune checkpoint blockade

Despite durable responses in a subset of patients, the clinical benefit of immune checkpoint inhibitors remains heterogeneous across tumor types and patient populations. Many patients exhibit no meaningful initial response, whereas others relapse after a period of tumor control. Clinical interpretation is further complicated by delayed responses, pseudoprogression, mixed responses among metastatic lesions, and, in uncommon cases, rapid disease acceleration described as hyperprogressive disease. Treatment may also produce immune-related adverse events affecting the skin, gastrointestinal tract, endocrine organs, liver, lungs, heart, nervous system, and other tissues. These limitations emphasize that checkpoint expression alone does not determine therapeutic outcome and that clinical efficacy depends on the integrity of multiple steps in the cancer-immunity cycle.^80,81^

### 3.2. Primary, adaptive, and acquired resistance

Immune checkpoint blockade can broadly be categorized into primary, adaptive, or acquired resistance. Definitions of primary resistance involve no clinically meaningful benefit from the outset and may arise because of an innate inability of tumors to respond (low tumor antigenicity, defective antigen presentation, immune exclusion, or a prohibitively suppressive tumor microenvironment). The initial active antitumor immune response, however, can induce compensatory reactive inhibitory mechanisms responsible for adaptive resistance (*e.g.*, by interferon-driven *PD-L1* expression or upregulation of alternative checkpoints). Acquired resistance occurs after an initial response and reflects the selection or emergence of tumor-cell and microenvironmental states that evade renewed immune pressure. These categories overlap biologically, and more than one resistance mechanism may coexist within the same patient or even among different lesions.^39,82^

### 3.3. Tumor-intrinsic resistance pathways

Tumor-intrinsic resistance can occur at multiple steps in immune recognition. First, neoantigen-low tumors or tumors that express either few or relatively non-immunogenic antigens may fail to elicit strong T-cell responses. Another mechanism relates to defects in processing and presenting antigens, such as β2-microglobulin loss, inhibition by HLA class I molecules, or mutations in the transporter associated with antigen processing, which precludes recognition by CTLs. Moreover, the decreased sensitivity to immune-mediated killing can be partially attributed to impairment of interferon-γ signaling through JAK1, JAK2, or STAT1 or downstream transcriptional effectors that cause a downregulation in antigen presentation. In contrast, prolonged or aberrant interferon signaling can drive adaptive resistance through the induction of *PD-L1* and other inhibitory programs.^83,84^

Oncogenic signaling pathways also contribute to immune exclusion. Activation of Wnt/β-catenin signaling has been associated with defective dendritic-cell recruitment and reduced T-cell infiltration, while constitutive MAPK, PI3K–AKT–mTOR, *MYC*, and selected receptor-tyrosine-kinase programs can suppress inflammatory chemokines, enhance checkpoint-ligand expression, or promote an immunosuppressive metabolic state. PTEN loss could further increase signaling through the PI3K pathway and hinder T-cell infiltration into tumors. Not all tumors are affected uniformly by these pathways; the impact of genes in this pathway may depend upon tumor lineage, co-occurring genomic alterations, and the immune context.^85–87^

### 3.4. Tumor-microenvironment and host-mediated resistance

Tumor-extrinsic resistance is generally due to the tumor microenvironment, a complex collection of immune, stromal, vascular, and metabolic components. Myeloid-derived suppressor cells, regulatory T cells, tumor-associated macrophages, and dysfunctional dendritic cells localize into tumors, generating a microenvironment that inhibits T-cell priming and effector function using IL-10, TGF-β, arginase, indoleamine 2,3-dioxygenase, reactive oxygen species, and other suppressive mediators. On the other hand, cancer-associated fibroblasts and deposition of the extracellular matrix create a physical–biochemical barrier to lymphocyte infiltration, generating a T-cell-excluded phenotype. These abnormalities in tumor vessels and the further upregulation of endothelial anergy are going to limit leukocyte trafficking.^88,89^

In addition, T-cell dysfunction can persist even after PD-1 or CTLA-4 blockade due to terminal exhaustion, poor co-stimulation, loss of antigen-specific clones, or compensatory expression of alternative inhibitory receptors such as LAG-3, TIM-3, TIGIT, and VISTA. Adenosine accumulation *via* the CD39–CD73 axis, lactate production, hypoxia, nutrient depletion, and altered tryptophan metabolism can also inhibit effector lymphocytes and promote regulatory and myeloid populations. At the host level, corticosteroid or antibiotic exposure may play a role, as well as comorbidities, prior therapies, and inter-patient variation in intestinal microbiome composition and diversity, but the extent of these effects seen in basic science studies does not always translate into clinically reproducible findings.^90,91^

## 4. BET-dependent remodeling of tumor immunogenicity and the tumor microenvironment

BET proteins coordinate tumor immunity at multiple levels beyond *PD-L1* transcription and antigen presentation. By controlling enhancer- and promoter-dependent gene expression, BET proteins can alter malignant cells, infiltrating lymphocytes, macrophages, dendritic cells, myeloid-derived suppressor cells, and stromal populations. Consequently, pharmacological BET inhibition may influence immune-checkpoint expression, antigen processing, inflammatory and interferon signaling, immune-cell trafficking, macrophage polarization, extracellular-matrix-associated programs, and reciprocal communication between tumor and host cells.^92^ These effects are highly context-dependent because commonly used BET inhibitors act in both malignant and non-malignant compartments and frequently engage more than one BET family member. The immunological outcome therefore reflects the combined effects of tumor lineage, host immune competence, drug selectivity, dose, and treatment schedule.^27^

Pharmacological inhibition of BET bromodomains with JQ1, or I-BET762 disrupts BRD4-chromatin interactions, leading to a 70% reduction in *PD-L1* transcription in triple-negative breast cancer and prostate cancer models within 12 hours.^93,94^ JQ1 decreased *PD-L1* mRNA levels by 65% and increased HLA-ABC surface expression by ∼2.4-fold, improving antigen presentation and T cell cytotoxic responsiveness; quantitatively, FSCN-HiM significantly reduced *PD-L1* mRNA levels by 25% (*p* < 0.01) but demonstrated an insignificant increase in HLA-ABC expression in these experiments. Additionally, BRD4 blockade induced expression of ISGs and Th1 chemokines (CXCL9, CXCL10), which can contribute to the recruitment of CD8^+^ T cells, thereby reverting the “cold” tumor microenvironment.^28^

BET proteins also interact with both EZH2 and HDAC1 to balance the activation and repression of chromatin. Simultaneous inhibition of BET and EZH2 resulted in a greater than 2.7-fold increase in the expression of antigen-presenting genes, inducing durable immune memory that enabled tumor rejection in models of melanoma and glioblastoma.^95^ The contribution of individual BET family proteins to tumor immunogenicity and microenvironmental remodeling is strongly dependent on cancer lineage, cellular compartment, and experimental mode of perturbation. BRD4 has the most extensively characterized role and can act in both malignant and non-malignant cells. BRD4 controls checkpoint-ligand expression, interferon-responsive transcription, antigen-presentation pathways, and chemokine release in tumor cells.^28^ In immune and stromal compartments, BRD4-dependent transcription can influence macrophage polarization, inflammatory cytokine production, extracellular-matrix programs, and the recruitment or function of cytotoxic lymphocytes.^26^ BRD2 also contributes to immune-evasive signaling in selected settings; for example, BRD2-dependent *PD-L1* expression has been reported in cetuximab-resistant head and neck squamous cell carcinoma. By contrast, the immunological functions of BRD3 in cancer remain less clearly defined, and available studies predominantly implicate it in lineage-specific transcription, proliferation, and tumor-cell state rather than directly demonstrating immune-cell or stromal remodeling.^11^ BRDT is largely restricted to germ-cell biology and currently has limited evidence supporting a role in the immune microenvironment of common somatic malignancies.^96^ Accordingly, the feasibility of BET inhibition should be evaluated according to tumor-specific BET dependency, the relative contribution of tumor *versus* host cells, and whether the selected compound is pan-BET, isoform-selective, or bromodomain-selective.^97^

Moreover, despite the downregulating transcriptional activity of *PD-L1*, HDAC/BET dual inhibition led to an increase in acetylation at the *PD-L1* promoter in a manner that would generally suggest heightening MHC class II-mediated immune responses.^98^ Taken together, BET proteins regulate several complementary determinants of tumor immunogenicity, including checkpoint-ligand expression, interferon-responsive transcription, chemokine production, and MHC-dependent antigen presentation. The magnitude and direction of these effects vary according to the BET family member, tumor lineage, and cellular context. Table 3 summarizes these context-dependent relationships and distinguishes direct transcriptional effects from broader changes in immune signaling.

**Table 3:** BET proteins as modulators of tumor immunogenicity

BET type	3D structure	Cancer type	Molecular pathway	Molecular target	IC_50_ value	References
**BRD2**	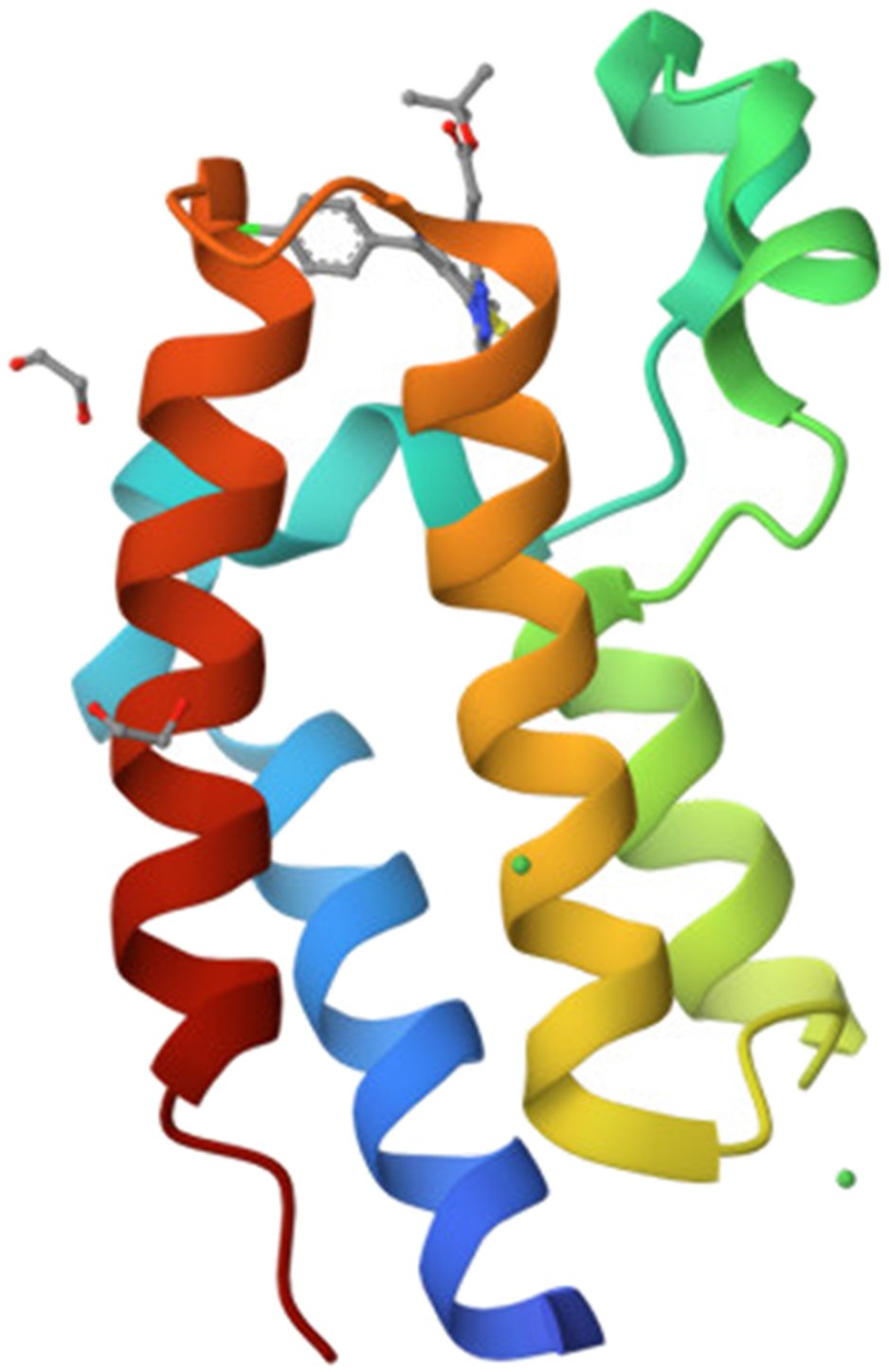 **PDB: 3ONI**	HNSCC	*PD-L1* upregulation *via* BRD2 in cetuximab/cisplatin resistance	*PD-L1*	NA	99
		Leukemia	c-*MYC* degradation (*via* pan-BET PROTAC dBET1)	c-*MYC*	NA	100
		Lung cancer	SRC/MAPK/AP-1/*PD-L1* signaling	JUN, FOS	NA	101
		Melanoma	MEK–ERK1/2 pathway	BRAF/MEK	NA	102
		Ovarian cancer	PI3K-AKT pathway	PI3K-AKT	NA	103
		Prostate cancer	AR–BRD2 interaction at hormone-responsive genes (H2A.Z/H4-acetylation-dependent recruitment)	AR	NA	104
**BRD3**	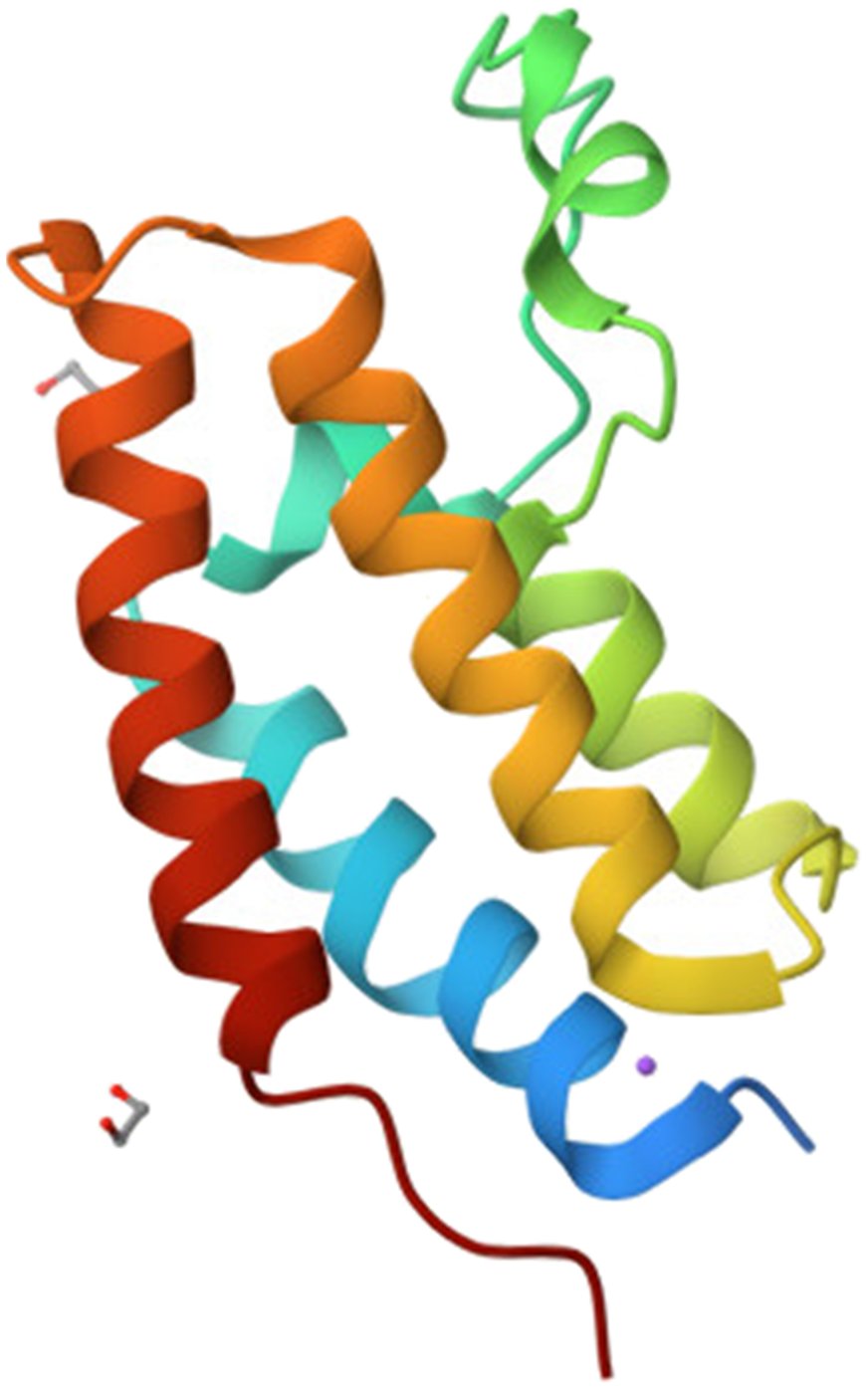 **PDB: 2NXB**	Ovarian cancer	PI3K-AKT pathway	PI3K-AKT	NA	103
		Renal carcinoma	mTOR signaling	mTOR, HLA-ABC	NA	105
**BRD4**	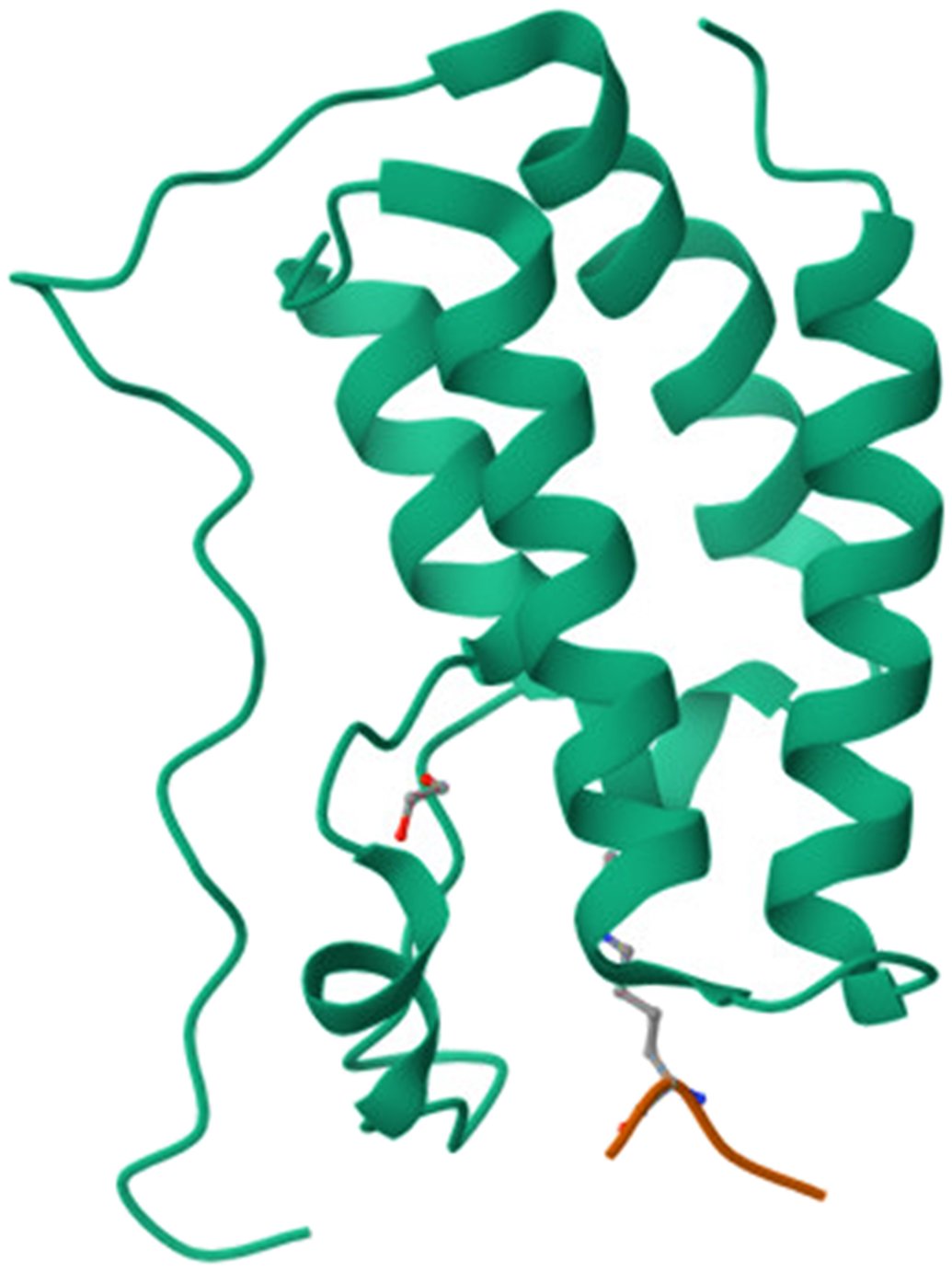 **PDB: 3JVK**	NSCLC, hematological cancers	General BRD4-targeting rationale (review)	BRD4	NA	106
		Glioblastoma	Super-enhancer disruption *via* PROTAC-mediated degradation	SDC1, c-*Myc*	0.47–3.68 μM (cell-line dependent)	107
		AML	*MYC* transcriptional suppression	*Myc*/NRF2	NA	108
		Breast cancer	BRD4/*MYC* regulation	*MYC*, *PD-L1*	3.0 μM	109, 110
		Colorectal cancer	Chromatin remodeling	JAK2-STAT3	NA	111, 112
		Head and neck squamous cell carcinoma	MHC class I/*PD-L1* regulation	*PD-L1*, HLA-A	NA	25
		Lymphoblastic leukemia	*Myc*-pathway	Inhibits T-cell	NA	113
		NSCLC	IRF1 axis	*PD-L1*, IRF1	NA	114
		Prostate cancer	MHC class I expression	*PD-L1*, HLA-A	NA	94
		Triple-negative breast cancer	NF-κB/RelA signaling	RelA	NA	115
		Triple-negative breast cancer	PD-1/*PD-L1* pathway	*PD-L1*	NA	30

### 4.1. Regulation of *PD-L1* and antigen presentation

Although suppression of *PD-L1* and restoration of MHC-dependent antigen presentation provide an important rationale for BET–ICI combinations, these mechanisms represent only part of the broader remodeling induced by BET inhibition.^116^

### 4.2. Myeloid-cell recruitment and macrophage polarization

Myeloid populations are major determinants of resistance to immune checkpoint blockade, and several studies indicate that BET inhibition can alter their recruitment and functional state. Tumor-associated macrophages often adopt an M2-like, tissue-remodeling phenotype that is associated with production of immunosuppressive cytokines, competent antigen-presenting capacity, and inhibition of cytotoxic lymphocytes. The transcriptional regulation establishes these processes, and BRD4-dependent transcription is required for the maintenance of these programs in some tumors. AZD5153 decreased M2-like macrophage polarization, altered MAF-dependent transcription, increased CPI phenotype-associated pro-inflammatory cytokine secretion, and augmented pharmacologic activation of CD8^+^ T cells in preclinical models of high-grade serous ovarian cancer. In this same study, responsiveness to *PD-L1* blockade was also improved in a three-dimensional tumor-immune model as compared to two-dimensional cultures, implicating changes in the myeloid compartment from BRD4 inhibition in many of its mediating effects rather than solely through tumor-cell *PD-L1* suppression.^26^

BET inhibition can inhibit the bidirectional crosstalk between malignant cells and macrophages. AZD5153 inhibited BRD4-dependent transcription in cancer cell–macrophage interactions, which reduced a tumor-supportive paracrine circuit. This finding is important because macrophages are not merely passive recipients of tumor-derived signals; they can reinforce oncogenic transcription, tumor survival, invasion, and resistance through reciprocal cytokine and growth-factor signaling. Thus, inhibition of BET-dependent tumor–macrophage crosstalk may reduce both immunosuppression and non-immune trophic support within the tumor microenvironment.^116^

BET inhibition can also affect myeloid-derived suppressor cells. In syngeneic and spontaneous tumor models, pharmacological BRD4 inhibition with JQ1 or the dual PI3K/BRD4 inhibitor SF2523 reduced myeloid-derived suppressor-cell infiltration, restrained immunosuppressive macrophage polarization, restored CD8^+^ T-cell activity, and promoted antitumor immune responses. These findings suggest that BET-dependent transcription contributes to a broader myeloid suppressive network, although the effects of dual-target agents cannot be attributed exclusively to BRD4.^29^

### 4.3. T-cell infiltration, activation, and exhaustion

BET inhibition may also influence the abundance, localization, and functional state of tumor-infiltrating lymphocytes. In Rb-deficient prostate cancer, JQ1 increased immune-cell infiltration through tumor-cell activation of STING, NF-κB, and type I interferon signaling. The resulting antitumor effect depended on macrophages and T cells and enhanced sensitivity to immune checkpoint blockade. This model illustrates how BET inhibition can convert a genomically defined, poorly infiltrated tumor into a more inflamed state through innate immune signaling rather than solely through checkpoint-ligand suppression.^117^ In colorectal cancer models, JQ1 increased MHC class I expression, enhanced cytotoxic T-cell activity, reduced intratumoral regulatory T-cell infiltration, and improved the antitumor efficacy of PD-1 blockade in immunocompetent mice. By contrast, JQ1 showed little comparable antitumor activity in T-cell-deficient hosts, supporting an important contribution from adaptive immunity. These results indicate that BET inhibition may enhance checkpoint therapy through simultaneous effects on tumor-cell recognition, effector-cell activity, and regulatory T-cell abundance.^118^ The effect of BET inhibition on T cells is nevertheless not uniformly stimulatory. BET proteins participate in transcriptional programs required for lymphocyte activation, proliferation, differentiation, and cytokine production. Consequently, sustained or high-intensity inhibition may suppress activated immune cells even when tumor-cell immunogenicity is improved. This duality reinforces the need for intermittent or pharmacodynamically guided dosing that produces sufficient tumor and myeloid reprogramming without compromising effector-cell fitness.^119^

### 4.4. Cytokine, chemokine, and innate immune signaling

BET proteins also regulate cytokine and chemokine networks that determine whether immune cells can enter and remain functional within tumors. Depending on cellular context, BET inhibition can suppress tumor-supportive mediators such as IL-6, CXCL12, and selected NF-κB-dependent inflammatory programs, thereby reducing recruitment or maintenance of suppressive myeloid and stromal populations. In other settings, BET inhibition can activate innate immune pathways. In Rb-deficient prostate cancer, JQ1 induced DNA-damage-associated STING activation, non-canonical NF-κB signaling, and type I interferon production, which promoted T-cell and macrophage infiltration and sensitized tumors to checkpoint blockade.^117,120^

These apparently divergent effects reflect the fact that BET proteins can sustain both tumor-promoting inflammation and antitumor interferon responses. The net outcome depends on which transcriptional program is dominant in a given tumor. BET inhibition may be favorable when it suppresses chronic IL-6-, CXCL12-, or myeloid-supportive signaling while preserving or inducing CXCL9, CXCL10, type I interferons, and other mediators required for lymphocyte recruitment. Conversely, broad inhibition that attenuates productive interferon signaling could reduce immune-cell trafficking or antigen presentation. Measurement of cytokine and chemokine changes should therefore be incorporated into preclinical evaluation of BET–ICI combinations.^121,122^

### 4.5. Stromal and cancer-associated fibroblast interactions

Non-immune stromal cells represent another BET-regulated component of the tumor microenvironment. Cancer-associated fibroblasts can promote extracellular-matrix deposition, physical immune exclusion, tumor-cell survival, and resistance to targeted therapies. In pancreatic cancer models, BET bromodomain inhibition reduced cancer-associated fibroblast content, indicating that part of the antitumor effect may arise through suppression of the desmoplastic compartment.^123^ Stromal cells can also modify tumor-cell sensitivity to BET inhibitors. Cancer-associated fibroblast-derived signals have been shown to induce BRD4 phosphorylation and resistance to BET inhibition, demonstrating that the microenvironment can both be remodeled by BET-targeted therapy and determine the tumor's response to it. This reciprocal relationship is especially relevant in fibroblast-rich malignancies such as pancreatic cancer, where dense stroma, myeloid suppression, and limited T-cell penetration may restrict the efficacy of a simple BETi–ICI combination.^112^ Therefore, assessment of BET inhibition in stromal-rich cancers should include changes in fibroblast abundance and state, extracellular-matrix architecture, vascular accessibility, myeloid recruitment, and lymphocyte penetration. Tumor-size reduction alone cannot determine whether a BET inhibitor has produced meaningful immune sensitization.^22^

### 4.6. Context-dependent and potentially adverse immune effects

The effects of BET inhibition on the tumor microenvironment should not be interpreted as uniformly beneficial. Pan-BET inhibitors act simultaneously on tumor cells, macrophages, dendritic cells, fibroblasts, hematopoietic progenitors, and lymphocyte subsets. A reduction in suppressive macrophages or regulatory T cells may favor checkpoint activity, whereas excessive inhibition of inflammatory transcription or lymphocyte proliferation could impair immune effector function. Moreover, results obtained with JQ1, AZD5153, or dual-target compounds cannot automatically be assigned to BRD4 alone because these agents differ in isoform selectivity, bromodomain preference, pharmacokinetics, and off-target activity.^26,124,125^ The feasibility of BET–ICI therapy should therefore be evaluated using a compartment-resolved biomarker strategy. Relevant measurements include tumor-cell *PD-L1* and MHC expression, STING and interferon activity, CXCL9/CXCL10 production, CD8^+^ T-cell infiltration and function, regulatory T-cell abundance, macrophage polarization, myeloid-derived suppressor-cell infiltration, fibroblast and extracellular-matrix signatures, and circulating hematologic toxicity. Such analyses will help determine whether BET inhibition has produced productive immune remodeling, neutral transcriptional suppression, or an unfavorable loss of immune-cell fitness.^126,127^

BRD4 illustrates why the cellular source of BET activity matters when evaluating therapeutic feasibility. Tumor-cell BRD4 can sustain *PD-L1* expression, suppress antigen presentation, and support oncogenic transcription, whereas BRD4 activity in myeloid or stromal cells can maintain immunosuppressive and inflammatory programs. In high-grade serous ovarian cancer, AZD5153 reduced M2-like macrophage polarization and improved antitumor immune activity, indicating that BET inhibition can act beyond the malignant cell compartment.^26^ In other settings, BRD4-dependent extracellular-matrix and inflammatory transcription may contribute to an immune-excluding microenvironment. Conversely, clinical data have also indicated that broad BET inhibition can reduce circulating monocytes or immune-effector signatures, underscoring that host-cell effects may be either beneficial or detrimental depending on dose, schedule, and compound selectivity.^128^

## 5. BET inhibition sensitizes tumors to checkpoint blockade

**Fig. 4:**
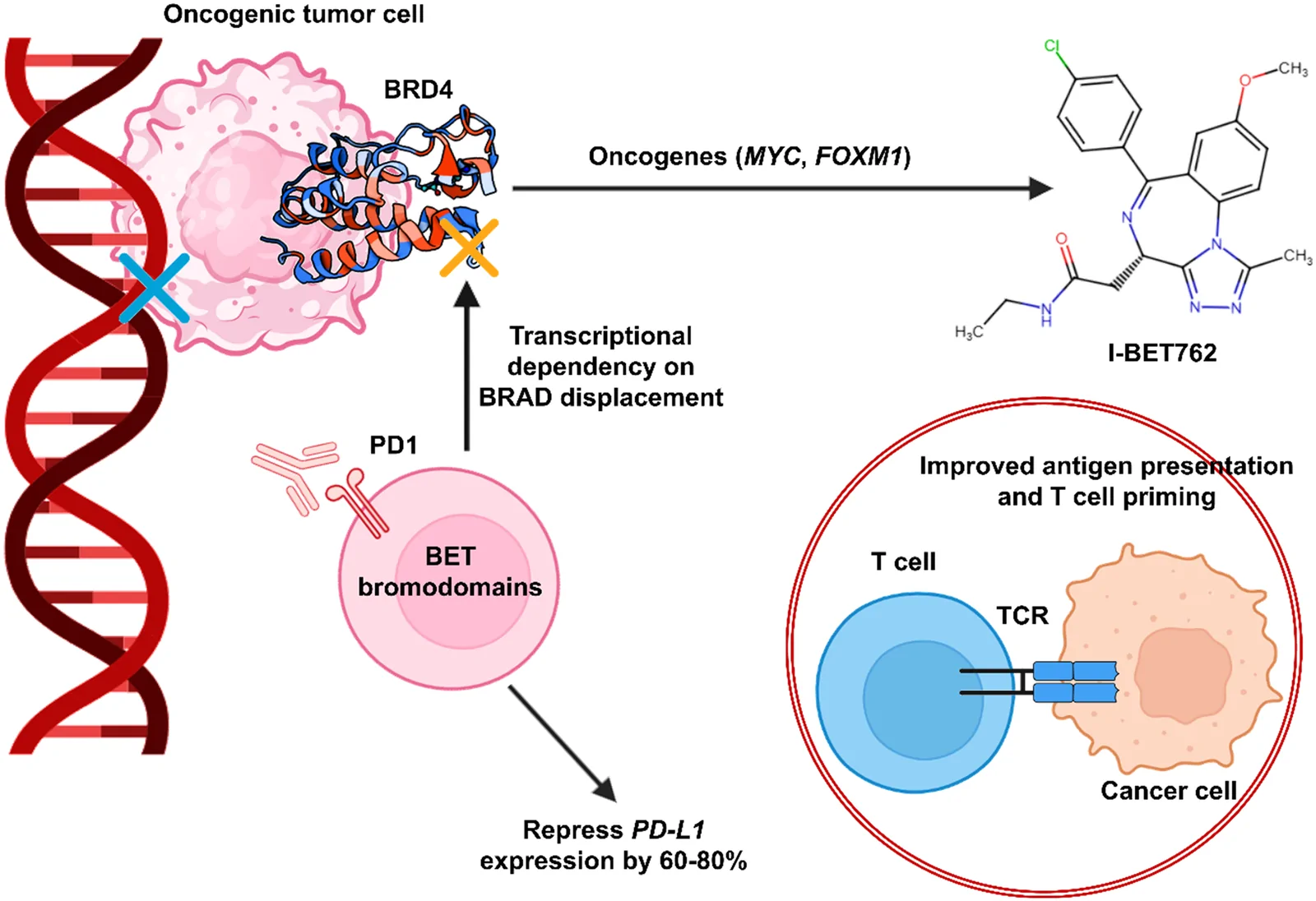
BET inhibition sensitizes tumors to checkpoint blockade. Partially created based on (https://www.biorender.com/).

The transcriptional and immunological effects described above provide the basis for combining BET inhibitors with immune checkpoint blockade. Preclinical studies suggest that BET inhibition can increase the responsiveness of selected tumors to PD-1, *PD-L1*, or CTLA-4 blockade by simultaneously modifying tumor-cell-intrinsic transcription and the composition of the tumor immune microenvironment. Inhibitors such as JQ1 and I-BET762 can block these transcriptional programs and decrease *PD-L1* expression by 60–80% across many models, leading to improved antigen presentation and T-cell priming.^27,129^ In an immunocompetent murine TNBC model, JQ1 and anti-PD-1 treatment led to greater tumor-growth inhibition than either treatment alone and were associated with approximately twofold more intratumoral CD8^+^ T cells, indicating that the combination response is at least partly immune-mediated.^130^ In a *KRAS*-mutant model of NSCLC, BET bromodomain inhibition decreased BRD4-dependent transcription of *PD-L1*, and concomitant blockage in PD-1 improved tumor control and survival over monotherapy.^131^ Functionally, BET inhibition also suppresses interleukin-6 and CXCL12, reducing MDSC infiltration and rescuing IFN-gamma-driven immune activation, as elucidated in Fig. 4.^28^

**Fig. 5:**
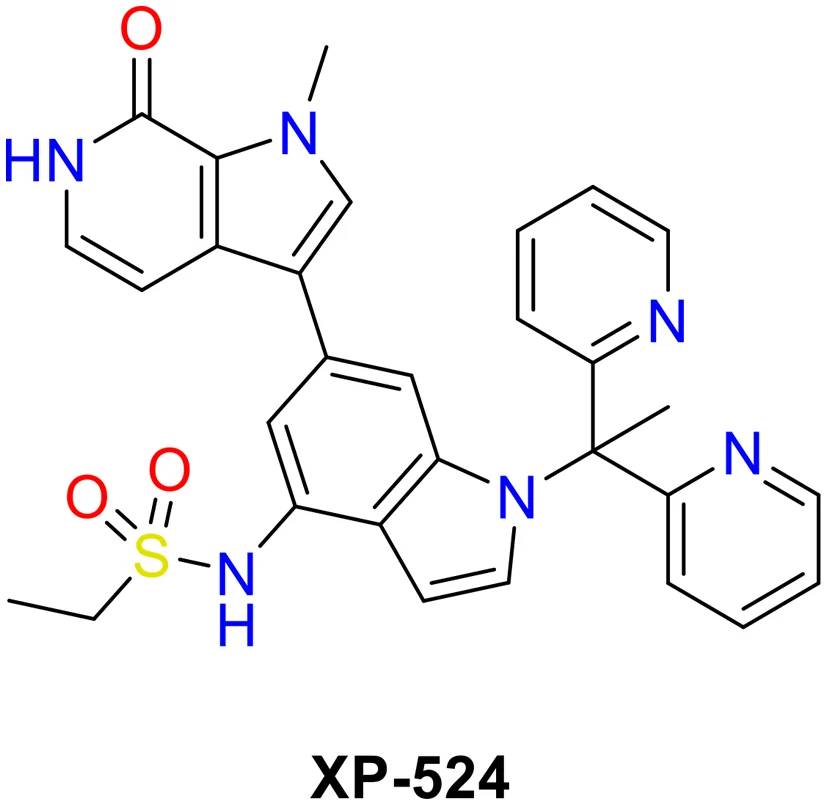
Structure of XP-524.

Recent studies have extended this paradigm: the dual BET/EP300 inhibitor XP-524 (**4**) (Fig. 5) decreased *KRAS* and *PD-L1* co-expression in pancreatic cancer, with concomitant tumor burden reduction of 67% and enhanced anti-CTLA-4 therapy effectiveness.^132^

However, the interpretation of *in vivo* BET inhibitor studies is highly dependent on the experimental model used. Indeed, more informative systems to evaluate the capacity for cooperation between BET inhibition and immune checkpoint blockade consist of syngeneic models in an immunocompetent context that preserve functional innate and adaptive immune compartments. In these models, effects on intratumoral CD8^+^ T-cell infiltration, interferon-responsive signaling, antigen presentation, myeloid-cell composition, and adaptive immune resistance can be associated with treatment outcomes.^28^ Traditional human tumor xenografts in immunodeficient hosts focused on tumor-cell-intrinsic responses, including inhibition of oncogenic transcription and other target engagement as well as inhibition of tumor growth, pharmacokinetics, and tolerability. While xenograft studies provide direct evidence of antitumor activity, they cannot be used alone to prove immune-mediated synergy with PD-1, *PD-L1*, or CTLA-4 blockade. In this regard, data from syngeneic models should be weighted more heavily than xenograft results when evaluating the immunological rationale for BET–ICI combinations; in contrast, xenograft findings serve as additional evidence of tumor-intrinsic efficacy.^27^ Systems biology modeling also provides molecular support for this synergy, predicting that BETi corrects cytokine feedback loops that maintain T-cell activation during checkpoint blockade.^133^ Collectively, these studies support pharmacological cooperation between BET inhibition and checkpoint blockade across selected preclinical settings. However, the available evidence remains heterogeneous with respect to tumor model, BET inhibitor, checkpoint target, dosing schedule, and immune endpoint. Direct comparisons across immunocompetent models, together with pharmacodynamic confirmation of BET target engagement, will be required to identify the tumor contexts most likely to benefit from this strategy.

## 6. Molecular design, binding affinity, and SAR of BET inhibitors

The identification of bromodomain and extraterminal (BET) inhibitors exemplifies the achievements of bioorganic chemistry and specialist disciplines such as structure-based drug design and optimization of inhibitor–protein contacts.^120^ BET proteins, including BRD2, BRD3, and BRD4, possess two bromodomains (BD1 and BD2), which can read acetylated lysines on histones H3 and H4. Functional conservation of the asparagine–acetyl-lysine hydrogen bond network in the binding pocket was first observed from crystallography studies and led to the development of triazolobenzodiazepine scaffolds exemplified by JQ1 and I-BET762.^134^ JQ1 possesses nanomolar affinity (KD ≈ 50 nM) for BRD4-BD1 and preferentially competes with acetyl-lysine recognition, resulting in wide-scale transcriptional inhibition of *MYC* and *PD-L1*. SAR optimization studies revealed that substituting the diazepine nitrogen with an oxygen atom improved potency by 2.5-fold, as well as solubility and metabolic stability.^135^ Both the benzotriazepine analogues, including MS436, and their binding affinity were rescued to KD = 18 nM, driven by π–π stacking with WPF shelf residues in BRD4. Although most dual BET-directed compounds were initially developed to enhance antitumor efficacy by simultaneously inhibiting complementary oncogenic pathways, many of the targeted signaling networks also regulate tumor immunogenicity, immune-cell recruitment, stromal remodeling, and resistance to immune checkpoint blockade. Consequently, the therapeutic value of dual BET inhibitors extends beyond direct tumor-cell cytotoxicity and may include modulation of the tumor immune microenvironment. The following subsections therefore summarize not only the medicinal chemistry of each dual-target strategy but also its potential immunological relevance and compatibility with immune checkpoint inhibitor (ICI)-based combination therapy.^29,136^

**Fig. 6:**
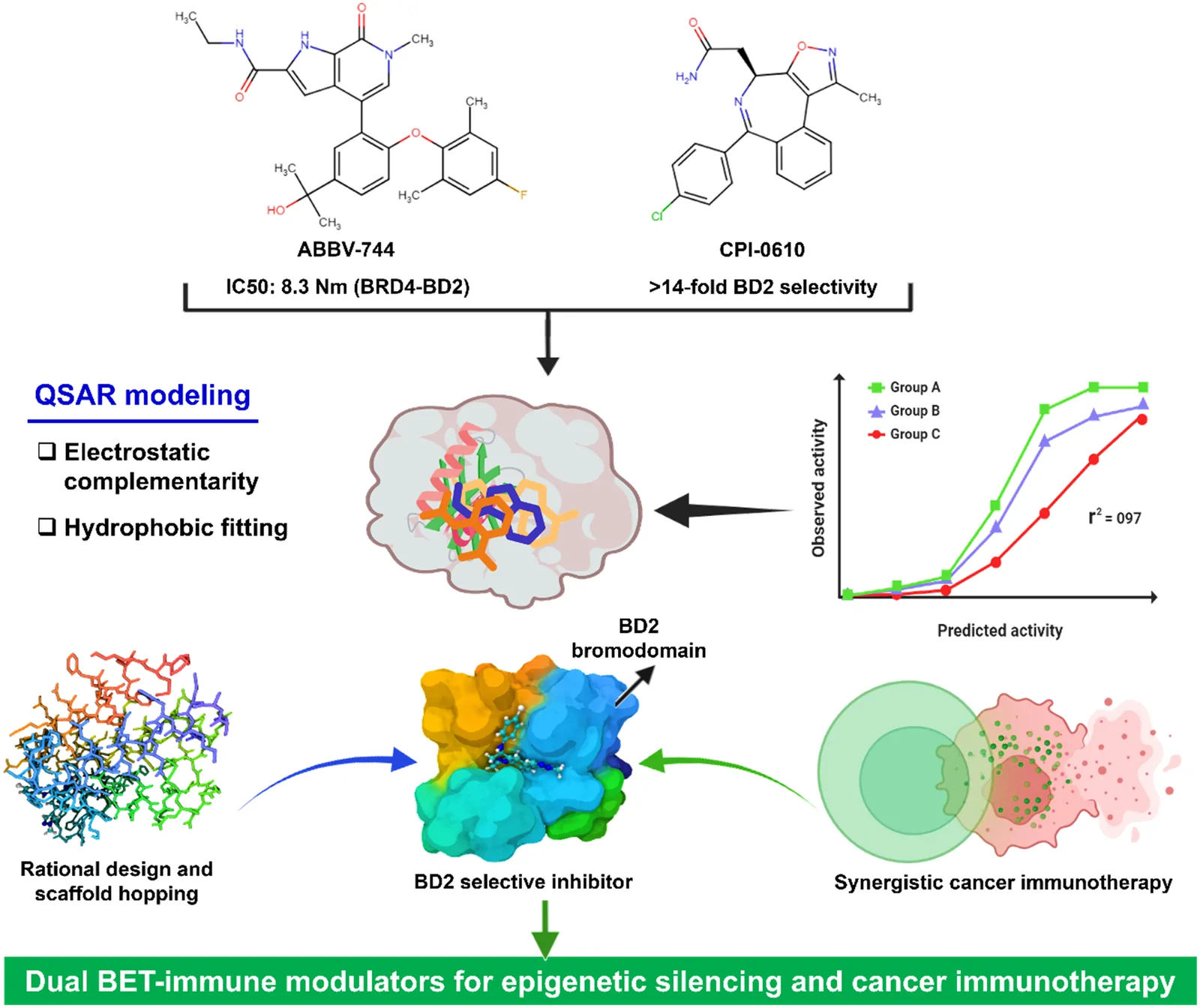
Next-generation inhibitors for BD2 selectivity to lower hematologic toxicity. Partially created based on (https://www.biorender.com/).

Next-generation inhibitors, such as ABBV-744 and CPI-0610, provided BD2 selectivity driven by the need to lower hematologic toxicity. ABBV-744 had an IC_50_ of 8.3 nM for BRD4-BD2 over 118 nM for BD1 (>14-fold selectivity).^137^ Computational QSAR studies also supported these interactions, based on electrostatic complementarity and hydrophobic fitting, in a way that predictive *r*^2^ values > 0.95 corroborate the robustness of the SAR.^138^ The bioorganic chemistry of BET inhibitors demonstrates that rational design, scaffold hopping, and domain-selective engineering have resulted in compounds with precisely calibrated affinity and specificity. These findings connect chemical synthesis with molecular pharmacology and provide a rationale for the development of dual BET–immune modulators to synergize epigenetic silencing with cancer immunotherapy, as demonstrated in Fig. 6.^139^

### 6.1. Synthesis of the triazolobenzodiazepine scaffold (JQ1 analogues)

Chemical probes derived from (+)-JQ1 have diversified into two principal categories. The first includes label-free probes, while the second comprises labeled variants. Together, these developments illustrate the expanding versatility of (+)-JQ1 scaffolds in chemical biology and drug-discovery research.^140^

#### Label-free chemical probes

**Fig. 7:**
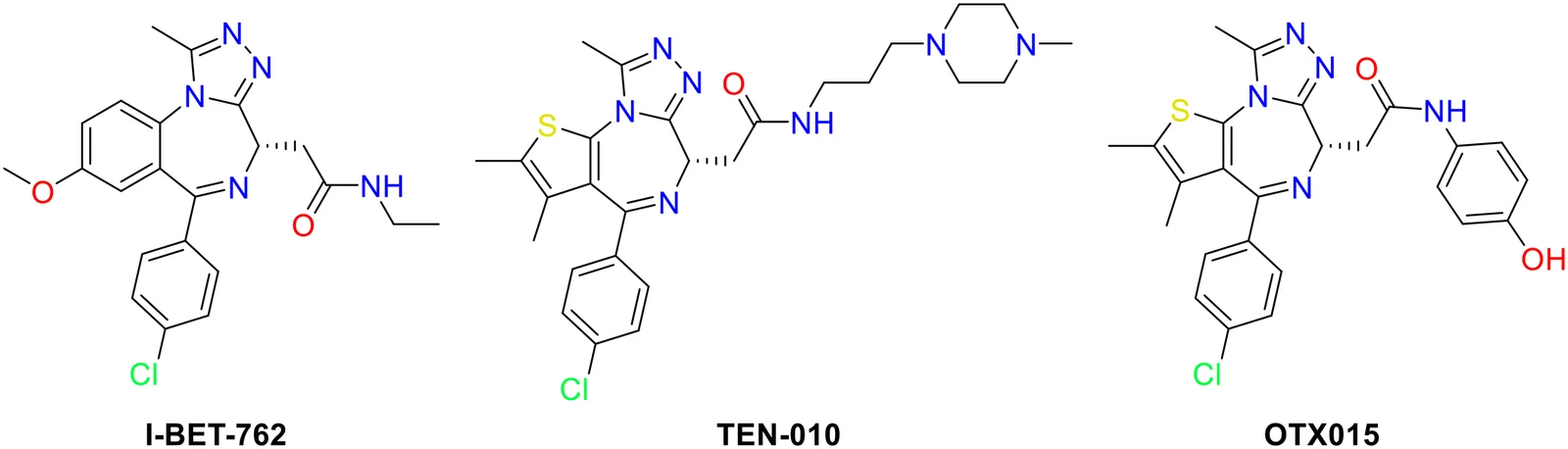
Structures of I-BET762, TEN010, and OTX015.

Numerous label-free (+)-JQ1-derivatized probes have been reported to improve upon the pharmacological properties of the original compound. Key examples include thienotriazolodiazepine inhibitors like I-BET762, TEN010, and OTX015, which demonstrated nanomolar potency against BET proteins and advanced into human clinical studies. This group also includes dual-targeting inhibitors, often linking (+)-JQ1 with a second active inhibitor to address limitations such as resistance^139,141^ (Fig. 7).

#### Labelled chemical probes

##### Fluorescent probes

**Fig. 8:**
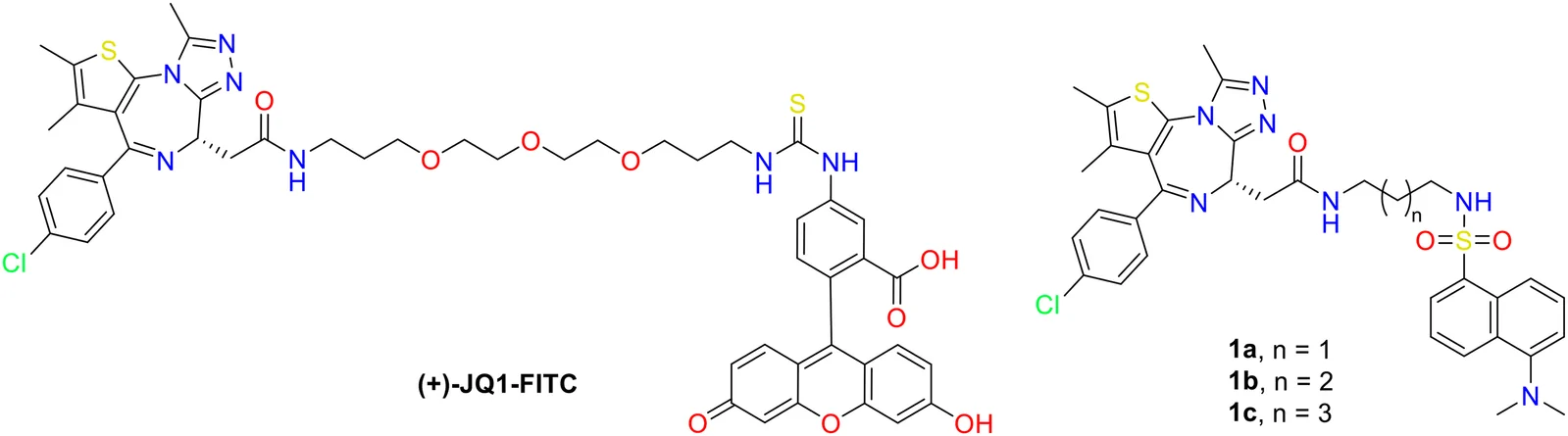
Structures of fluorescent probe (+)-JQ1-FITC and probes 1a–c.

An example is the fluorescent probe (+)-JQ1-FITC, which exhibits high affinity for BRD4(BD1) and BRD4(BD2) and was utilized to validate a TR-FRET-based platform.^142^ Furthermore, environment-sensitive probes 1a–c, designed using a dansyl amide fluorophore, were employed to label and study the distribution of BET family proteins in tumor cells and tissues^143^ (Fig. 8).

##### Photoaffinity labeling

**Fig. 9:**
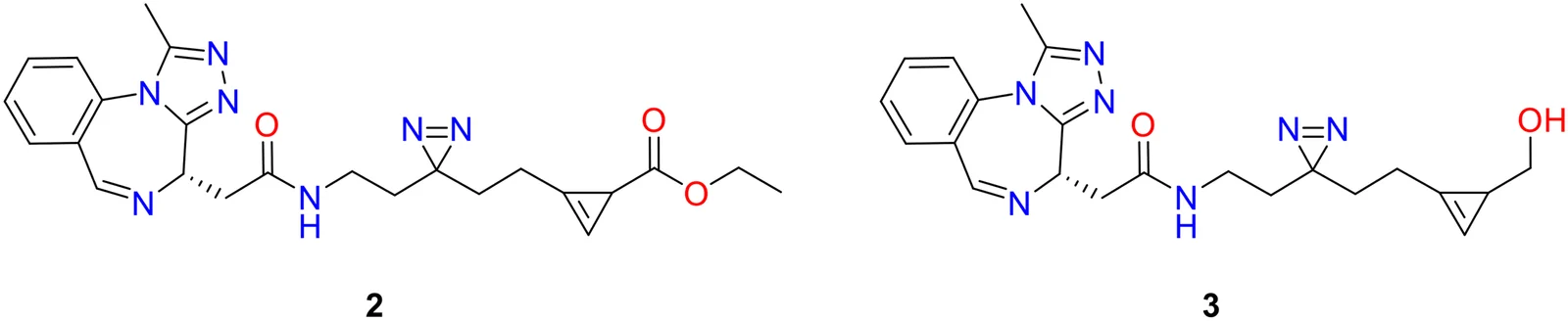
Structures of chemical probes 2 and 3.

Photoaffinity labeling (PAL) is a covalent labeling method in which photoaffinity probes contain photoreactive groups, such as diazirines, aryl azides, or diaryl ketones. Novel BRD4 targeting chemical probes 2 and 3 have been developed to enable copper-free bioorthogonal chemistry for target identification^144^ (Fig. 9).

### 6.2. Design of a dual-action BET-HDAC hybrid inhibitor

**Fig. 10:**
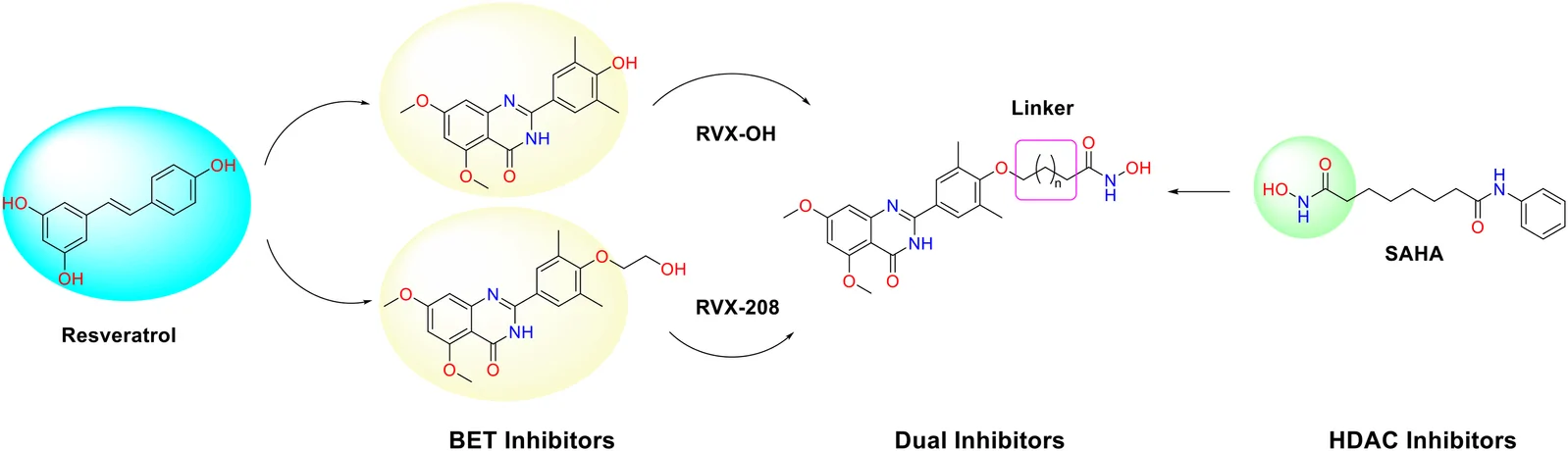
Schematic diagram of construct novel dual BRD4/HDAC inhibitors.

**Scheme 1:**
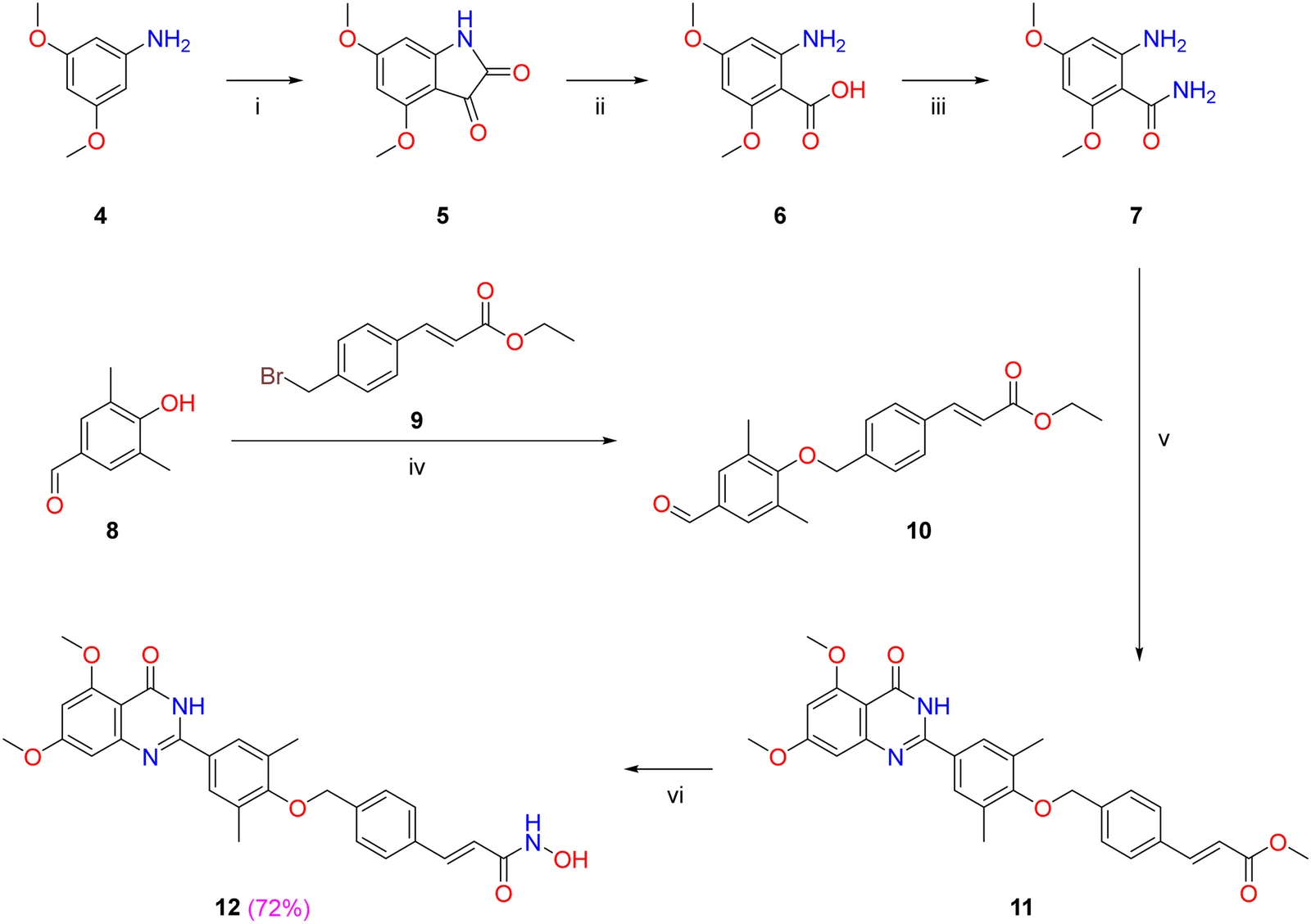
Synthesis of the target 12. Reagents and conditions: (i) HCl (g), ether, 0 °C, 2 h, oxalyl chloride, 70 °C, 1.5 h, MeOH, reflux, 1 h; (ii) NaOH (33% in water), H_2_O_2_ (30% in water), 1 h, 70 °C; (iii) EDC, HOBT, NMM, THF, 4 h, NH_3_ (g), 1 h, r.t.; (iv) K_2_CO_3_, MeCN, reflux, 24 h; (v) PTSA, NaHSO_3_, DMAc, 120 °C, 16 h; (vi) 50% NH_2_OH aq., NaOH, DCM/MeOH (1 : 2), r.t.

Bromodomain and extra-terminal (BET) and histone deacetylase (HDAC) are influential targets in drug discovery and development. The analogous properties of the BET (bromodomain and extra terminal) family and HDACs, such as their roles in epigenetic mechanisms, enable the design of dual hybrids with sub-nanomolar cytotoxic and enzymatic activities.^145,146^ Mingfeng Shao *et al.* reported the design of a dual-action BET/HDAC hybrid inhibitor using a structure-based approach. This strategy was motivated by the observation that BET and HDAC inhibitors synergize to kill *Myc*-induced murine lymphoma cells.^147^ The design process involved selecting RVX-OH and RVX-208, derivatives of resveratrol (3,4′,5-trihydroxy-transstilbene), which have good biological effects on the BET proteins. This core was subsequently fused to a hydroxamate moiety, which is the more potent zinc-binding group (ZBG) important for chelation of the zinc ion that resides in the HDAC active site. Different linkers were employed to bind the ZBG to the free hydroxyl group of the phenol ring. This rational method successfully generated novel compounds, such as 12, which functioned as potent dual inhibitors of **BRD4** and HDAC1 (Fig. 10). The synthetic procedure for compound 12 is depicted in Scheme 1. From an immunological standpoint, co-inhibition of BET proteins and histone deacetylases represents a particularly salient strategy given the fact that both target classes regulate chromatin accessibility, inflammatory transcription, and antigen-presentation pathways. The impact of HDAC inhibition on MHC class I expression, tumor-antigen presentation, interferon-responsive transcription, and T-cell infiltration is well established in many malignancies. When combined with BET inhibition, these effects may simultaneously dampen immune-evasive transcriptional programs and sensitize tumor immunogenicity, facilitating a permissive environment for PD-1/*PD-L1* blockade. These novel BET–HDAC dual inhibitors are promising next-generation therapeutic agents for overcoming epigenetically mediated immune resistance, but their clinical validation is still limited.^147,148^

### 6.3. Development of a quinazolinone-based BRD4 inhibitor

**Fig. 11:**
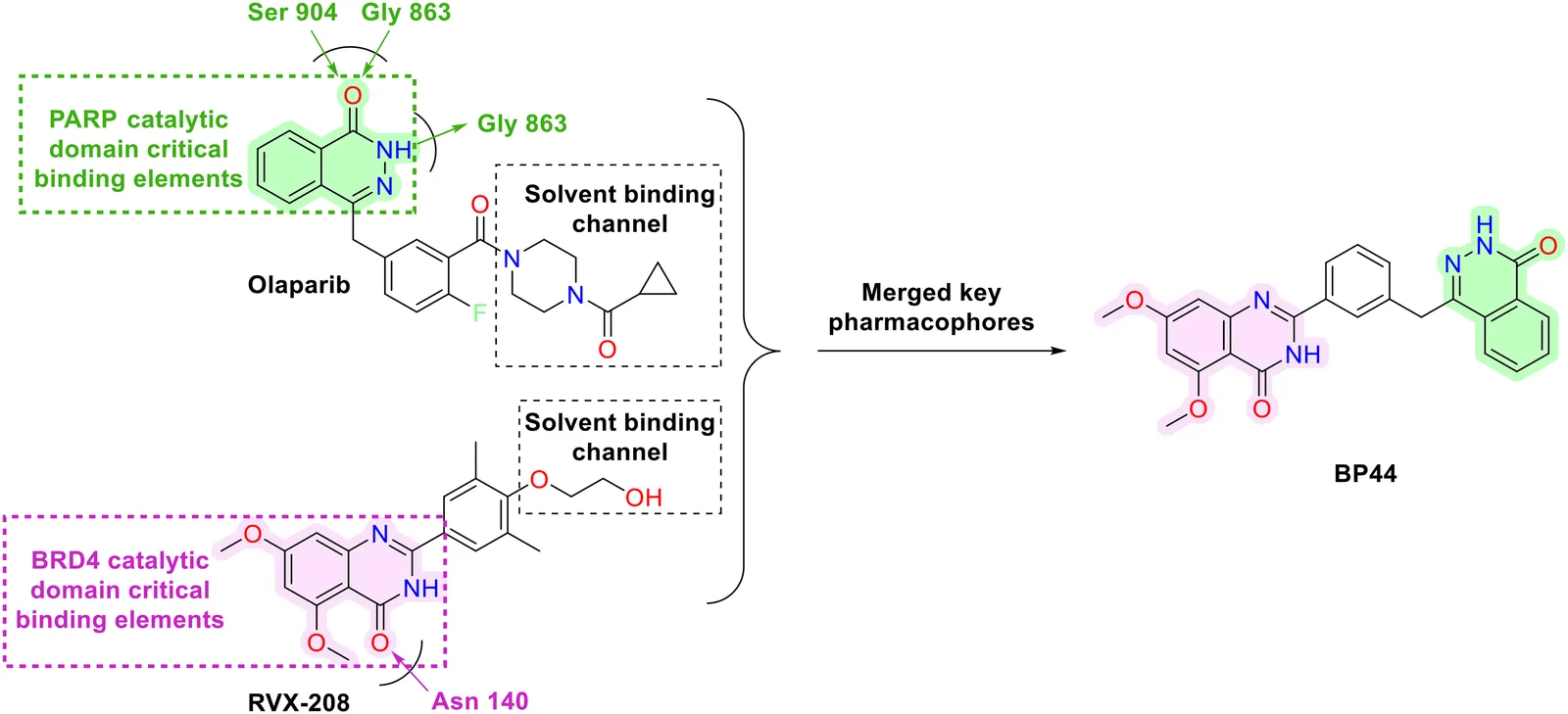
Design of dual BRD4-PARP1 inhibitors.

**Scheme 2:**
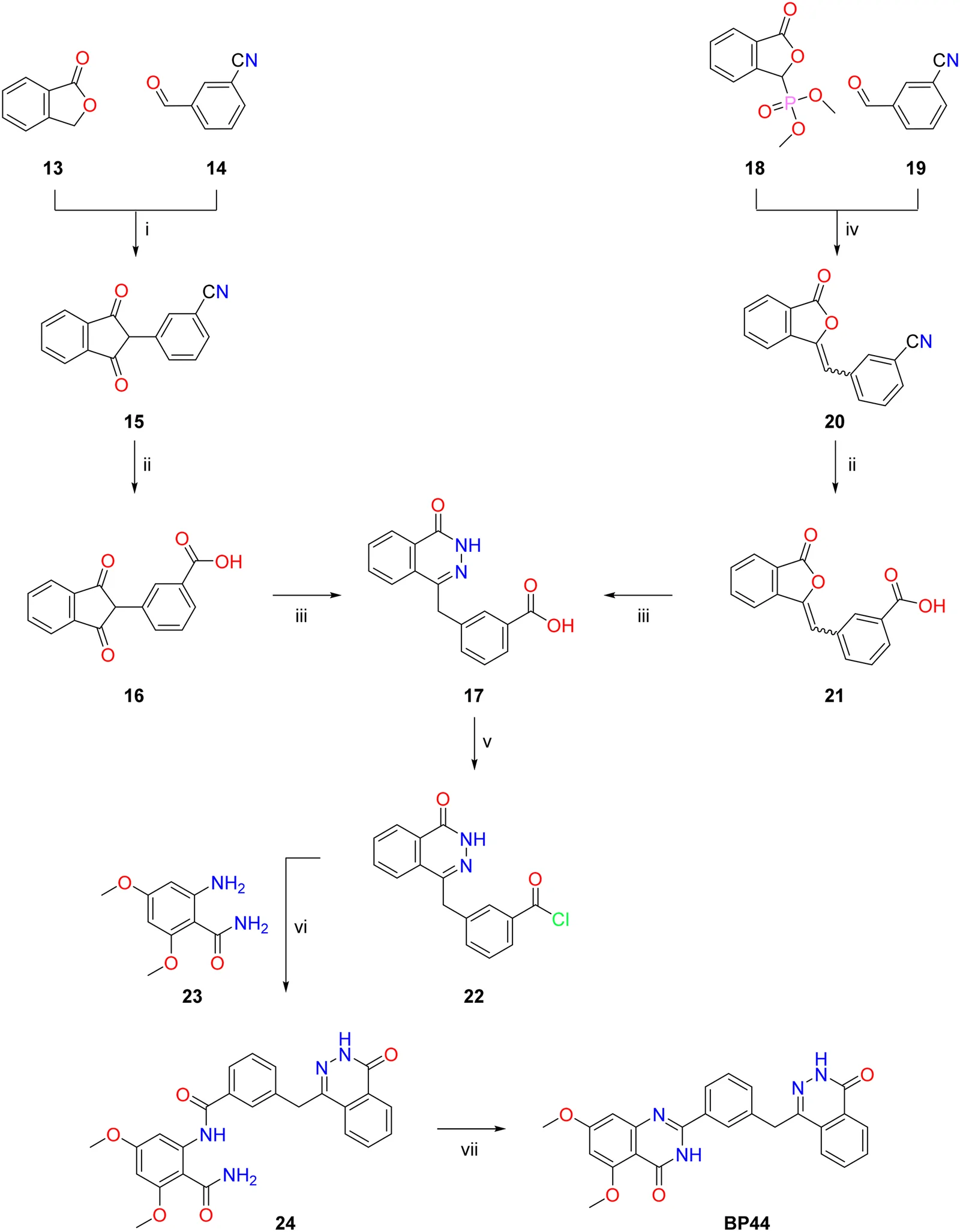
Synthesis of compound **BP44**. Reagents and conditions: (i) EA, NaOMe, MeOH, r.t., 3 h; (ii) NaOH, H_2_O, 100 °C, 6–10 h; (iii) N_2_H_4_, H_2_O, 100 °C, 6–10 h; (iv) Et_3_N, THF, r.t., 5 h; (v) SOCl_2_, 80 °C, 2.5 h; (vi) dry DCM, pyridine, 0 °C, 4–8 h; (vii) NaOH, H_2_O, 100 °C, 8–10 h.

Jifa Zhang *et al.* developed quinazolinone-based BRD4 inhibitors focused on designing dual BRD4-PARP1 inhibitors to exploit synthetic lethality, particularly for triple-negative breast cancer (TNBC). This structure was selected because the quinazoline ring part was identified as the key fragment of BRD4 binding based on the co-crystal analysis of the BRD4 inhibitor RVX-208. Researchers employed a pharmacophore fusion strategy, linking the BRD4-binding quinazolinone fragment with the phthalazine moiety of the PARP1 inhibitor Olaparib. This optimization yielded compound **BP44**, which showed high selectivity and potent inhibitory activity against both BRD4 and PARP1 (Fig. 11). The synthetic pathway for compound BP44 is shown in Scheme 2. Molecular docking confirmed that the quinazolinone ring of BP44 formed a key hydrogen bond interaction with Asn433/Asn140 in the BRD4 BD1/BD2 domains.^149^

**Fig. 12:**
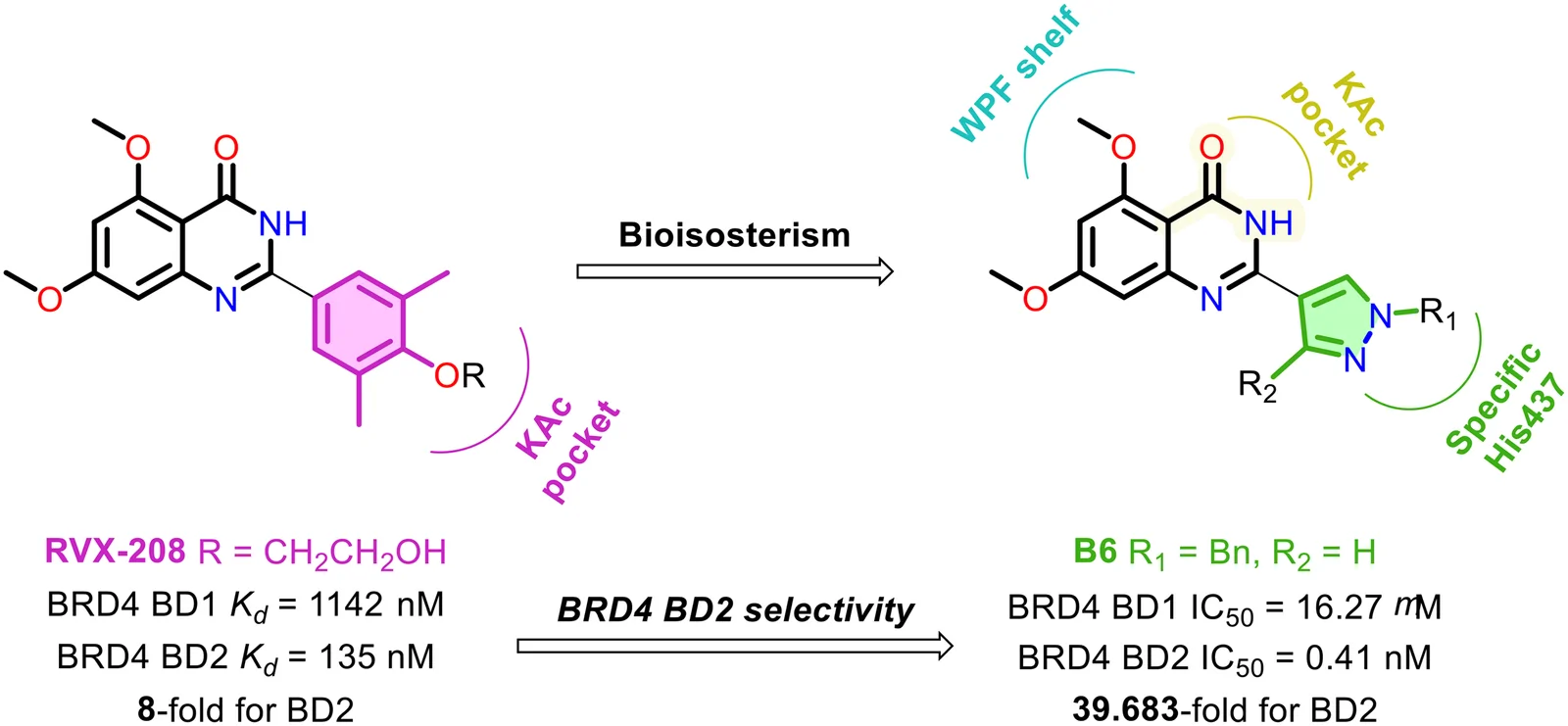
Design of BRD4 inhibitors *via* a bioisosterism approach.

**Scheme 3:**
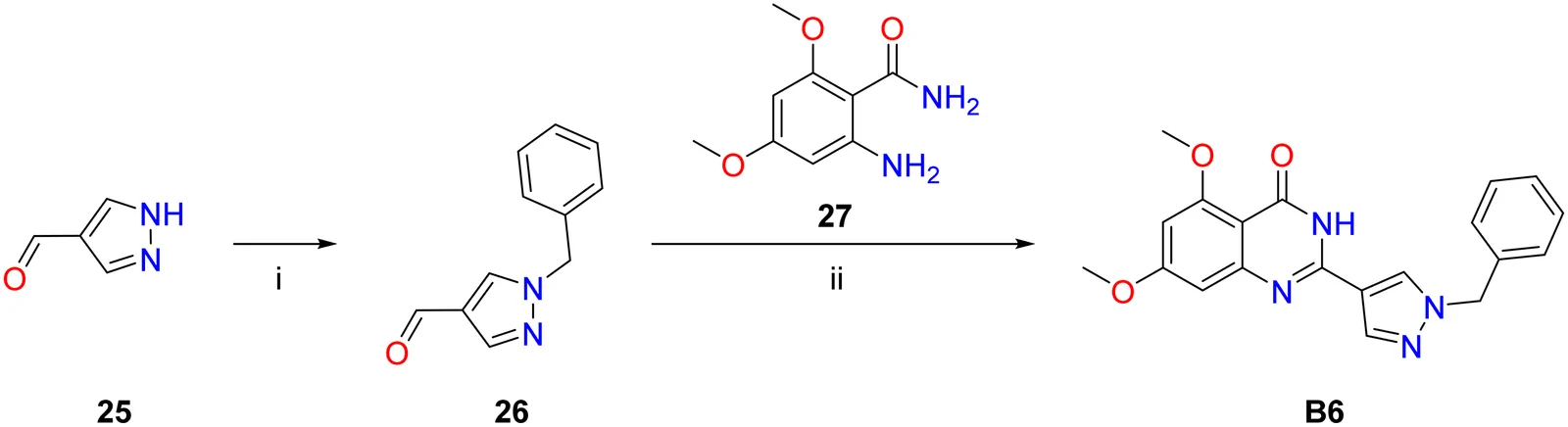
Synthesis of compound B6. Reagents and conditions: (i) bromides, Cs_2_CO_3_, anhydrous MeCN, 80 °C, overnight; (ii) I_2_, EtOH, reflux, 3 h.

On the other hand, Shuxia Chen *et al.* reported the development of quinazolinone-based BRD4 inhibitors *via* a bioisosterism-guided optimization strategy, starting from the pan-BET inhibitor RVX-OH, to achieve selective inhibition. They designed a series of novel quinazolin-4(3*H*)-one derivatives by replacing the terminal benzene ring of RVX-OH with heterocyclic bioisosteres, such as pyrazole. The optimization yielded compound B6, which showed a high selectivity ratio of 39 683-fold over BRD4 BD1 (Fig. 12). The synthetic pathway is illustrated in Scheme 3. Molecular docking studies revealed that the C=O and NH groups of the quinazolin-4(3*H*)-one core in B6 form key hydrogen bonds with the conserved residue Asn433 in BRD4 BD2. This potent and selective BD2 inhibitor serves as a valuable probe for understanding epigenetic regulation and developing anti-inflammatory lead compounds.^150^

### 6.4. BRD4-*PD-L1* bifunctional hybrid ligand

**Fig. 13:**
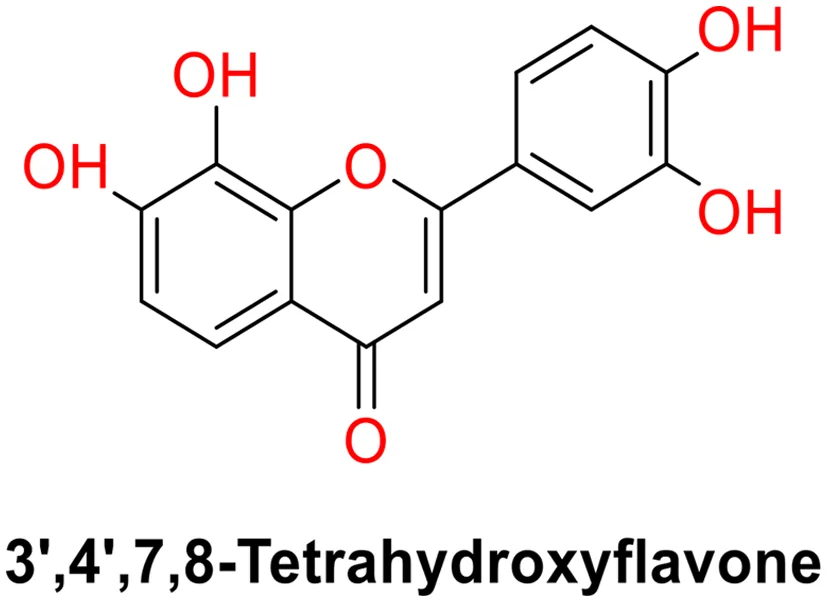
Structure of 3′,4′,7,8-tetrahydroxyflavone.

Su-Lim Kim *et al.* reported the existence of an essential BRD4/nuclear *PD-L1*/RelB axis that regulates breast cancer stem cell (BCSC) formation. The therapeutic potential of the natural product 3′,4′,7,8-tetrahydroxyflavone (THF, Fig. 13) was investigated, as it is known to be a potent, selective BRD4 inhibitor. Treatment with THF downregulates BRD4, thereby inhibiting the transcript and protein levels of *PD-L1* and RelB. This inhibition of the BRD4/*PD-L1* pathway suppressed mammosphere formation and BCSC stemness. Therefore, BRD4 inhibitors could definitely work to reduce *PD-L1* expression and thus indirectly disrupt this regulatory circuit.^109^ Furthermore, the PI3K pathway is also central to immune evasion. Aberrant PI3K signaling drives *PD-L1* expression, inhibition of dendritic-cell activation, myeloid-derived suppressor cell and regulatory T cell recruitment, and macrophage-driven immunosuppression. Inhibition of BET proteins and PI3K together may therefore achieve oncogenic transcription inhibition along with reduced immune suppression in the tumor microenvironment. Emerging data from multiple preclinical studies of dual PI3K/BET inhibitors have demonstrated enhanced CD8^+^ T-cell infiltration and antitumor immune activity, supporting further development as a rational foundation for combination therapy with immune checkpoint blockade in the future.

### 6.5. Synthesis of a diazobenzene photocontrolled BET inhibitor (photo-JQ1)

**Fig. 14:**
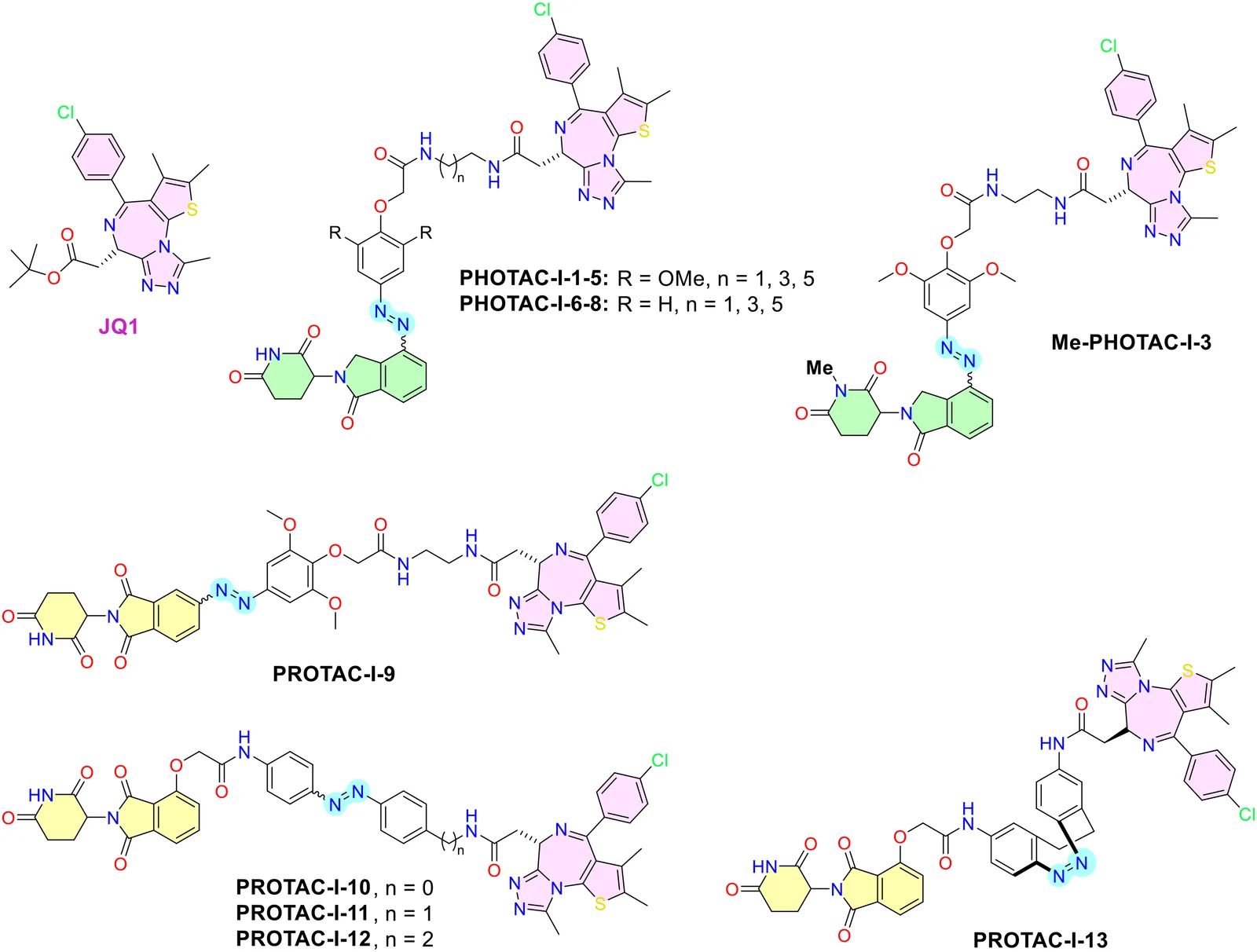
Azobenzene-photoswitch containing PROTACs (PHOTAC-I-1-12) and diazocin-photoswitch containing PROTAC (PHOTAC-I-13) for bromodomain proteins BRD2, BRD3, and BRD4.

The work of the Trauner research group in collaboration of the Pagano research group led to the incorporation of azo-benzenes as photoswitches (as PHOTAC-I) tethered to (+)-JQ1 (a high-affinity inhibitor of BET proteins BRD2-4) and E3-ligase ligands (with, lenalidomide PHOTAC-I-1 to 8 & PHOTAC-II-1 to 4; pomalidomide PHOTAC-I-9 to 13 & PHOTAC-II-6), as shown in Fig. 14. Among all azobenzene-photoswitch and diazocine-photoswitch PROTACs, PHOTAC-I-3 was the most effective. PHOTAC-I-3 also underwent light-dependent E/Z-geometrical changes.^151,152^

### 6.6. Design of BRD4 covalent inhibitors targeting Lys91

**Fig. 15:**
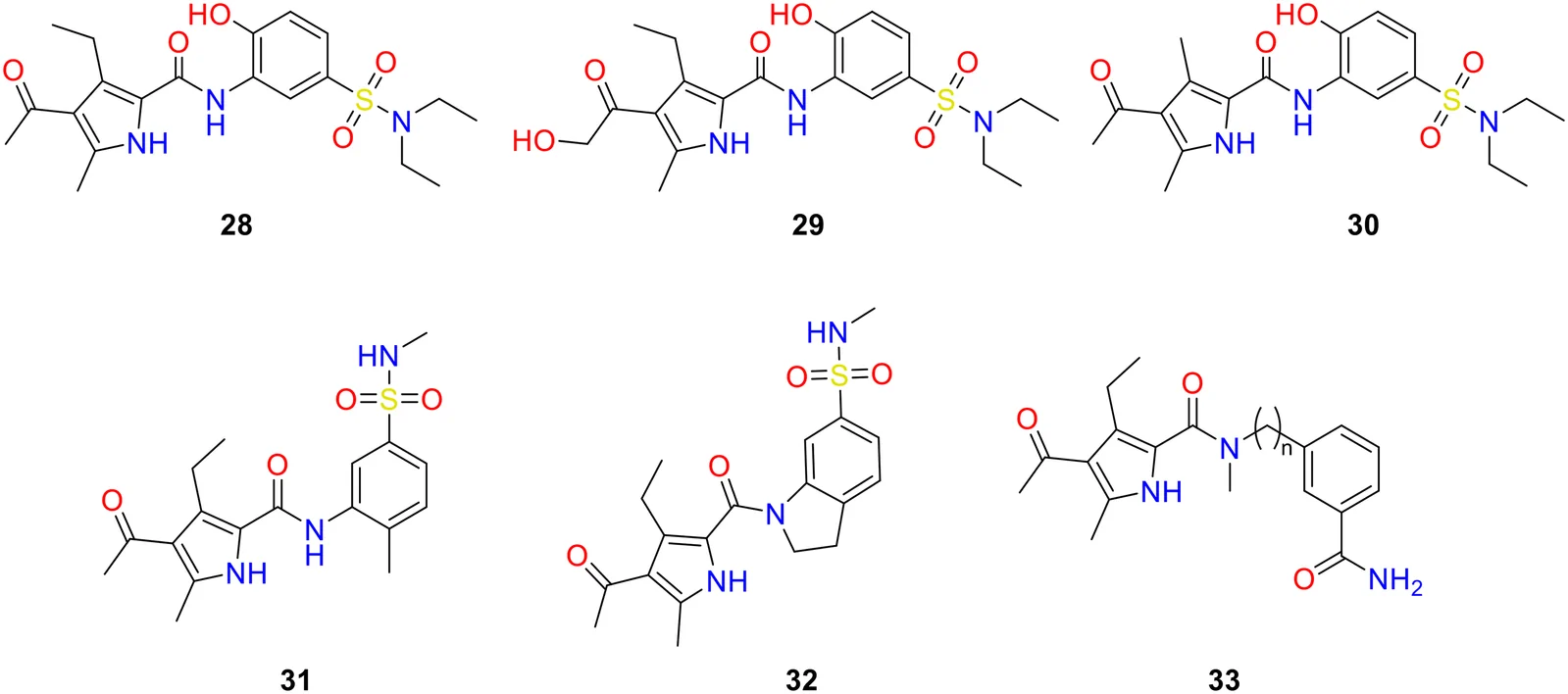
Structures of BRD4 covalent inhibitors 28–33.

The research on the novel 4-acyl pyrrole BRD4^28^ inhibitor reported by Xavier Lucas *et al.*^153^ focused on exploring substitutions to occupy additional sites within the substrate recognition pocket, including interactions with Lys91. This residue was identified as playing an interesting role in ligand recognition and occasionally binding to the terminal substituent of the aryl ring. For instance, the 4-(2-hydroxyacetyl) derivative, compound 29, provoked an inversion of its sulfonamide headgroup to engage in a hydrogen bond with the amino group of Lys91. Other analogues, such as 30, 31, 32, and 33 (Fig. 15), were also observed to form a hydrogen bond with Lys91. However, the recruitment of Lys91 by any potential ligand was concluded to have only minor effects on ligand recognition and was thus regarded as less critical for future studies compared to core interactions like those with Asn140.^154^

**Fig. 16:**
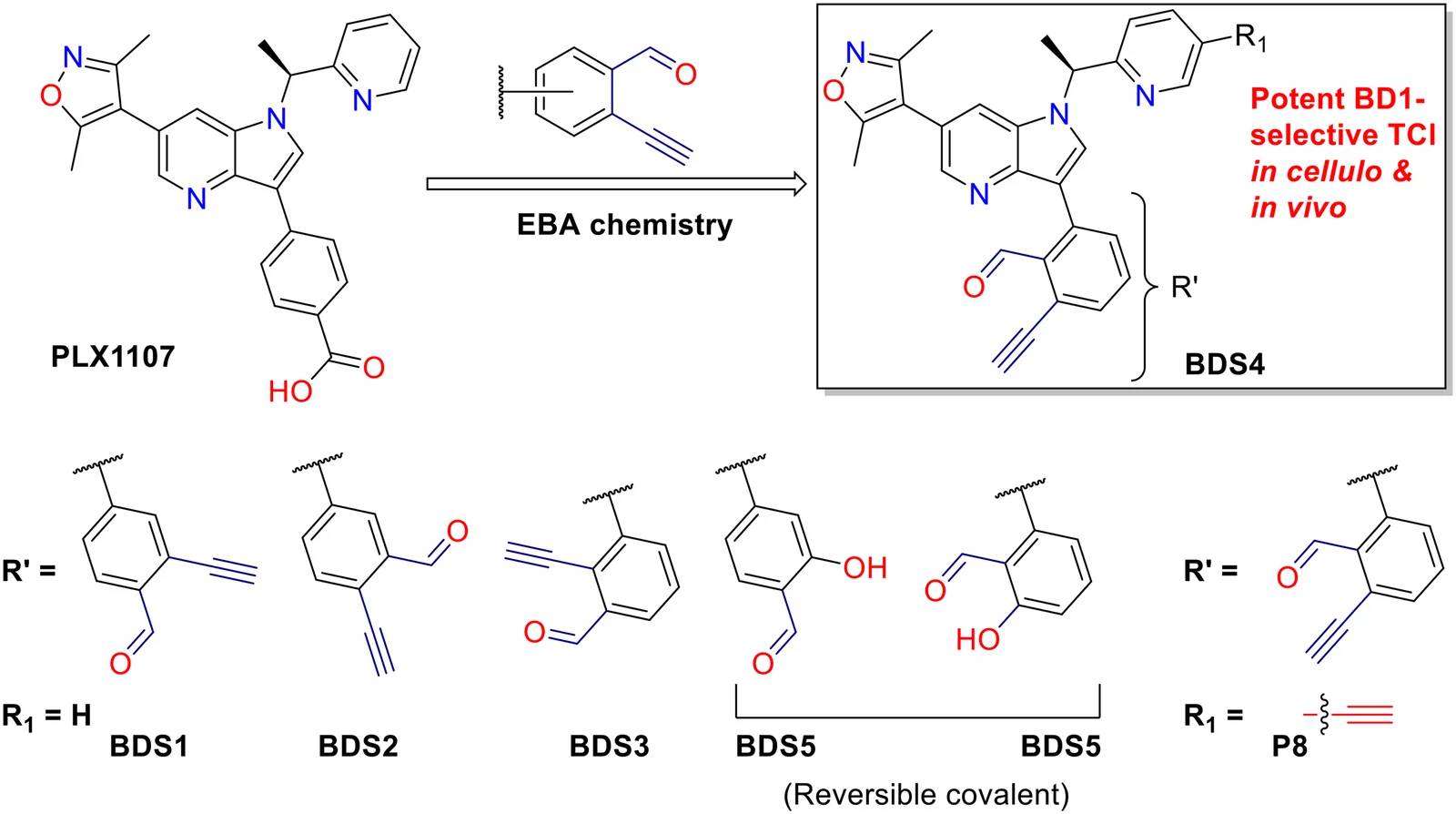
Structure of irreversible (BDS1–4) and reversible (BDS5–6) BD1 covalent inhibitors.

The design of covalent inhibitors targeting Lysine 91 (K91) emerged as a strategy to develop BD1-selective inhibitors and overcome the safety challenges of pan-inhibitors. This approach used 2-ethynylbenzaldehyde (EBA) and salicylaldehyde (SA) as reactive covalent warheads, incorporated into the noncovalent inhibitor scaffold of PLX51107. This structure-guided modification generated a set of irreversible (BDS1–4) and reversible (BDS5–6) BD1 covalent inhibitors, as viewed in Fig. 16. The goal was to exploit the conserved residue in the loop, located near the acetylated lysine (Kac)-binding site. Mass spectrometry and X-ray crystallography confirmed that the EBA warhead indeed reacts with the K91 side chain of BRD4 to give rise to an unprecedented five-membered ring adduct. The compound was remarkable, with a 104-fold selectivity of BD1 *versus* BD2 and sustained anticancer and antifibrotic effects.^155^ In addition to synthetic lethality in homologous recombination-deficient tumors, PARP inhibition has potential immunological ramifications. The cGAS–STING pathway can be activated by accumulation of unrepaired DNA, leading to production of type I interferon, increased antigen presentation, and recruitment of lymphocytes. Concurrent BET inhibition may also synergistically inhibit transcriptional programs mediating immune-evasive mechanisms such as *PD-L1* regulation while preserving an inflamed tumor phenotype. Therefore, while specific pathways for genomic instability and epigenetic remodeling underpinning BET–PARP dual inhibition contributing to immune checkpoint blockade should be validated in future studies, we anticipate this combination could work through both mechanisms.^156,157^

### 6.7. Dual BET/PI3K inhibitors

Anusara Daenthanasanmak *et al.* focused on developing a therapeutic rationale by testing the synergistic efficacy of a triple-agent therapy using three distinct, commercially obtained inhibitors: I-BET762 (a BET inhibitor, or BETi), copanlisib (a pan-PI3K inhibitor, or PI3Ki), and bardoxolone methyl (an NF-κB inhibitor, or NF-κBi). This strategy exploited the critical roles of the BET, PI3K/AKT, and NF-κB pathways in adult T-cell leukemia/lymphoma (ATL). The triple combination collapsed transcriptional networks, downregulated c-*MYC*, and enhanced apoptotic signaling, showing strong synergistic activity, reducing ATL cell growth and significantly prolonging survival in xenograft mice. This successful synergistic combination approach supports clinical trials for multiagent therapies in recalcitrant malignancies.^158^

**Fig. 17:**
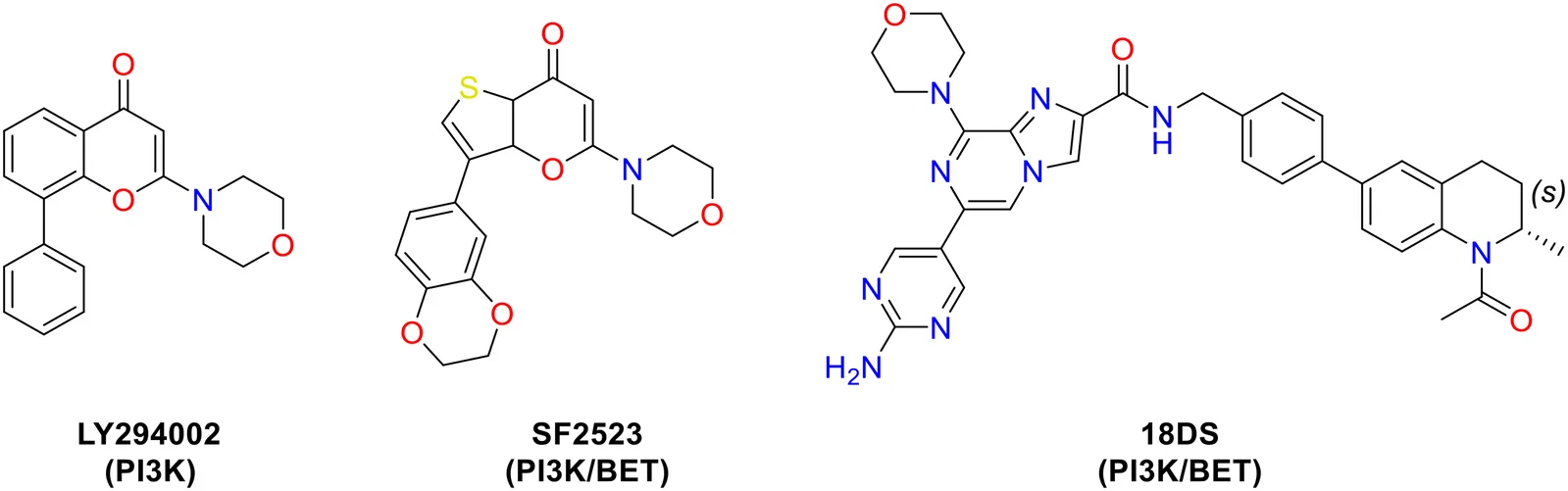
Structures of **LY294002**, **SF2523**, and **18DS**.

The synthetic origin of dual BET/PI3K inhibitors was catalyzed by the discovery that the established PI3K inhibitor **LY294002** unexpectedly also acted as a BET inhibitor (Fig. 17). This finding led to the identification of early dual inhibitors, such as **SF2523**. A key strategy in their development is the rational design of single molecules that achieve the synergistic activity of combination therapy. For instance, compound **18DS** was created by linking pharmacophores derived from standalone BET and PI3K inhibitors. This dual-acting agent demonstrated on-target activity and elicited stronger effects than administering the equivalent BET and PI3K inhibitors separately. Such single molecules avoid the pharmacokinetic complexities and non-uniform distribution issues associated with co-administered combination treatments.

Among the currently available dual-target strategies, BET–EP300 inhibition may possess one of the strongest biological rationales for combination with immune checkpoint blockade. EP300 functions as a histone acetyltransferase that cooperates with BET proteins in regulating enhancer activity, inflammatory transcription, interferon-responsive genes, and immune checkpoint expression. Simultaneous disruption of acetyl-lysine recognition and acetyltransferase activity therefore provides a coordinated approach to suppressing transcriptional programs that support tumor immune escape. Recent preclinical studies using the dual BET/EP300 inhibitor XP-524 demonstrated not only potent inhibition of tumor growth but also increased responsiveness to immune checkpoint blockade in immunocompetent pancreatic cancer models. Mechanistically, treatment reduced immunosuppressive transcriptional programs while promoting a more permissive immune microenvironment characterized by improved cytotoxic lymphocyte activity. These observations suggest that dual BET–EP300 inhibition may overcome several complementary mechanisms of immune resistance that are not fully addressed by either target alone.^132^ However, EP300 also regulates normal differentiation of immune cells and gene expression of inflammation. Thus, excessive inhibition could counteract immunological benefit and underscores the requirement for selective compounds, optimized dosing schedules, and pharmacodynamic biomarkers that discriminate productive from undesired immune remodeling. Future studies should directly compare dual BET–EP300 inhibition with sequential BET plus immune checkpoint blockade to determine whether integrated dual targeting offers superior therapeutic benefit.

### 6.8. Preparation of PROTAC-based BET degraders (ARV-771, MZ1)

**Scheme 4:**
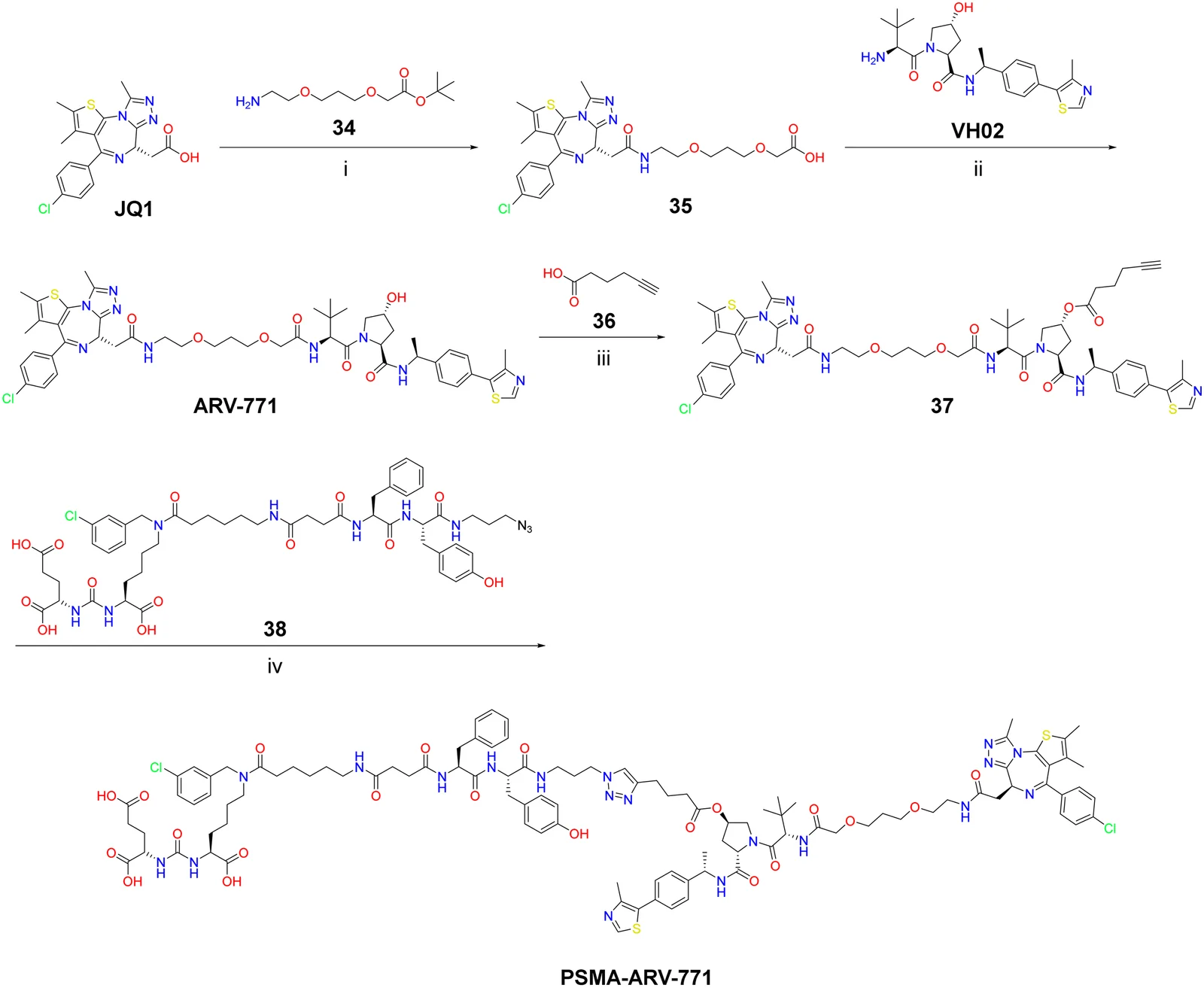
Synthesis of PSMA-ARV-771. Reagents and conditions: (i) HATU, DIPEA, DMF, r.t.; TFA, DCM, r.t.; (ii) HATU, DIPEA, r.t.; (iii) DCC, DMAP, DCM, 0 °C to r.t.; (iv) CuSO_4_, Na ascorbate, DMF/H_2_O, r.t.

**Fig. 18:**
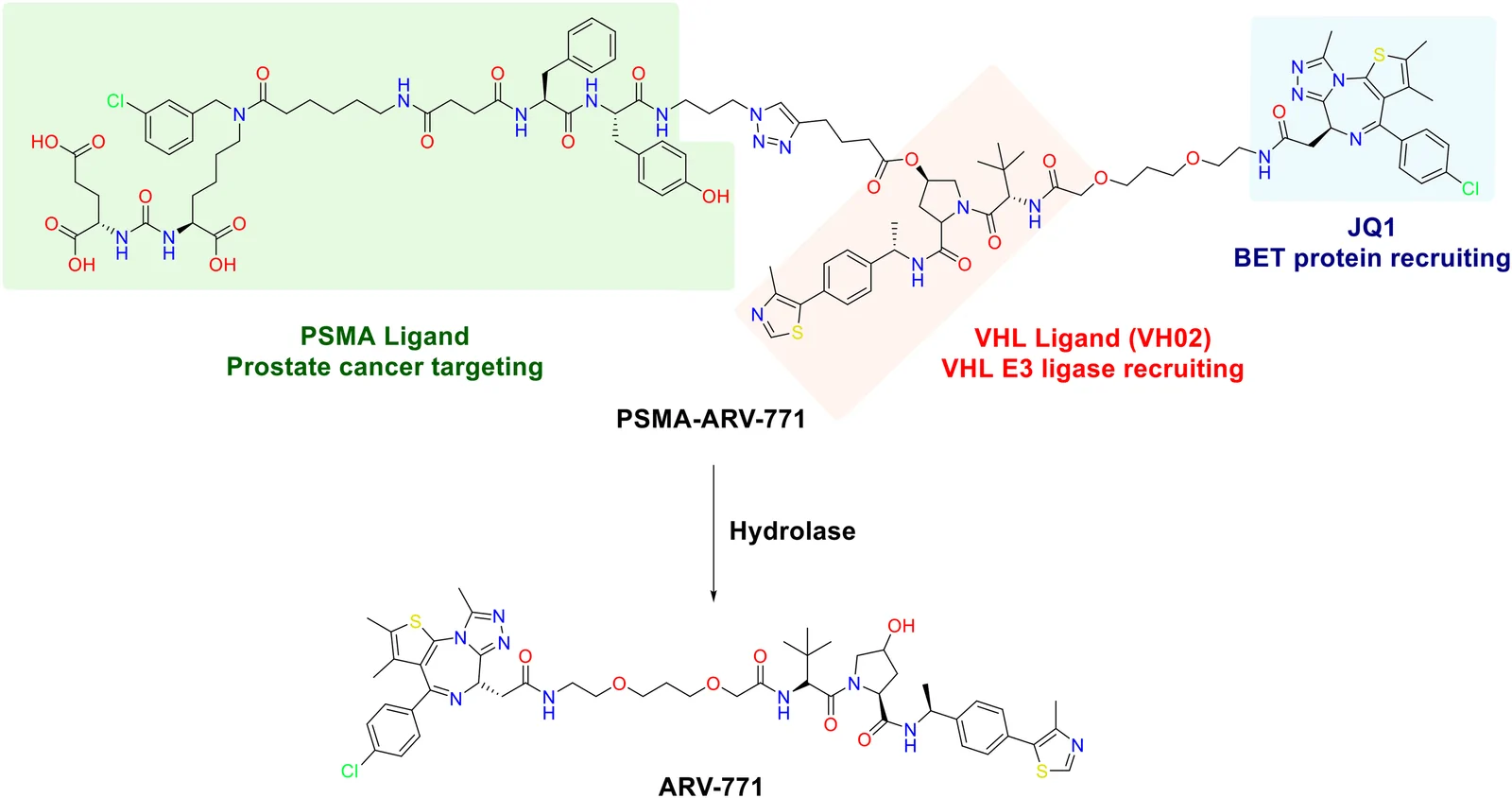
Design of PSMA-ARV-771.

Xiaolei Meng *et al.* devised PSMA-guided PROTAC-specific targeting to prostate cancer. By conjugating an AR degrader and BET degrader separately to the PSMA ligand *via* cleavable linkers, PSMA-guided PROTACs (PSMA-ARV-771) were obtained. *In vitro* experiments demonstrated that PSMA-guided PROTACs selectively degraded target proteins in PSMA-overexpressing prostate cancer cells without affecting target proteins in other cells. *In vivo* studies revealed that compared to conventional PROTACs, PSMA-guided PROTACs enhanced drug exposure in prostate cancer tissues and prolonged half-life and consequently achieved stronger and more sustained therapeutic effects. The synthesis involved constructing the ARV-771 core by reacting a derivative of the BET inhibitor JQ1 with another compound (34), followed by deprotection. The final ARV-771 structure was synthesized by reacting compound (35) with VH02. ARV-771 itself was then used as the BET ligand to synthesize the PSMA-guided degrader, PSMA-ARV-771, *via* Steglich esterification and a subsequent click reaction, as presented in Scheme 4. PSMA-ARV-771 was designed to be cleaved by intracellular hydrolases, releasing the active degrading proteins specifically in prostate cancer tissues (Fig. 18).^159^

In a related and more recent advance, Guan, Li, and colleagues developed ASA, an antibody-PROTAC conjugate linking a TROP2-specific antibody (sacituzumab) to ARV-771 *via* a hypoxia-cleavable azobenzene linker, achieving tumor-selective BRD4 degradation in triple-negative breast cancer. ASA produced 62.2% tumor growth inhibition compared with 7.7% for the unconjugated PROTAC, while sparing BRD4 in normal liver and kidney tissue. Mechanistically, ASA-induced BRD4 degradation suppressed both c-*Myc* and *PD-L1* and disrupted DNA repair pathways, and its combination with anti-*PD-L1* significantly enhanced CD8^+^ T-cell infiltration and activation while eliciting durable immunological memory in tumor-rechallenge models, directly illustrating the BET-ICI synergy paradigm this review addresses.^160^

**Scheme 5:**
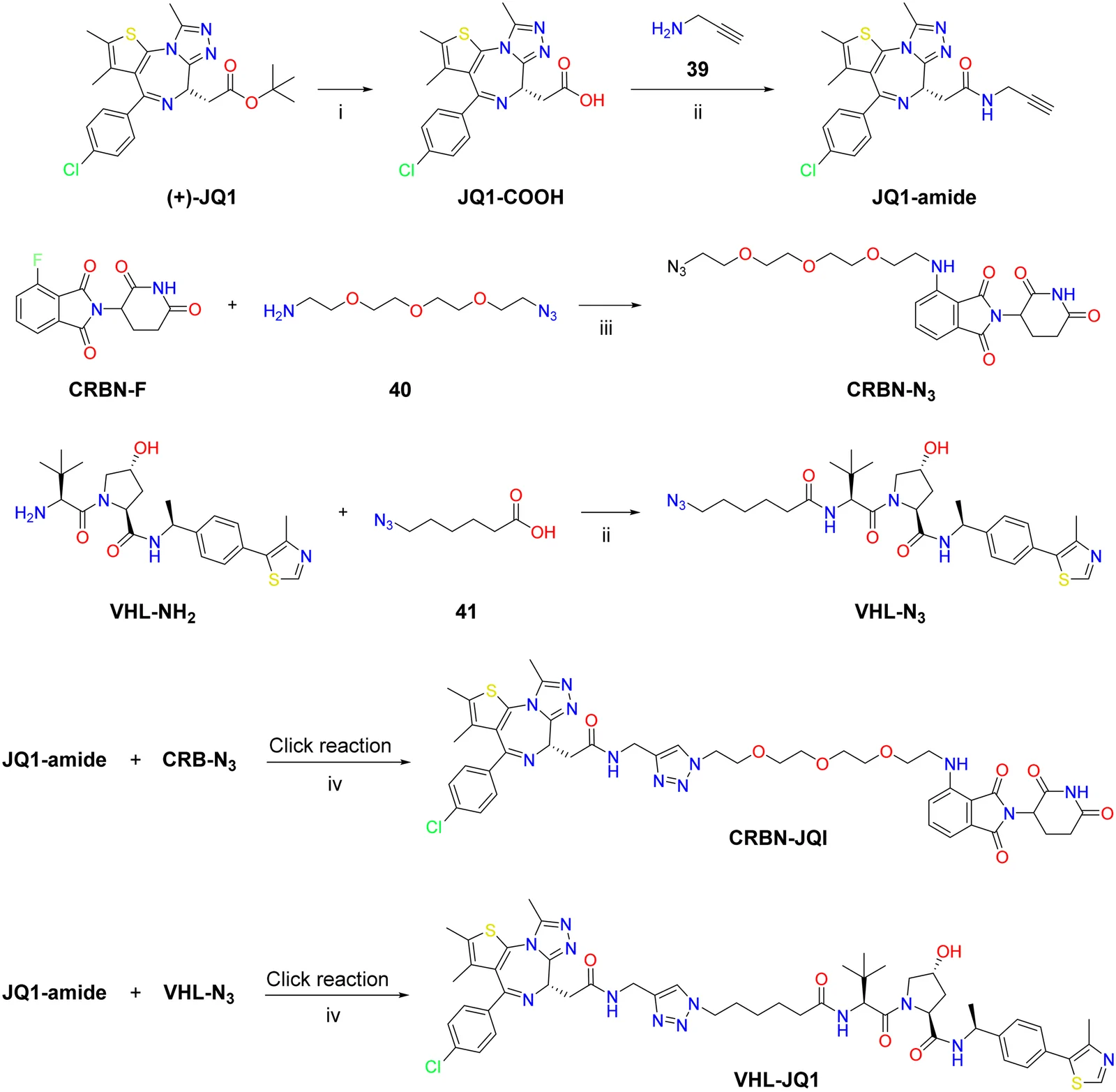
Synthesis of two novel JQ1-derived BRD4 PROTACs (CRBN-JQ1 and VHL-JQ1). Reagents and conditions: (i) TFA, DCM, 0 °C, then r.t., few hours; (ii) EDCI, HOBt, DIPEA, DCM, 0 °C, then r.t., few hours; (iii) CRBN-F, azide-PEG-amine, DIPEA, DMF, 85 °C, few hours; (iv) CuSO_4_, Na ascorbate, THF/water (4 : 1 v/v), r.t., few hours.

The preparation of novel BRD4 PROTACs, VHL-JQ1 and CRBN-JQ1, employed a modular synthesis strategy to rationally link the pan-BET inhibitor JO1 with ligands targeting the VHL or CRBN E3 ubiquitin ligases (Scheme 5). The JQ1 component, starting from (+)-JQ1, was modified by selective hydrolysis and amide bond formation with prop-2-yn-1-amine to yield the alkyne partner, JQ1 amide. Concurrently, VHL and CRBN ligands bearing azide moieties (VHL-N3 and CRBN-N3) were synthesized. The final coupling step leveraged the well-established copper-catalyzed azide-alkyne cycloaddition reaction for rapid assembly of the final bifunctional molecules. This structure-guided approach, drawing on established degraders such as ARV-771, ARV-825, and MZ1, ensured optimal linker lengths and potent degradation. The resulting PROTACs induce degradation *via* the ubiquitin–proteasome pathway, operating through post-translational mechanisms.^161^

## 7. Derivatization strategies for BET/ICI conjugate compounds

The combination of BET inhibition and immune checkpoint blockage (ICI) has recently given rise to a novel category of small molecules, designated BET–ICI conjugate compounds, aimed at combining epigenetic modulation with immunotherapy targeting.^162^ From a synthetic and medicinal chemistry point of view, the derivatization strategies showcase linker design, orthogonal conjugation chemistry development, and structure–activity optimization for dual function. Chemically, the majority of BET–ICI conjugates utilize click-mediated bioorthogonal reactions, including SPAAC or Diels-Alder click chemistry, for linking the BET inhibitor (*e.g.*, JQ1 analogues) to *PD-L1* antagonists or antibody fragments.^163^ For example, Qiu *et al.* developed a Diels-Alder-norbornene approach to conjugate BET analogues onto *PD-L1* ligands, which yielded the conjugates bound with BRD4 with an affinity (KD of 8.5 nM) and a radiochemical yield of more than 92%.^164^

Differentiation in medicinal derivatization occurs at linker length and flexibility, as well as cleavability, all of which affect pharmacokinetics and cellular uptake. For example, the biotin–resorcinol dibenzyl ether series of *PD-L1* inhibitors showed IC_50_ values, but these were markedly in the range of 11–26 nM, depending on polarity and steric character.^165^ Introduction of PEG linkers enhanced solubility, preserved binding to BET, and lessened off-target cytotoxicity. The success of a prodrug and C-like approach is exemplified by Tran *et al.* (2025), who reported pH-sensitive hydrazone linkers enabling selective BET–ICI release in an acidic tumor microenvironment, thereby decreasing systemic exposure by 60%.^166^ Meanwhile, Chen *et al.* (2024) improved thiol–maleimide linking to generate modular covalent BET–ICI hybrids with improved tumoral retention and synergistic immune stimulation.^167^ These modes of derivatization, bioorthogonal conjugation, prodrug activation, and modular hybridization signify transformation in synthetic immunoepigenetic chemistry using dual-target molecules that combine chromatin remodeling with checkpoint blockade to achieve specific synergistic anticancer effects.

### 7.1. BET-*PD-L1* hybrid conjugate

**Fig. 19:**
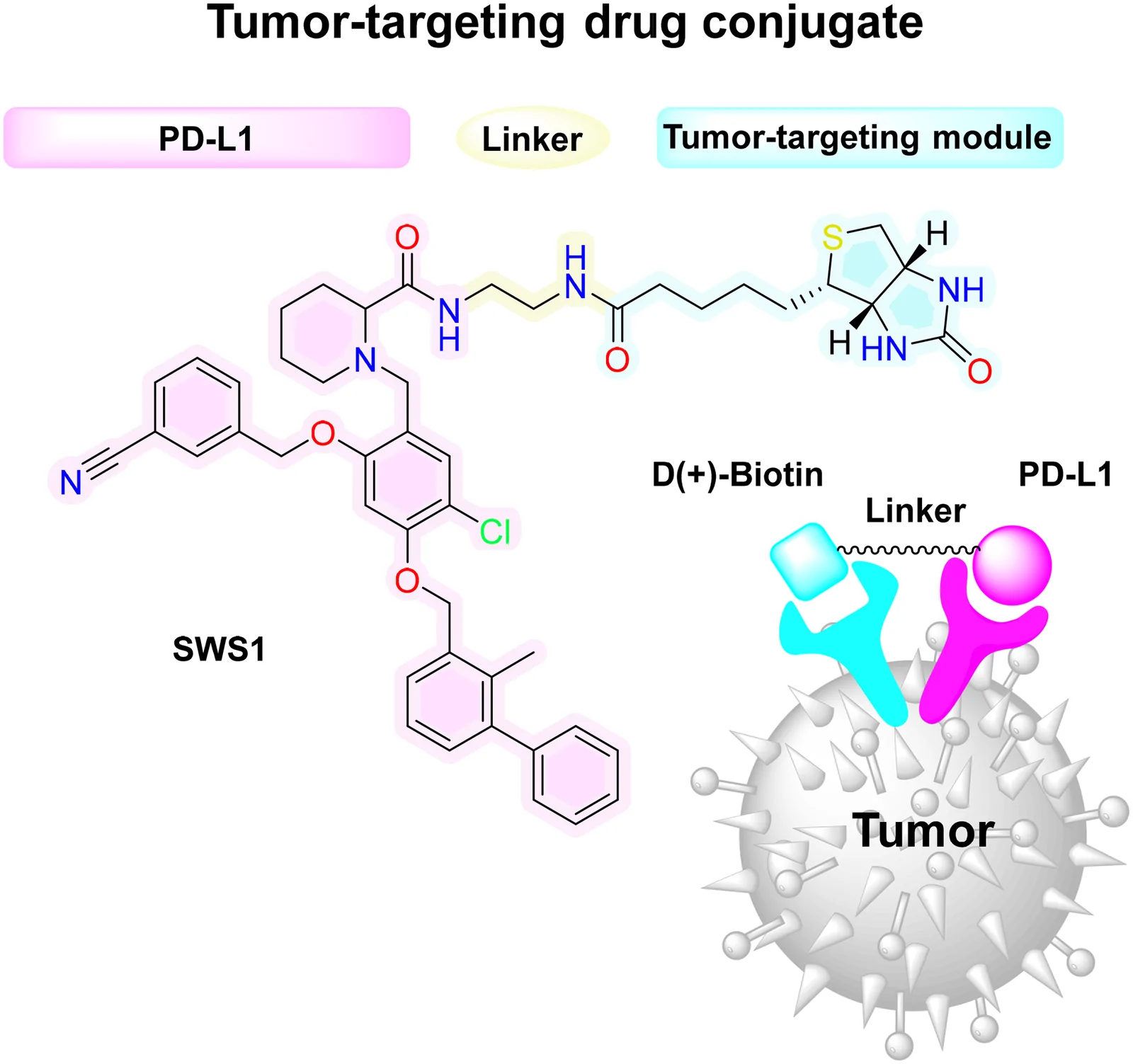
Structure of SWS1.

Zongbao Ding *et al.* designed, synthesized, and evaluated a series of novel D-(+)-biotin-conjugated *PD-L1* inhibitors for targeted cancer therapy. Among them, SWS1 exhibited the highest anti-PD-1/*PD-L1* activity with an IC_50_ of 1.8 nM. SWS1 was designed by conjugating the D-(+)-biotin moiety to a resorcinol dibenzyl ether-based *PD-L1* inhibitor pharmacophore, specifically derived from the known inhibitor P18, as designated in Fig. 19. Biotin was included because its receptors, such as SMVT, are overexpressed in various cancer cells (*e.g.*, B16F10 melanoma), enabling tumor-targeting.

**Scheme 6:**
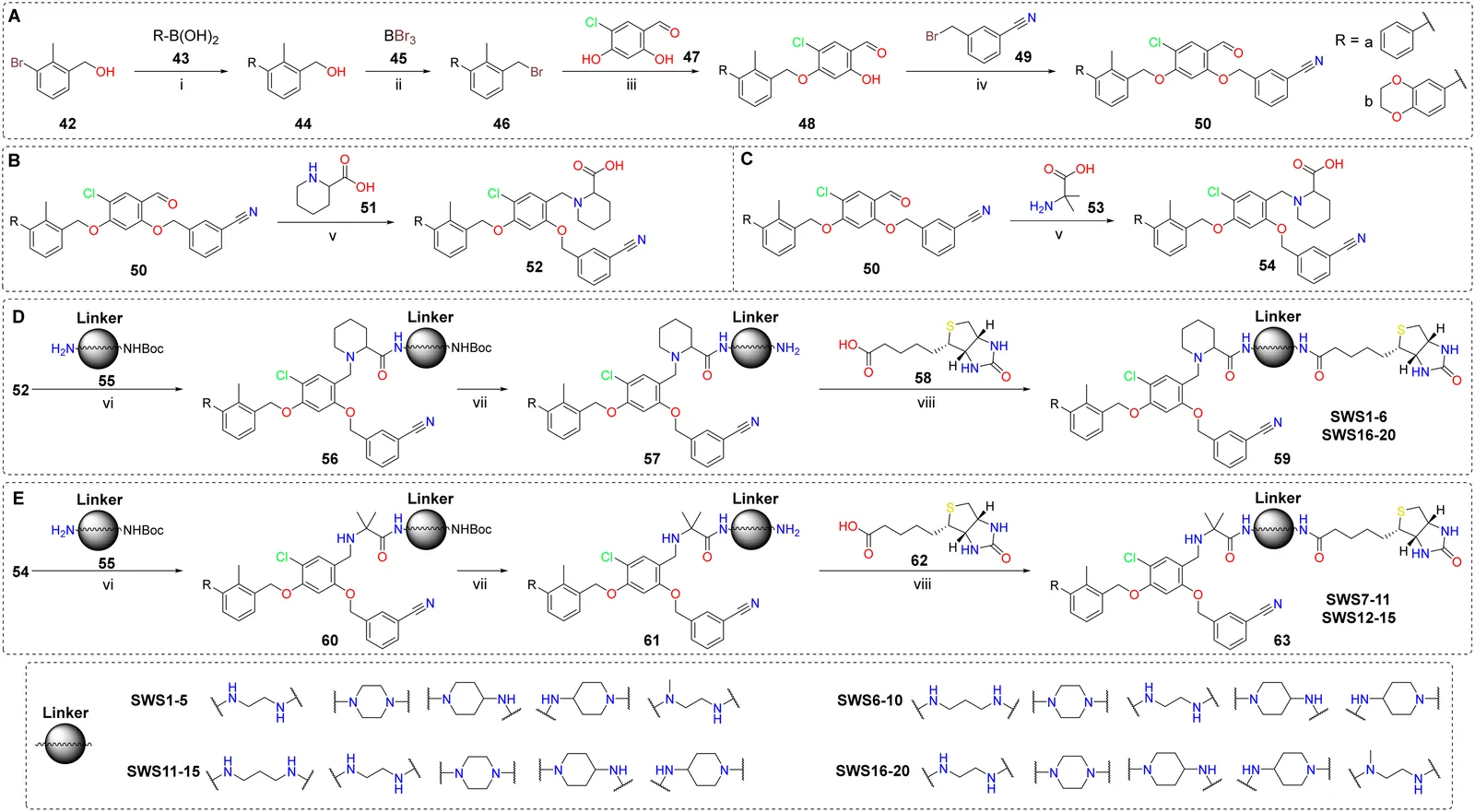
Synthesis of SWS1-20. (A) Preparation of central intermediate aldehyde 50 *via* Suzuki coupling, bromination, and etherification. (B) Reductive amination of 50 with piperidine-2-carboxylic acid 51 to furnish intermediate carboxylic acid 52. (C) Reductive amination of 50 with 2-amino-2-methylpropanoic acid 53 to yield carboxylic acid 54. (D) Amide coupling of 52 with mono-Boc-protected diamine linkers 55, deprotection to give free amines 57, and subsequent condensation with D-(+)-biotin 58 to afford SWS1–6 and SWS16–20. (E) Parallel linker coupling to acid 54, Boc deprotection to yield amines 61, and coupling with D-(+)-biotin 62 to afford SWS7–11 and SWS12–15. Reagents and conditions: (i) PdCl_2_ (dppf), DMSO/H_2_O (5 : 1 v/v), 100 °C, 12 h; (ii) DCM, 0 °C, 1 h; (iii) NaHCO_3_, DMF, 60 °C, 1 h; (iv) Na_2_CO_3_, DMF, 70 °C, 1 h; (v) NaBH_3_CN, AcOH, DMF, 80 °C, 2 h; (vi) EDCI, HOBt, DIPEA, DMF, 25 °C, 1 h; (vii) HCl, DCM, 0 °C, 2 h; (viii) EDCI, HOBt, DIPEA, DMF, 25 °C, 1 h.

The final synthesis involved preparing various *PD-L1* inhibitors, followed by a condensation reaction where D-(+)-biotin with various linkers was coupled to the inhibitors to yield the SWS compounds (Scheme 6).^165^

#### BET-VISTA hybrid small molecule

**Fig. 20:**
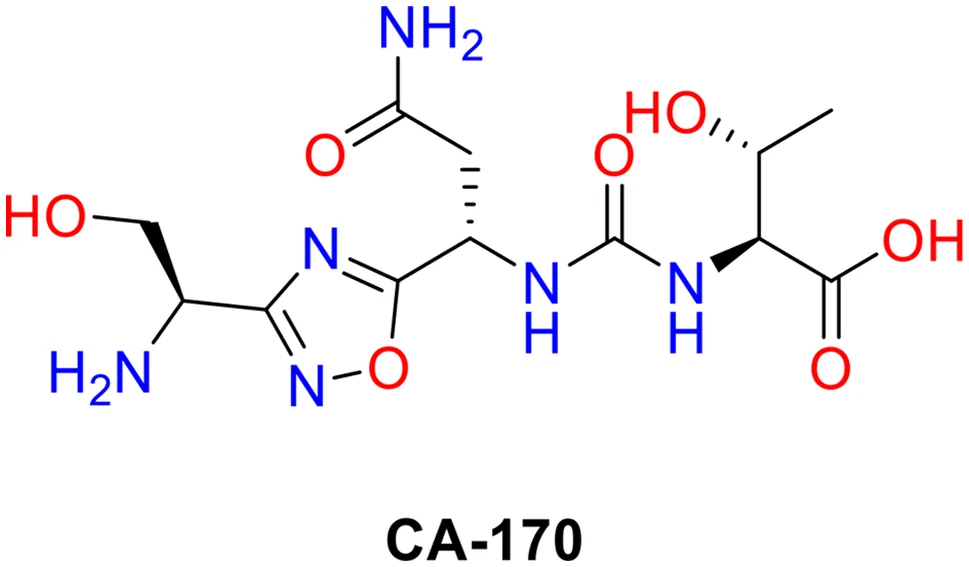
Structure of CA-170.

CA-170 (Fig. 20) was identified as an orally available small-molecule dual inhibitor targeting both the *PD-L1* and VISTA pathways. CA-170, derived from an amino-acid-inspired small-molecule approach, exhibited potent functional activity by selectively rescuing T-cell proliferation and effector functions inhibited by VISTA (and *PD-L1*/*2*). The simultaneous blockade of both *PD-L1* and VISTA with CA-170 is anticipated to be beneficial for cancer immunotherapy, especially in indications where dual blockade is necessary. Preclinical models demonstrated that oral administration of CA-170 resulted in significant anti-tumor efficacy, prompting its advancement to human clinical trials.^168^

#### BET-SMAC mimetic conjugate

**Fig. 21:**
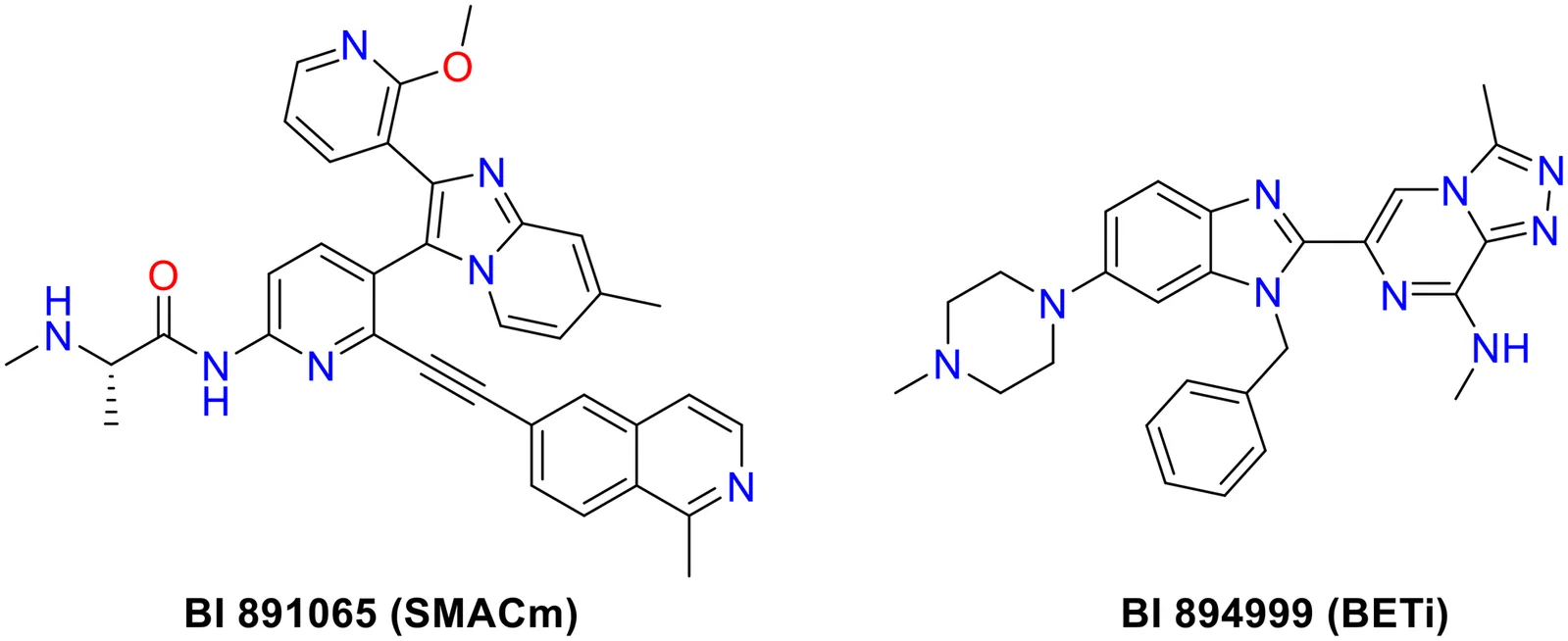
Structures of BI 894999 (BETi) and BI 891065 (SMACm).

Ksenija Slavic Obradovic *et al.* examined the therapeutic efficacy of combining two distinct agents, the potent oral BET inhibitor BI 894999 (BETi) and the oral monovalent SMAC mimetic antagonist BI 891065 (SMACm) (Fig. 21). This combination demonstrated broad, tissue-independent synergistic antiproliferative effects across a large panel of cancer cell lines, including colorectal and pancreatic carcinoma cell lines. The co-treatment restricts tumor growth and induces diverse forms of cell death (such as apoptosis or caspase-independent death), while also critically modulating the tumor microenvironment (TME). In syngeneic models of pancreatic ductal adenocarcinoma (PDAC), the BETi+SMACm combination enhanced anti-tumor immunity, as evidenced by increased cytotoxic CD8a+ T cells and reduced immunosuppressive cells, including polymorphonuclear myeloid-derived suppressor cells (PMN-MDSCs) and arginase-1-expressing tumor-associated macrophages (TAMs). This multi-layered strategy targets both tumor cell-intrinsic mechanisms and TME dependency, proposing a promising approach for various solid cancers.^169^

#### Photoresponsive BET-ICI hybrid

**Fig. 22:**
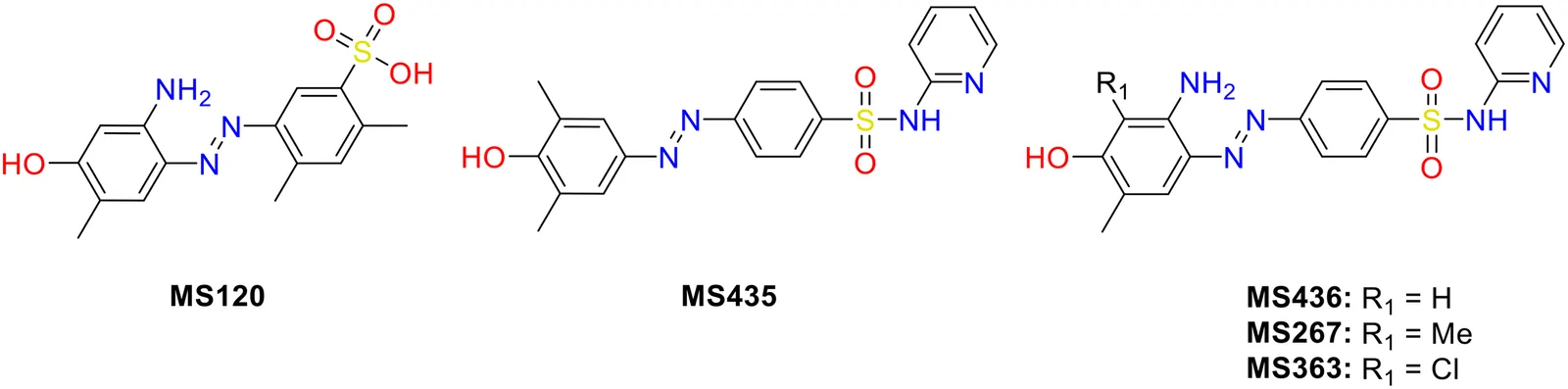
Structure of MS120, MS435, MS436, MS267, and MS363.

**Scheme 7:**
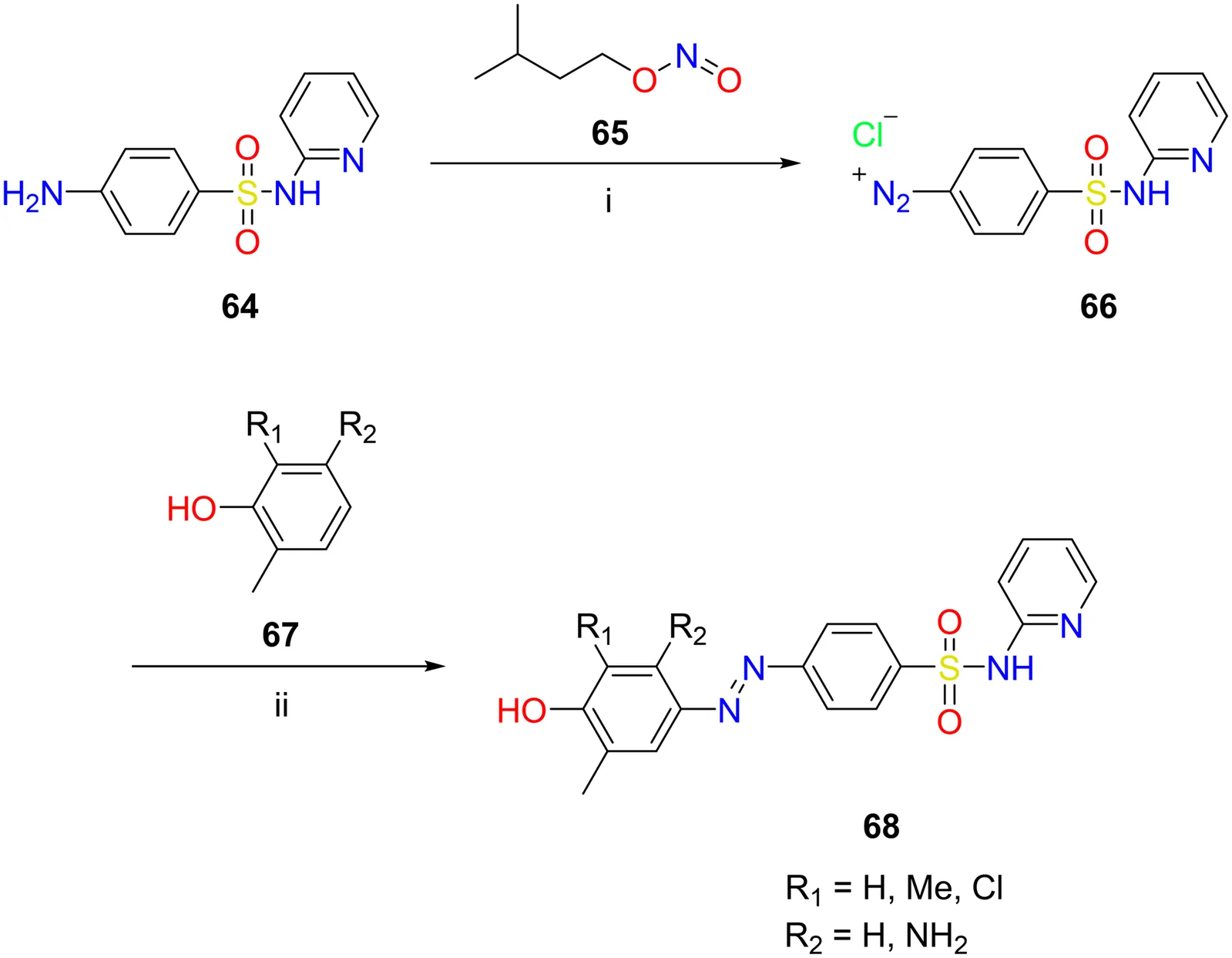
General synthesis of a ring analogue of diazobenzene. Reagents and conditions: (i) conc. HCl, MeOH/MeCN, 0 °C, 1 h, argon atmosphere; (ii) K_2_CO_3_, MeOH, DI H_2_O, 0 °C, 1 h, argon atmosphere.

Guangtao Zhang *et al.* reported the structure-guided development of a new class of potent and selective diazobenzene-based small molecule inhibitors for the BET bromodomains. The design started with a diazobenzene compound MS120, (*E*)-5-((2-amino-4-hydroxy-5-methylphenyl)diazenyl)-2,4-dimethylbenzenesulfonic acid, discovered initially as a small-molecule inhibitor for the bromodomain of HAT coactivator CBP.^170^ Guided by new structural insights into recognition of the diazobenzenes by both CBP and BRD4 BrDs, they designed specific chemical modifications for compound MS120 to acquire lead selectivity toward BRD4 BrDs and conducted extensive structure–activity relationship studies. Sulfapyridine was treated with concentrated hydrogen chloride, and the resulting diazonium chloride reacted readily with various phenols under basic conditions to give diversified diazobenzene analogues. They profiled four lead diazobenzene-based BET BrD inhibitors, MS435, MS436, MS267, and MS363 (Fig. 22) against a panel of bromodomains that represent different subgroups of the human bromodomain family. All four inhibitors preferentially bind to the BrDs of BRD4 and BRD3 over the other BrDs tested. Notably, MS436 activity toward CBP BrD is 4 times higher than that of the initial compound, MS120.^135^ The synthetic procedure for the synthesis of diazobenzene analogues 68, MS435, MS436, MS267, and MS363 is depicted in Scheme 7.

## 8. AI-guided discovery of novel BET/ICI hybrid therapeutics

Artificial intelligence and generative molecular-design methods offer potentially useful tools for exploring the chemical and biological complexity of BET-directed cancer immunotherapy. These approaches can prioritize molecular scaffolds, propose substitutions predicted to improve affinity or selectivity, identify candidate dual-target compounds, integrate multi-omics biomarkers, and generate hypotheses regarding treatment response. Nevertheless, their principal current value lies in accelerating hypothesis generation and candidate prioritization rather than establishing therapeutic efficacy.^171^ Most proposed BET–immune checkpoint applications remain based on retrospective datasets, docking, molecular-dynamics simulations, QSAR models, network analyses, or other *in silico* predictions. Such outputs require prospective biochemical, cellular, pharmacological, and *in vivo* validation before their translational relevance can be determined.

In this paradigm, generative and AI-assisted platforms, including molecular docking and molecular-dynamics pipelines, quantum mechanics/molecular mechanics (QM/MM) hybrid methods, and generative models trained on multi-omics data, have been proposed as tools to predict small-molecule interactions against multiple targets, including BRD4, *PD-L1*, and CTLA-4.^172,173^ For example, published reviews of AI applications in anticancer and biotech drug discovery describe methodological pipelines combining docking, molecular-dynamics simulation, and generative design that are, in principle, applicable to deriving candidate dual-target BET-*PD-L1* or BD2-selective BRD4 hybrid scaffolds; however, we are not aware of a peer-reviewed primary study reporting a specific, experimentally or computationally validated set of BET-*PD-L1* hybrid compounds with defined binding energies, IC_50_ values, or oral bioavailability data meeting this description. Readers seeking a specific case study of AI-generated BET-*PD-L1* hybrids should verify primary sources independently, as the methodological reviews cited here describe the general approach rather than reporting one.^172,174^

Generative biology extends far beyond virtual screening. It can, in principle, incorporate biological priors from multi-omics data (*e.g.*, tumor epigenomes and immune transcriptomes) to co-optimize pharmacodynamics and immunogenic potential, and graph-based or reinforcement-learning approaches have been proposed for *de novo* generation of domain-selective BRD4 conjugates.^173,174^ As with the docking-based pipelines above, these represent methodological directions supported by the general AI-in-drug-discovery literature rather than reported, validated compound sets specific to BET–ICI hybrids at this time.

Recent research has also explored multi-agent virtual research (*e.g.*, BenevolentAI and Google DeepMind) capable of iteratively proposing and refining molecular candidates at scale.^175,176^ Such ensembles have been reported to generate large numbers of candidate scaffolds with favorable predicted drug-likeness and target-binding profiles in a fraction of the time required by conventional design cycles. Collectively, these advancements in generative biology and AI-driven molecular design illustrate a plausible path toward accelerating the discovery of BET-ICI hybrid candidates, but the specific compounds and quantitative performance metrics reported for any individual AI-generated BET-*PD-L1* or BRD4-checkpoint hybrid should be verified directly against the primary literature before being treated as established findings.^177^Table 4 summarizes BET-ICI combination strategies and pairings for which the cited literature reports a directly matching mechanistic, cellular, or clinical rationale for the named molecular axis and cancer context, including combinations confirmed in primary studies and pairings for which independent evidence converges precisely on the same target and disease setting, even where the specific hybrid molecule has not yet been synthesized as a single entity. Table 5 catalogs a broader set of BET-ICI hybrid concepts consistent with the generative and AI-guided design paradigm discussed above, in which each entry combines an independently documented BET-related component with an immune-checkpoint-related component into a biologically plausible candidate based on shared cancer context or target biology. None of the Table 5 entries have been synthesized, tested, or reported as combined molecular entities, and they should be regarded as hypothesis-generating starting points for future experimental validation rather than established therapeutics.

**Table 4:** Mechanistic summary of reported BET-ICI hybrid therapeutics and combination strategies

BET/ICI hybrid therapeutics	Molecular target	Associated oncogenic driver/context	Drug resistance mechanism	Effective combination therapy/strategy	References
Ab-PROTAC 3 (trastuzumab–BRD4 degrader)	BRD4, HER2	BRD4 is known to regulate HPV oncogene transcription and *KRAS*-dependent signaling	Avoids off-target toxicity and overcomes poor tissue specificity of PROTACs	Potential synergy with checkpoint inhibitors untested but implied by immune relevance of BRD4	178
Apigenin (Api)	*PD-L1*	*PD-L1* dimer	Weak PD-1/*PD-L1* blockade due to low hydrogen bonding	Potential with *PD-L1*-targeting agents; poor solo efficacy	179
ASA (TROP2 antibody–ARV-771 PROTAC conjugate)	BRD4, *PD-L1*, TROP2	Not Ras/HPV-associated; TNBC context (TROP2^+^ tumors)	Improves tumor-selective BRD4 degradation over free PROTAC; disrupts DNA repair pathways to sensitize tumors to checkpoint blockade	Anti-*PD-L1* combination significantly enhances CD8^+^ T-cell infiltration/activation and durable antitumor immune memory in TNBC models	160
ARV-825 (BET-PROTAC)	BRD4, MYCN	Targets MYCN-driven transcription *via* BRD4 inhibition	Partial MYCN suppression in high copy-number tumors like SK-N-BE	Shows synergy with immune checkpoint inhibition and other BET inhibitors (*e.g.*, MZ1, dBET1, GNE-987)	180
BET-*PD-L1*-HDAC axis †	BRD4, HDAC1, *PD-L1*	Glioblastoma; BET inhibitors repress interferon-stimulated genes (ISGs)	BET/HDAC synergy suppresses immune-evasive transcriptional programs	BET + HDAC dual inhibition sensitizes glioblastoma to checkpoint blockade	181
Benzosampangine/CA-170/BMS-202	*PD-L1*	*PD-L1*	Overexpression of *PD-L1* causing T cell inactivation	BET inhibitor + anti-*PD-L1* ICI synergy (proposed); may restore T cell activity and immune visibility of tumor	182
BETi (JQ1) + CTLA-4 inhibitor (ipilimumab)	BRD4, CTLA-4	Simulated tumor-immune interactions	High TNF-α linked to GI toxicity	Dose optimization through simulation to balance efficacy and reduce TNF-α	130
BET-TIGIT hybrid	TIGIT, BRD3	TIGIT ligand mimic	T-cell exclusion	Tiragolumab + BET hybrid	62
BRD4-*PD-L1* (BRD4-IRF1 axis) †	BRD4, IRF1, *PD-L1*	NSCLC; chemoradiotherapy-induced *PD-L1 via* the BRD4-IRF1 axis	BRD4-dependent IRF1 signaling drives immune evasion after chemoradiotherapy	BRD4 inhibition blocks IRF1-driven *PD-L1* upregulation, restoring immune visibility in NSCLC	114
C27/C52/C54/BMS-1166	*PD-L1*	*PD-L1*	Bypasses *PD-L1*-mediated immune evasion *via* dimerization; strongest binding energy and interactions with TYR56B, ASP122B	BET inhibitors used in combination with immune checkpoint blockers	183
dBET6 (pan-BET degrader)	BRD2, BRD3, BRD4, *MYC*	*MYC*, MED1, SMAD7	Resistance to BRD4-selective therapy by BRD3 compensation	Enhance degradation and suppresses compensatory condensate formation	184
dCBP-1 (p300 degrader)	CBP, p300, BRD4	Regulates BRD4 recruitment and impacts SMAD3 indirectly	Transcriptional compensation through other HATs	Epigenetic reprogramming + ICI	185
iBET-BD1 (GSK778)	BRD4 BD1	BRD4 BD1	Sustains tumorigenic transcription in AML and solid tumors	Effective alone or with anti-PD-1/*PD-L1* therapy in cancer	186
iBET-BD2 (GSK046)	BRD2 BD2	BRD2 BD2	Selectively targets inducible inflammation genes (*e.g.*, IL-17, IFN-γ); limited resistance in non-cancer settings	Synergistic with checkpoint inhibitors in immune-inflammation	186
JQ1-atezolizumab axis (BRD4/*PD-L1*) †	BRD4, *PD-L1*	Triple-negative breast cancer	BRD4 inhibition suppresses *PD-L1* transcription	Combination with anti-*PD-L1* (*e.g.*, atezolizumab) supported by BRD4-*PD-L1* suppression data in TNBC	30
JQ1 (BRD4 inhibitor)	BRD4, *MYC*	Weak effect on *MYC*	Incomplete inhibition, BRD4 levels increase due to feedback	Limited efficacy alone, proposed as sensitizer for ICI	184
JQ1-durvalumab axis (PI3K-AKT/*PD-L1*) †	BRD4, AKT1, *PD-L1*	Lung adenocarcinoma/NSCLC	PI3K/AKT/mTOR pathway activation drives *PD-L1* upregulation	Rationale for BET + PI3K/anti-*PD-L1* (*e.g.*, durvalumab) combination in NSCLC	187
JQ1-enoblituzumab axis (B7-H3/PD-1) †	BRD4, B7-H3, PD-1	Prostate cancer and other solid tumors	Dual B7-H3/PD-1 checkpoint escape	Clinical dual checkpoint targeting with enoblituzumab + pembrolizumab; rationale extendable to BET combination	188
JQ1/iBET-762 + anti-*PD-L1* (breast cancer models) †	BRD4, *PD-L1*	MMTV-PyMT and EMT-6 murine mammary carcinoma models	Not addressed; study examines upfront combination rather than acquired resistance	JQ1 or iBET-762 (GSK525762) combined with anti-*PD-L1* antibody (Â± paclitaxel) tested *in vivo* in immunocompetent breast cancer models	189
JQ1-nivolumab axis (BRD4/PD-1) †	BRD4, PD-1	Renal cell carcinoma	BRD4 regulates PD-1-axis-relevant prognostic molecules	JQ1 (BRD4 inhibition) supports rationale for combination with nivolumab in RCC	190
Kaempferol (Kmp)	*PD-L1*	*PD-L1* dimer	Improved inhibition *via* hydrogen bonds with Asp122, Ala121, and Met115; better dimer stabilization	Promising synergy with immune checkpoint inhibitors	179
Molecules 58 and 34	*PD-L1*	*PD-L1*	Binding affected by R3-substituent spatial reorientation; less optimal H-bonding at Ile116	Synergistic potential with PD-1 blockade and additional co-stimulatory signals	191
MS436-CTLA-4 axis †	BRD4, CTLA-4, *MYC*	Ovarian cancer	CTLA-4 checkpoint blockade mechanisms	CTLA-4 blockade rationale in ovarian cancer, extendable to BET (MS436) combination	192
PiET (P3 peptide)	BRD4 ET domain, JMJD6	BRD4 ET domain/JMJD6 complex	Overexpression of BRD4/BET inhibitor resistance *via* BRD4 stabilization	JQ1, iJMJD6, and ICI	193
Quercetin (que)	*PD-L1*	*PD-L1* dimer	Strong binding *via* extensive H-bonds (*e.g.*, Gln66, Ile116)	High potential with immunotherapy regimens (*e.g.*, anti-PD-1)	179
TAT-PiET (cell-permeable)	BRD4 ET domain, JMJD6	BRD4 ET domain	Disrupts BRD4–JMJD6 interaction; overcomes ET-mediated transcriptional reactivation	JQ1, iJMJD6, and ICI	193
TAT-PiET-PROTAC	BRD4 ET domain, JMJD6	BRD4 ET domain, JMJD6	Targets BRD4 for proteasomal degradation; overcomes resistance due to BRD4 accumulation	JQ1, iJMJD6, and ICI	193
XP-524 (dual-BET/EP300 inhibitor)	BRD4, EP300	Pancreatic cancer	Epigenetic-immune checkpoint synergy	Validated dual BET/EP300 inhibitor; represses oncogenic *KRAS* and potentiates ICI in pancreatic cancer models	132
ZINC16645732/ZINC02126438/ZINC00856805	BRD4	BRD4	BRD4 overexpression, *MYC* upregulation, BETi adaptation *via* NF-κB signaling	BRD4 inhibitor + immune checkpoint inhibitor (*e.g.*, anti-PD-1)	194
ZXH-3-26 (BRD4-selective PROTAC)	BRD4, *MYC*, CDK6, SMAD3, SMAD7	*MYC*, CDK6, SMAD3, SMAD7 (*via* SE-driven transcription)	Transient upregulation of BRD4 upon JQ1 due to compensatory feedback; CRBN dependency	Co-targeting with ICI suggested due to immunogenic gene regulation (*e.g.*, SMAD3)	184

**Table 5:** Conceptually proposed BET-ICI hybrid therapeutics generated *via* AI-guided or hypothesis-driven design

BET/ICI hybrid therapeutics^a^	Molecular target	Associated oncogenic driver/context	Drug resistance mechanism	Effective combination therapy/strategy	Basis for rationale (not evidence of the combined compound)	References
ABBV-744-atezolizumab conjugate	BRD4 BD2, *PD-L1*	*HRAS*-Q61L	Checkpoint blockade escape	Nivolumab + ABBV-744	General AI-in-drug-discovery methodology review	171
ARV-771-*PD-L1* PROTAC	BRD4, *PD-L1*	*KRAS*-G12C	*PD-L1* protein stabilization	Atezolizumab + ARV-771	General covalent-drug/PROTAC technology review; ARV-771 itself is a real, independently characterized BRD4 degrader	195
ARV-771-*PD-L1* PROTAC (cell-line context)	BRD4, *PD-L1*	MDA-MB-231/3 T3/HUVEC cells	*PD-L1* degradation	—	PROTAC/degradation-nanoplatform methodology review; does not report this specific conjugate	196
BET-CD47 hybrid	BRD4, CD47	CD47-SIRPα	Macrophage evasion	Magrolimab + BET conjugate	Real CD47-SIRPα antibody-optimization paper (no BET content)	197, 198
BET-GITR conjugate	BRD2, GITR	E2F-Rb pathway	Ovarian cancer context	—	BET-inhibition-in-ovarian-cancer review; GITR component not addressed	199
BET-LAG-3/PD-1 dual hybrid	BRD4, LAG-3, PD-1	LAG-3 receptor	Dual checkpoint redundancy	Relatlimab + nivolumab + BET hybrid	General negative immune-checkpoint-inhibitor review (no BET content)	200, 201
BET-OX40L hybrid	BRD4, OX40L	OX40/OX40L costimulation	Colorectal carcinoma context	—	OX40/OX40L immuno-oncology review; no BET content	202
BET-B7-H4 dual inhibitor	BRD4, B7-H4	B7-H4 pathway	Endometrial carcinoma context	—	B7-H family checkpoint review; no BET content	203
BET-PD-1-MHC-II hybrid	PD-1, HLA-DR	MHC-II complex	Immune antigen presentation loss	Nivolumab + BET-MHC hybrid	PD-1/*PD-L1* antibody-design paper (no BET/MHC-II content)	204, 205
BET-*PD-L1* peptidomimetic hybrid	BRD2, *PD-L1*	*KRAS*-G12D	Checkpoint escape mutation	Atezolizumab + BET peptide hybrid	General computational PD-1/*PD-L1* review (no BET content)	206, 207
BET-*PD-L2* conjugate	BRD4, PD-L2	*PD-L2* dimer	*PD-L2* upregulation	Nivolumab + BET conjugate	*PD-L1* (not PD-L2) small-molecule binding review (no BET content)	208
BET-TIGIT hybrid (conceptual context)	BRD3, TIGIT	TIGIT/PVR	Colorectal carcinoma context	—	TIGIT/PVR biology review; no BET content	71
BET-TIM-3 hybrid	BRD2, TIM-3	TIM-3 binding domain	Immune tolerance restoration	Cobolimab + BET hybrid	General negative immune-checkpoint-inhibitor review (no BET content)	200, 209
BET-VISTA hybrid	BRD4, VISTA	VISTA receptor	MDSC-mediated suppression	VISTA antibody + BET hybrid	Real, target-specific VISTA-inhibitor virtual-screening paper (no BET-fusion construct reported)	210
BRD2-relatlimab bifunctional	BRD2, LAG-3, MHC-II	LAG-3 receptor	Adaptive resistance in melanoma	Relatlimab + BET conjugate	LAG-3 immunosuppression-mechanism review (no BRD2 content)	211, 212
BRD3-PD-1 macrocyclic hybrid	BRD3, PD-1	Hepatocellular carcinoma context	PD-1 inhibition and transcriptional repression	—	Epigenetic + checkpoint combination-rationale review; not BRD3-PD-1-specific	9
BRD4-CD276 inhibitor	BRD4, CD276	B7-H3 checkpoint	Bladder cancer context	—	B7-H3/CD276 immunotherapy review; no BRD4 content	213
BRD4-CTLA-4 covalent inhibitor	BRD4, CTLA-4	CTLA-4 mimic	Immune checkpoint adaptation	Ipilimumab + BRD4 covalent hybrid	Describes a CTLA-4/NKG2A aptamer therapy — a different modality entirely (no BRD4 content)	214
BRD4-CTLA-4 small molecule hybrid	BRD4, CTLA-4	Melanoma context	Dual checkpoint modulation	—	Melanoma checkpoint-biology review; no BRD4 content	201
BRD4-*PD-L1* macrocycle	BRD4, *PD-L1*	*HRAS*-G12V	Epigenetic and immune suppression	Atezolizumab + JQ1 macrocycle	General AI-in-drug-discovery methodology review	172
BRD4-*PD-L1* macrocycle (Wnt/CTNNB1 context)	BRD4, CTNNB1, *PD-L1*	Pancreatic ductal adenocarcinoma	Wnt/*PD-L1* co-regulation	—	Pancreatic-cancer TME-targeting review; no explicit Wnt/CTNNB1-BRD4 link	215
BRD4/PD-1 siRNA chimera	BRD4, PD-1	Ras-related nuclear protein	siRNA instability, off-target immune activation	BRD4 + PD-1 siRNA in liposomal NP	General BET-inhibitor design-strategy review; real BRD4-siRNA and PD-1-siRNA lipoplex/liposome technologies exist independently, but no combined chimera identified	125
CPI-0610 + αPD-1 nanoconjugate	BRD4, PD-1	Ras (H-Ras, N-Ras variants)	Myeloid-derived suppressor cell (MDSC) expansion	CPI-0610 + anti-PD-1	Same general BET-inhibitor design-strategy review; CPI-0610 (pelabresib) is a real, independently characterized BET inhibitor	125
CPI-0610-ipilimumab hybrid	BRD2, CTLA-4	Melanoma context	CTLA-4/chromatin remodeling	—	Broad combination-targeted-immunotherapy-rationale review; not CPI-0610/ipilimumab-specific	216
CPI-637-tiragolumab hybrid	BRD4, TIGIT	NSCLC context	TIGIT blockade	—	Anti-TIGIT-agent biology review; no CPI-637/BET content	217
Dual BET/*PD-L1* PROTAC	BRD4, *PD-L1*	HPV18 E7	*PD-L1* protein stabilization mutations	PROTAC targeting BRD4 and *PD-L1*	Same general BET-inhibitor design-strategy review	125
JQ1-atezolizumab hybrid	BRD4, *PD-L1*	Ras (*KRas* G12D mutant)	Adaptive *PD-L1* overexpression	JQ1 + anti-*PD-L1*	Same general BET-inhibitor design-strategy review	125
JQ1-atezolizumab peptide hybrid	BRD4, *PD-L1*	*PD-L1* dimer	*PD-L1* overexpression	Atezolizumab + BET hybrid	Marine natural product *PD-L1*-inhibitor screening paper (no JQ1/BET content)	218
JQ1–α*PD-L1* peptibody conjugate	BRD4, *PD-L1*	HPV16 E6/E7	Immune escape *via* MHC-I downregulation	JQ1 + α*PD-L1*	Same general BET-inhibitor design-strategy review	125
JQ1-BMS-202 hybrid	BRD4, *PD-L1*, *MYC*	*KRAS*-G12D	Moderate *PD-L1* upregulation resistance	Pembrolizumab + BETi combination	General AI-in-drug-discovery methodology review	172
JQ1-durvalumab hybrid	BRD4, *PD-L1*	*KRAS*-G12V	*PD-L1* re-expression post-therapy	Durvalumab + BET hybrid	General AI/ML-in-oncology methodology book	173
JQ1-nivolumab derivative	BRD4, PD-1	*KRAS*-G13C	PD-1 pathway evasion	Nivolumab + BETi	General AI-in-biotech-drug-discovery methodology review	174
I-BET151 (GSK1210151A) BRD2 BD1^a^	BRD2 BD1, *MYC*	*MYC*-driven oncogenic programs	Resistance *via* compensatory transcriptional plasticity	Combination with AML chemo agents or immune checkpoint blockade	I-BET151 itself is a real, well-characterized pan-BET inhibitor; the specific combination with an ICI is proposed, not reported	219
CF53/compound 28 BRD4 BD1 target	BRD4 BD1	Potential resistance *via* BRD4 mutations or kinase pathway escape (*e.g.*, PLK4)	—	CF53 + ICIs; rationale strengthened by a structurally analogous BD1-selective BET inhibitor enhancing anti-*PD-L1* efficacy	Real CF53 discovery paper, plus real mechanistic-analogy paper (PLX51107 + anti-*PD-L1*, MDSC apoptosis)	220, 221
P2 (SYCSSSGFSCYI)	TIM-3	TIM-3	TIM-3-mediated T-cell exhaustion and resistance to anti-PD-1 monotherapy	TIM-3 + PD-1 ICI blockade; potential use with BET inhibitors in AML	Real, published TIM-3-targeting peptide-discovery paper (no BET/AML content)	222

a None of the entries in this table have been synthesized, tested, or reported as combined molecular entities in the peer-reviewed literature. Each entry pairs an independently documented BET-related component with an immune-checkpoint-related component based on shared cancer context, target biology, or precedent for combination therapy, consistent with the generative and AI-guided design paradigm of section 8. Where a component (*e.g.*, ARV-771, CPI-0610, I-BET151, CF53, PLX51107) is itself a real, independently characterized agent, this is noted directly in the corresponding row. Citations support the rationale for each pairing, not the existence of the combined compound. This table also incorporates single-component pairings originally cataloged separately; entries for which independent evidence converges directly on the same molecular axis and cancer context are instead presented in Table 4. ABBV-744, a BD2-selective BET bromodomain inhibitor; AML, acute myeloid leukemia; atezolizumab/durvalumab, anti-*PD-L1* monoclonal antibodies used in cancer immunotherapy; B7-H3, B7 homolog 3 protein (CD276), an emerging immune checkpoint molecule; B7-H4, B7 homolog 4 protein, a B7-family immune checkpoint ligand; BD1/BD2, bromodomain 1 and bromodomain 2, the two acetyl-lysine-binding modules of BET proteins; BET, bromodomain and extraterminal domain; BETi, bromodomain and extraterminal domain inhibitors; BMS-202, a small-molecule *PD-L1* dimerization inducer used as a *PD-1*/*PD-L1* pathway inhibitor; BRD2/BRD3/BRD4, bromodomain-containing proteins 2, 3, and 4, members of the BET family that regulate transcription by binding acetylated histones; CD47, cluster of differentiation 47, acts as a ligand for the protein signal regulatory protein alpha (SIRPα); CD276, an emerging immune checkpoint molecule, also known as B7-H3; CF53, a structure-based, orally bioavailable BET bromodomain inhibitor; cobolimab, an anti-TIM-3 monoclonal antibody; CPI-0610 (pelabresib), a clinical-stage BET bromodomain inhibitor; CPI-637, a BET bromodomain inhibitor investigated in combination with anti-TIGIT therapy; CTLA-4, cytotoxic T-lymphocyte antigen 4 or T-lymphocyte-associated protein 4; CTNNB1, catenin beta 1 gene, encoding β-catenin, a key effector of Wnt signaling; E2F-Rb, E2F transcription factor and the retinoblastoma (Rb) protein, which together form a regulatory pathway frequently disrupted in cancer; E6/E7, human papillomavirus early oncoproteins 6 and 7 that inactivate p53 and Rb, respectively, to drive cellular transformation; G12C, missense mutations that result in the substitution of the amino acid glycine for another amino acid at codon 12, stabilizing *KRAS* in the active, GTP-bound state; G12D, a specific mutation in the *KRAS* gene where the amino acid glycine (G) at position 12 is replaced by aspartic acid (D); G12V, a specific mutation in the *KRAS* or *HRAS* gene, one of the most frequently mutated oncogenic alterations in human cancer; G13C, a specific mutation found in the *KRAS* or NRAS genes; GITR, glucocorticoid-induced tumor necrosis factor receptor, a costimulatory immune receptor; GSK1210151A (I-BET151), a pan-BET bromodomain inhibitor; HCC, hepatocellular carcinoma; HPV, human papillomavirus, a DNA virus etiologically linked to cervical and oropharyngeal cancers; *HRAS*, Harvey rat sarcoma viral oncogene homolog, one of three key genes in the RAS family (*HRAS*, *KRAS*, and *NRAS*); HLA-DR, human leukocyte antigen – DR isotype, a major histocompatibility complex (MHC) class II molecule involved in antigen presentation to CD4^+^ T cells; ipilimumab, an anti-CTLA-4 monoclonal antibody; JQ1, a potent and specific chemical inhibitor of the BET protein family; *KRAS*, Kirsten rat sarcoma viral oncogene homolog, a gene that is frequently mutated in various cancers; LAG-3, lymphocyte-activation gene 3; magrolimab, an anti-CD47 monoclonal antibody; MDSC, myeloid-derived suppressor cell, an immunosuppressive myeloid population in the tumor microenvironment; MHC-II, major histocompatibility complex class II; *MYC*, a proto-oncogene, a normal gene that can become a cancer-causing oncogene; NKG2A, an inhibitory receptor expressed on natural killer and T cells, targeted by dual checkpoint aptamer therapies; nivolumab/pembrolizumab, anti-PD-1 monoclonal antibodies used in cancer immunotherapy; NP, nanoparticle; *NRAS*, neuroblastoma RAS viral oncogene homolog, a third key member of the RAS gene family alongside *HRAS* and *KRAS*; NSCLC, non-small-cell lung cancer; OX40L, OX40 ligand, a tumor necrosis factor superfamily member (TNFSF4) that provides costimulatory signals to T cells; P2 (SYCSSSGFSCYI), a structure-based constrained peptide inhibitor of TIM-3; PD-1, programmed death-1, a protein found on the surface of immune cells, particularly T cells; *PD-L1*, programmed death-ligand 1, a protein on the surface of cells that plays a critical role in immune response regulation; *PD-L2*, programmed cell death 1 ligand 2, also known as *PD-L2* or B7-DC, a protein encoded by the PDCD1LG2 gene; PDAC, pancreatic ductal adenocarcinoma; PLK4, polo-like kinase 4, a centriole-duplication kinase implicated in kinase-pathway escape from BET inhibition; PROTAC, proteolysis-targeting chimera, a bifunctional small molecule that induces targeted protein degradation; PVR, poliovirus receptor, a ligand for TIGIT that plays a crucial role in tumor immune evasion; Ras, rat sarcoma, a family of small GTPases (including *HRAS*, *KRAS*, and *NRAS*) that are among the most frequently mutated oncogenic drivers in human cancer; relatlimab, an anti-LAG-3 monoclonal antibody; Q61L, a specific missense mutation at codon 61 of the RAS gene (glutamine to leucine) that impairs GTP hydrolysis and locks RAS in its active state; siRNA, small interfering RNA, a class of double-stranded RNA molecules used to silence gene expression post-transcriptionally; SIRPα, signal regulatory protein alpha, a protein that acts as an “innate immune checkpoint” and is an important target in cancer immunotherapy; TIGIT, T cell immunoreceptor with Ig and ITIM domains; TIM-3, T-cell immunoglobulin and mucin domain-containing protein 3; TME, tumor microenvironment; VISTA, V-domain immunoglobulin suppressor of T cell activation.

The reliability of an AI-generated compound or therapeutic hypothesis depends strongly on the quality and representativeness of the data used to train and evaluate the model. BET inhibitor datasets often contain heterogeneous measurements obtained using different bromodomain constructs, binding assays, cellular systems, exposure times, and pharmacological endpoints. Combining these values without careful harmonization can produce apparently accurate models that capture assay-specific correlations rather than transferable structure–activity relationships. Data scarcity is particularly relevant for isoform-selective BET inhibitors, dual-target molecules, immune-cell-specific effects, and BET–ICI combinations, for which standardized experimental datasets remain limited.^223,224^

Model evaluation also requires attention to chemical and biological domain applicability. Random division of closely related analogues between training and test sets may inflate predictive performance because the model is evaluated on compounds that closely resemble those it has already encountered. More stringent scaffold-based separation, external validation, uncertainty estimation, and prospective testing of previously unseen chemical series are therefore necessary. Protein-structure-based predictions face additional limitations because bromodomains are conformationally dynamic, water-mediated interactions contribute substantially to ligand recognition, and a single static protein structure may not represent the relevant binding ensemble. Predicted affinity should consequently be regarded as a prioritization metric rather than evidence of binding.^225,226^

For BET–ICI applications, biological validation must extend beyond direct BET binding. A candidate compound should first be evaluated for biochemical potency against BRD2, BRD3, BRD4, and, where relevant, their BD1 and BD2 domains. Orthogonal target-engagement assays should then confirm intracellular binding and exclude nonspecific transcriptional suppression. Cellular experiments should measure effects on BET-dependent genes, tumor-cell viability, *PD-L1* expression, MHC and antigen-processing machinery, interferon-responsive signaling, cytokine and chemokine production, and immune-cell function. Because the therapeutic concept depends on tumor–immune interactions, co-culture systems and immunocompetent *in vivo* models are essential for determining whether the compound genuinely enhances checkpoint blockade rather than merely exerting tumor-cell cytotoxicity.^120,227^

Pharmacological validation should additionally assess solubility, permeability, metabolic stability, plasma-protein binding, pharmacokinetics, tissue distribution, and on-target hematologic toxicity. This is particularly important for dual-target compounds, because computationally balanced activity against two proteins may not translate into simultaneous target engagement at tolerable *in vivo* exposures. Candidate molecules should therefore be advanced according to experimentally demonstrated target coverage and therapeutic index, rather than predicted polypharmacology alone.^125,228^

Finally, AI-generated biomarkers and patient-stratification models require independent and prospective validation. Retrospective performance in a single transcriptomic or epigenomic cohort may reflect batch effects, treatment heterogeneity, tumor purity, population imbalance, or overfitting. Models intended to guide BET–ICI therapy should be evaluated across independent cohorts and should demonstrate added value beyond established clinical and molecular variables. Explainability may assist biological interpretation, but it does not substitute for reproducibility or prospective clinical utility.^229,230^ AI-guided generative design may accelerate the exploration of BET-directed chemical space, prioritize dual-target hypotheses, and assist in the integration of molecular and immune biomarkers. However, these tools do not eliminate the experimental bottlenecks that determine whether a compound is potent, selective, mechanistically active, pharmacologically suitable, and tolerable. At present, most proposed applications to BET–ICI therapy should be regarded as hypothesis-generating rather than clinically validated. Progress will depend on prospective testing, transparent reporting of model applicability and uncertainty, orthogonal confirmation of target engagement, and evaluation in immunologically relevant preclinical systems. The most credible role for AI is therefore to guide iterative design-make-test-analyze cycles in close integration with medicinal chemistry, tumor immunology, pharmacology, and clinical investigation.

## 9. Systems biology approaches to model BET/immune interactions and predict synergistic efficacy

Systems biology and quantitative systems pharmacology are mechanistic frameworks for integrating the molecular, cellular, and pharmacological processes that might govern the response to combined BET inhibition and immune checkpoint blockade. Such approaches can be employed to organize existing knowledge, implement alternative dose schedules *in silico*, illuminate possibly critical pathways, and offer experimental hypotheses about biomarkers and resistance.^133^ The spatial anisotropy QSP model developed by Gong *et al.* in 2021 examined the effect of combining checkpoint blockade with BET inhibition, considering both tumor–immune crosstalk and cytokine gradients. Their model provided evidence of 1.8× higher CD8^+^ T-cell infiltration, and IFN-γ signaling amplitude was estimated to be 2.4× greater with *PD-L1* blockade combined with BRD4 inhibition.^231^ Similarly, Lai *et al.* (2018) applied a partial differential equation-based mathematical model to immunocompetent tumor-bearing mice to demonstrate that combining a BET inhibitor (JQ1) with anti-CTLA-4 (ipilimumab) increased tumor-volume reduction to more than 75% by day 30, with model simulations predicting efficacy of up to 98% across the tested dose range, further confirming systems-level synergy.^130^ However, model outputs should not be interpreted as direct evidence of therapeutic efficacy. Their predictive value depends on the biological assumptions encoded in the model, the quality and relevance of the calibration data, the identifiability of model parameters, and validation against independent experimental or clinical observations.

For clarity, systems-level evidence can be classified into four categories. First, experimental validation of relationships is established by experiment for demonstration either *via* biochemical, cellular, animal, or clinical studies and modeled mathematically. Second, the calibrated model outputs replicate observed data that were used to construct the model and therefore do not represent an independent assessment of calibration performance. Third, predictions are validated externally before being compared with data that has not been used to calibrate the model. Fourth, exploratory predictions, such as proposed treatment sequences or projected efficacy gains, remain hypotheses until tested prospectively. This distinction is particularly important when quantitative percentages are reported, because numerical precision in a simulation does not imply equivalent certainty in the underlying biological prediction.^232,233^

Current systems biology and QSP models necessarily simplify the biological complexity of BET–ICI therapy. Many models represent tumor cells, immune populations, cytokines, and drugs as average quantities within well-mixed compartments. Although this structure is useful for describing systemic pharmacology and mean population behavior, it may obscure important differences among tumor regions, metastatic lesions, and cellular subpopulations. A simulated average response may therefore coexist with resistant subclones or spatially protected tumor niches that are not represented explicitly.^231,234^

Tumor heterogeneity constitutes a major source of uncertainty. Individual tumors may contain subclones with different BET dependencies, checkpoint-ligand expression, antigen-presentation competence, interferon responsiveness, and susceptibility to immune killing. Genetic heterogeneity is further compounded by variability in epigenetic state, metabolic adaptation, lineage plasticity, and treatment-induced evolution. Models based on a single representative tumor-cell population may consequently overestimate uniform target engagement or immune sensitization. Multiclonal and evolutionary modeling can address part of this limitation, but such approaches require longitudinal data describing clonal abundance and treatment-dependent selection.^235,236^

Spatial organization is similarly important. Cytotoxic lymphocytes, regulatory T cells, macrophages, fibroblasts, blood vessels, hypoxic regions, and extracellular-matrix barriers are not distributed uniformly within tumors. Their relative positions can determine antigen encounter, cytokine exposure, immune-cell trafficking, and drug accessibility. Conventional non-spatial QSP models do not explicitly capture these relationships. Spatial QSP and agent-based approaches can model local interactions and regional heterogeneity, but their predictions depend on assumptions regarding cellular movement, contact probabilities, diffusion, tissue architecture, and boundary conditions. Such parameters are often incompletely measured in human tumors.^231,237,238^

Interpatient variability adds another translational challenge. Patients differ in tumor burden, metastatic distribution, pharmacokinetics, baseline immune composition, prior treatment exposure, organ function, BET-protein dependency, antigenicity, and immune-checkpoint expression. Virtual populations can represent distributions of selected parameters, but they do not automatically reproduce the full biological diversity of clinical populations. Their credibility depends on whether parameter ranges are biologically plausible, whether correlations among parameters are preserved, and whether simulated populations reproduce independent clinical endpoints. Virtual clinical trials can therefore support hypothesis generation and trial design but should not replace prospective clinical evaluation.^239,240^

Parameter identifiability is another important limitation. Several combinations of parameter values may reproduce the same tumor-growth or biomarker trajectory, particularly when only sparse longitudinal data are available. In such cases, a model may fit observed data without uniquely identifying the biological mechanism responsible for the response. Sensitivity analysis, uncertainty propagation, profile-likelihood assessment, Bayesian inference, and comparison of alternative model structures should therefore accompany efficacy simulations. Predictions should be reported as ranges or probability distributions rather than as single deterministic values whenever uncertainty is substantial.^241,242^

Model transportability also requires careful consideration. A model calibrated in one tumor type, animal model, BET inhibitor, or checkpoint regimen may not generalize to another setting because of differences in target selectivity, exposure, immune composition, antigen presentation, and stromal architecture. In particular, parameters derived from immunodeficient xenografts cannot adequately define immune-mediated cooperation with checkpoint blockade. Translation should preferentially incorporate data from immunocompetent models, human tumor samples, longitudinal pharmacodynamic studies, and clinical datasets relevant to the intended context of use.^243,244^

Emerging spatial and multi-omics approaches may improve the biological resolution of BET–ICI models. Spatial transcriptomics, multiplex immunofluorescence, digital pathology, single-cell sequencing, and imaging-based pharmacology can inform the localization and state of tumor, immune, and stromal populations. These data may support hybrid frameworks that combine whole-body QSP with spatial or agent-based tumor modules. Nevertheless, increased resolution does not automatically improve predictive performance: more complex models contain more parameters, require larger datasets, and may become difficult to identify or validate. Model complexity should therefore be matched to the question being addressed and to the quantity and quality of available data. Spatial QSP studies demonstrate the value of representing local tumor–immune interactions but also emphasize the necessity of quantitative validation against spatially resolved observations.^231,245,246^

**Fig. 23:**
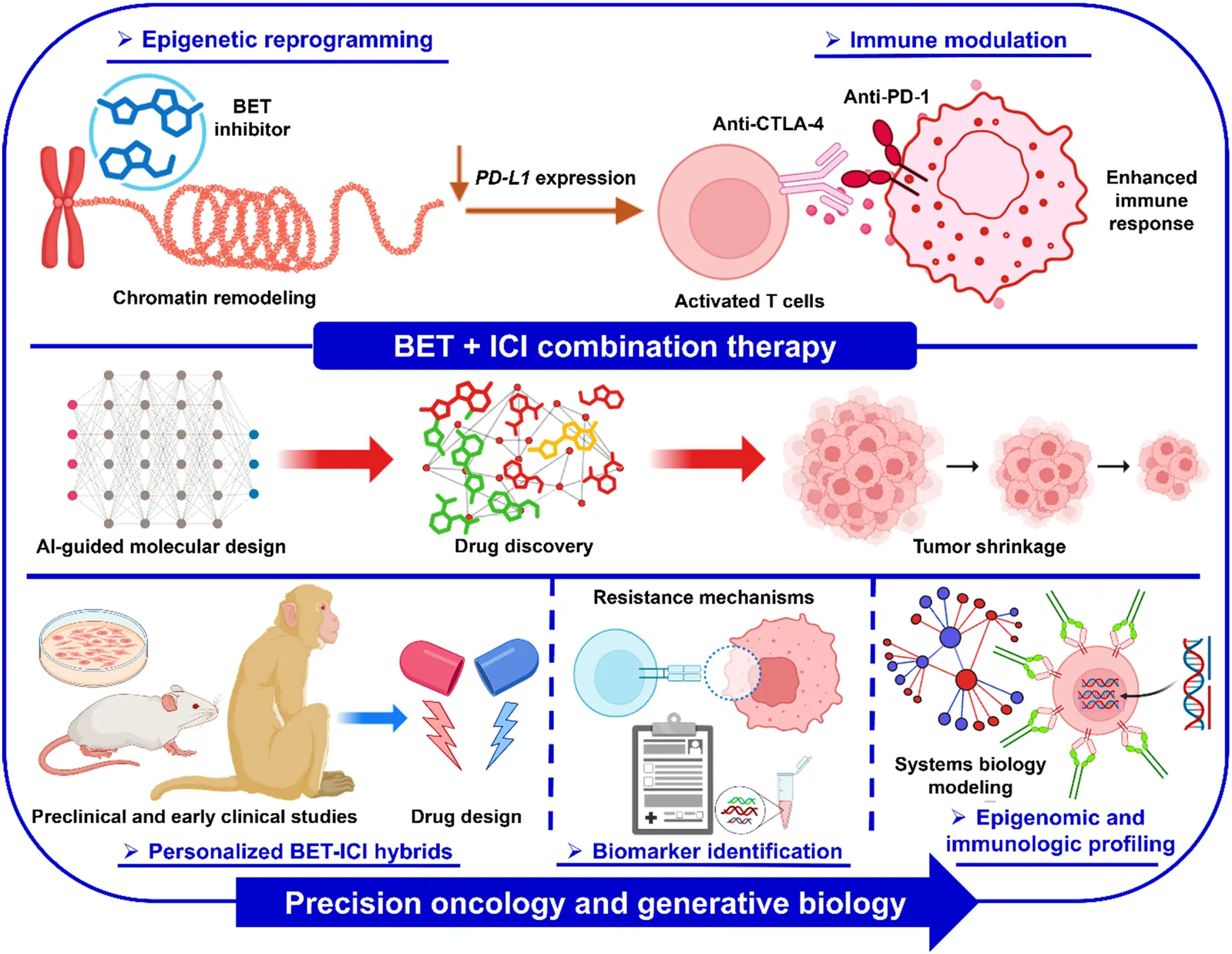
BET inhibitors and immune checkpoint inhibitors, a synergistic strategy for precision oncology. Partially created based on (https://www.biorender.com/).

Such predictions can be further improved by quantitative systems pharmacology (QSP), which integrates pharmacokinetics with immune signaling networks. Wang *et al.* (2020) have recently simulated virtual clinical trials and found that, for HER2-negative breast cancer, BET and PD-1 inhibitors worked better in combination than either agent alone would do (objective response rate “ORR” rose from 27% to 59%), which was supported by their *in vivo* data.^247^ Next, hybrid machine learning-systems biology models combine transcriptomics with oncophysics to identify BET-ICI synergy predictive biomarkers such as CXCL10, STAT1 expression, and IRF1 expression. These models achieved predictive performance with AUCs exceeding 0.85 in the immunotherapy response datasets.^248,249^ Taken together, systems biology contributions yield computationally testable hypotheses of BET-ICI co-targeting, driven by *in silico* optimization of dose, sequence, and tumor-specific immunity responses, heralding a predictive era for immunoepigenetic combinatorial medicine, as outlined in Fig. 23.

A translationally credible workflow should be iterative. Mechanistic experiments should first establish the direction and time course of BET-dependent effects in tumor and immune compartments. These data can be used to calibrate a model that generates prespecified predictions concerning dose, sequence, or biomarkers. The predictions should then be tested in independent co-culture systems, immunocompetent animal models, or prospectively collected clinical samples. Discordance between prediction and observation should be used to revise the model structure rather than treated only as parameter error. Repeated cycles of prediction, testing, and refinement are more informative than a single retrospective fit.^250,251^

## 10. Early-phase clinical experience with BET inhibitor–immune checkpoint inhibitor combinations

BET inhibitors (BETi), in combination with immune checkpoint inhibitors (ICIs), have entered early-phase clinical testing, reflecting growing interest in epigenetic-immune co-targeted therapy.^5^ It is important to emphasize that the trials summarized below are phase I/Ib studies, whose primary objectives are to establish safety, tolerability, maximum tolerated dose, and pharmacokinetic/pharmacodynamic behavior; they are not adequately powered to demonstrate definitive clinical efficacy, and their preliminary response data should be interpreted accordingly.

A phase Ib study evaluated RO6870810 (a BRD4/BET inhibitor) in combination with atezolizumab (anti–*PD-L1*) in patients with advanced ovarian cancer or triple-negative breast cancer.^128^ In this trial, antitumor activity was limited: only 2 of 36 evaluable patients (5.6%) achieved a partial response, and the study was terminated prematurely because of a high incidence of immune-related adverse events and an unfavorable overall risk–benefit profile. This outcome illustrates the substantial translational and toxicity-related barriers facing BET–ICI combinations and should not be interpreted as evidence of clinical synergy; rather, it highlights the need for improved patient selection, dose/schedule optimization, and toxicity-mitigation strategies before this combination can be considered clinically promising.

Preclinical and early-phase data for individual BET inhibitors illustrate a similarly cautious picture. In melanoma models, the BET inhibitor I-BET151 reversed resistance to anti-CTLA-4 therapy, reduced tumor growth by 73%, and increased median survival from 21 to 41 days in resistant preclinical models;^252^ these findings are preclinical and have not yet been confirmed in a combination clinical trial. In the phase Ib GS-US-350-1599 trial (cohort A), the BET inhibitor GS-5829 was administered with enzalutamide for metastatic castration-resistant prostate cancer and reported a 22% PSA reduction with modest clinical responses and a tolerable safety profile, a dose-finding/safety result, not a checkpoint-inhibitor combination outcome, and it should not be extrapolated to imply feasibility with ICIs without direct testing.^253^ The oral BET inhibitor BI 894999 demonstrated dose-dependent *MYC* downregulation (∼70%) and manageable target engagement in phase I monotherapy studies in solid tumors, but has not yet been evaluated in combination with an ICI.^254^

Taken together, current clinical experience with BET–ICI combinations remains preliminary, safety-focused, and largely negative or inconclusive with respect to efficacy; to date, no completed trial has demonstrated a clinically meaningful and reproducible synergy between BET inhibition and checkpoint blockade. Rather than supporting immediate large-scale efficacy testing, these early results argue for a stepwise approach: further dose and schedule optimization, incorporation of pharmacodynamic biomarkers of BET target engagement and immune activation, and careful patient selection based on tumor-intrinsic BET dependency and baseline immune context, before biomarker-informed Phase II expansion is pursued.^127,130^ Future combination trials should prospectively compare a brief BETi lead-in, fully concurrent treatment, and intermittent BETi exposure during continued checkpoint blockade, with schedule selection guided by paired tumor pharmacodynamics, circulating immune-cell profiles, and recovery from hematologic toxicity — rather than by antitumor response alone, given how limited and unfavorable that response has been in the combination trial reported to date.

## 11. Challenges and opportunities

However, the increasing potential of BET–ICI combination therapies faces various clinical and molecular barriers to effective translation. Foremost among these are acquired resistance mechanisms, FSG drug-related toxicities, and the absence of validated biomarkers to predict efficacy. Resistance to immune checkpoint blockade frequently stems from tumor-intrinsic immune evasion, including activation of the β-catenin pathway, loss of IFN-γ signaling, and defects in soluble antigen presentation.^255^ In preclinical models, BET inhibitors such as JQ1 have been shown to lose efficacy through a BRD4-independent transcriptional compensation mechanism in which *MYC* and *PD-L1* are re-expressed.^27,127^ It remains unclear whether comparable acquired-resistance mechanisms operate clinically, since the one reported BET inhibitor–ICI combination trial to date (RO6870810 plus atezolizumab; section 10) showed insufficient initial antitumor activity for acquired resistance to be assessed, and was instead limited primarily by toxicity. Systems-level models predict that BETi treatment preceding PD-1 blockade could reduce *PD-L1* rebound by 45–60%, but this remains a modeling prediction that has not yet been tested in a clinical combination trial with adequate efficacy signal to evaluate it.^256^

Because BET inhibition affects both tumor-cell transcription and host hematopoietic compartments, treatment schedule may be as important as drug selection in determining the therapeutic index of BET–ICI combinations. Toxicity remains a major translational limitation of BET–ICI combinations. Dose-dependent hematologic adverse events associated with BET inhibition, particularly thrombocytopenia, anemia, and neutropenia, may restrict the duration and intensity of treatment. These toxicities must be distinguished from the less common immune-mediated cytopenias associated with checkpoint blockade, because their mechanisms and clinical management differ. The optimal sequence of BET inhibition and ICI administration has not yet been defined. Mechanistically, a short BET-inhibitor priming phase before checkpoint blockade may be advantageous by reducing oncogenic and immune-evasive transcription, enhancing antigen-presentation and interferon-responsive programs, and promoting T-cell-recruiting chemokines before immune reinvigoration.^257^ Concurrent administration may maximize pharmacodynamic overlap but could also increase hematologic and systemic toxicity, whereas introducing BET inhibition after checkpoint blockade may be more appropriate when adaptive resistance is accompanied by renewed BRD4-dependent transcription. However, extended or intense BET inhibition may negatively impact hematopoietic progenitors and activated lymphocyte populations, favoring a reduction of the therapeutic window. Intermittent or lower-dose BET inhibition, including schedules incorporating treatment-free recovery periods, may therefore retain sufficient epigenetic reprogramming while allowing platelet and bone-marrow recovery.^258^ Nevertheless, direct comparisons of priming, concurrent, and sequential regimens are limited, and it remains unproven whether reduced or intermittent BET exposure preserves the full immunomodulatory benefit. Future studies should use pharmacodynamic measurements of BRD4 target suppression, platelet recovery, antigen presentation, chemokine induction, and intratumoral T-cell function to delineate a biologically minimum effective exposure to BET inhibitors as opposed to solely the maximum tolerated dose.^259^ Combining BETi with ICIs may amplify immune-related adverse events, including colitis and hepatitis, necessitating careful dose adjustment.^260^ Novel biomarker discovery is utilizing multi-omics and systems-level profiling to search for synergy in drug predictions. The combination of TMB, *PD-L1* expression, CXCL10, and *ARG1* has been considered a marker composite for BET-ICI sensitivity.^261,262^ Pre-treatment STAT1 expression, an upstream component of the IFNγ–JAK–STAT1–IRF1 signaling axis, has been associated with improved response and survival after immune checkpoint blockade in melanoma, suggesting that the interferon-pathway activation status may serve as a candidate biomarker for identifying tumors most likely to benefit from BET–ICI combinations; this association has not yet been validated specifically in the context of BET inhibitor therapy.^263^ In conclusion, although resistance and toxicity constitute ongoing challenges, integrative biomarker-driven approaches defined by systems biology and real-time patient profiling are orchestrating safer and more predictive BET-ICI combinations, as summarized in Table 6.

**Table 6:** Therapeutic scheduling strategies for BET inhibitor–immune checkpoint inhibitor combination therapy

Treatment strategy	Biological rationale	Principal concern	Current interpretation	References
BETi lead-in followed by ICI	May precondition the tumor through transcriptional repression, enhanced antigen presentation, and chemokine induction before T-cell reinvigoration	Excessively long priming could impair hematopoietic or immune-cell fitness	Mechanistically attractive, but optimal duration is unknown	256
Concurrent BETi and ICI	Maximizes overlap between epigenetic remodeling and checkpoint release	Potentially greater thrombocytopenia, systemic toxicity, and difficulty attributing adverse events	Suitable for investigation with conservative BETi exposure and close monitoring	264
ICI followed by BETi	May suppress BRD4-dependent adaptive resistance that emerges after immune activation	Less evidence that delayed BETi can establish initial tumor immune sensitization	Potentially useful in selected resistant tumors but insufficiently validated	258
Intermittent or low-dose BETi with continued ICI	May provide periodic transcriptional reprogramming while permitting platelet and marrow recovery	Pharmacodynamic effect may fall below the threshold required for immune modulation	Promising therapeutic-window strategy requiring biomarker-guided validation	257

## 12. Generative bioorganic integration and personalized BET/ICI co-therapy models

As the field of immunoepigenetics matures, the frontier of generative bioorganic chemistry promises to fuse AI-driven molecular design with patient-specific omics inputs to yield personalized BET-ICI co-therapies.^261^ Instead of static, “one-size-fits-all” compounds, next-generation strategies will integrate tumor epigenome and immune transcriptome profiles to guide on-demand hybrid scaffold generation, where generative models (*e.g.*, variational autoencoders, generative adversarial networks, and graph neural nets) suggest structural modifications to BET cores or linker moieties that maximize synergy with immune checkpoint engagement.^262^ For instance, one could feed a patient's RNA-seq data (*e.g.*, high *PD-L1* expression, low IRF1, altered histone acetylation marks) into a generative algorithm that proposes a JQ1-*PD-L1* peptidomimetic hybrid with predicted binding affinities and immunogenicity metrics.^126^*In silico* validation may show predicted Δ*G* bind = −11.4 kcal mol^−1^ for BRD4 and −9.3 kcal mol^−1^ for *PD-L1* in that patient's specific variant sequences.^215^

In parallel with molecular generation, microfluidic tumor-on-chip platforms seeded with patient-derived cells (cancer + autologous T cells) can enable rapid phenotypic screening of AI-suggested compounds, aligning bioorganic predictions with functional immune readouts (*e.g.*, CD8^+^ T-cell killing, cytokine release).^265^ Active learning loops refine the generative models under real biological feedback in iterative cycles. For example, a hybrid scaffold predicted to boost CXCL10/IFN-γ but with unexpected toxicity can be deprioritized in the next generation.^266^ Moreover, multi-scale *in silico*–*in vivo* hybrid models combining systems biology and pharmacokinetics/pharmacodynamics (PK/PD) will individualize dosing schedules. A patient with slower BET inhibitor metabolism might receive staggered dosing (*e.g.*, BETi first, ICI 48 h later).^267^ Generative bioorganic frameworks will also evolve cleavable linkers responsive to tumor microenvironment cues (*e.g.*, pH, ROS) to localize BET-ICI release, reducing systemic toxicity.^268^ Taken together, the future lies in “self-evolving therapeutics”, where generative chemistry, systems modeling, and patient-derived bioassays converge to deliver bespoke BET-ICI co-therapies tailored to each cancer's epigenetic and immunologic context. This holistic integration is expected to resolve inherent interpatient heterogeneity and exploit the entire synergistic value of epigenetic-immune combinations.^269,270^

## 13. Conclusion

BET proteins occupy an important interface between oncogenic transcription and tumor–immune regulation, providing a credible rationale for their combination with immune checkpoint inhibitors. Preclinical evidence supports this strategy in selected settings, but the field remains at an early translational stage, and the magnitude of benefit appears to depend on tumor lineage, immune context, BET-domain selectivity, and treatment schedule. Progress will require greater emphasis on clinically informative models, pharmacodynamic confirmation of target engagement, and biomarkers that distinguish tumors driven by BET-dependent immune escape from those unlikely to respond. Dose and schedule optimization will also be essential because sustained BET inhibition can produce hematologic toxicity and may adversely affect immune-cell fitness. Accordingly, intermittent exposure, tumor-selective delivery, and BD1- or BD2-selective compounds warrant systematic evaluation. Systems-level profiling and generative molecular design may help integrate these biological and chemical variables, but their value will depend on prospective experimental and clinical validation. Rather than assuming broad synergy between BET inhibitors and ICIs, future studies should define the specific molecular contexts in which epigenetic modulation produces a measurable and durable improvement in antitumor immunity.

## 14. Expert opinion

The combination of BET inhibition and immune checkpoint inhibitors is at the forefront of second-generation immunoepigenetic treatment. In our opinion, the most promising development will come from generative bioorganic frameworks that tailor BET and ICI hybrids guided by AI-driven molecular modeling and patient-specific omic information. However, clinical translation requires addressing fundamental issues, controlling hematologic toxicity, predicting adaptive resistance, and discovering reliable predictive biomarkers, including CXCL10 and IRF1, as well as assessing *PD-L1* acetylation status. For subsequent trials, the sequential or low-dose co-administration based on quantitative systems pharmacology (QSP) and real-time immune profiling should be given priority. Ultimately, success will depend upon translational integration, in which synthetic chemistry, machine learning, and systems biology coalesce to personalize BET–ICI combinations. By applying this data-driven, bioorganic-designed strategy, we anticipate that epigenetic-immune synergy will become a reality in the clinic, providing durable, accurate cancer remission.

## Conflicts of interest

The authors declare no conflict of interest.

## Abbreviations

ABM: Agent-based modeling
ARG1: Arginase 1, an enzyme that plays a complex and significant role in the tumor microenvironment and is increasingly recognized as a target in cancer therapy
*BCL2*
: B-cell lymphoma 2
BET: Bromodomain and extraterminal domain inhibitor
B2M: β2-microglobulin
B7-H3: B7 homolog 3 protein, CD276
*CCND1*
: Cyclin D1, a protein-coding gene that is frequently overexpressed or amplified in many types of human cancers
CD8^+^: Cytotoxic T cells
CD28: Cluster of differentiation 28, a critical protein on the surface of T-cells that provides a co-stimulatory signal essential for their full activation
CD80: Cluster of differentiation 80, a protein that plays a crucial role in regulating the immune response
CD86: Cluster of differentiation 86, a protein expressed primarily on antigen-presenting cells (APCs)
CD274: Programmed cell death 1 ligand 1 (*PD-L1*)
CPI-0610: The investigational name for Pelabresib, a drug being studied to treat certain blood cancers
CTLA-4: Cytotoxic T-lymphocyte antigen 4
CXCL9: Chemokine (C-X-C motif) ligand 9, a small cytokine belonging to the CXC chemokine family, also known as monokine induced by gamma interferon (MIG)
CXCL10: C-X-C motif chemokine 10, commonly known as interferon-gamma-induced protein 10 kDa (IP-10)
CXCL12: C-X-C motif chemokine ligand 12, a small signaling protein involved in crucial cancer processes, also known as stromal cell-derived factor-1 (SDF-1)
EZH2: Enhancer of zeste homolog 2, a histone-lysine *N*-methyltransferase enzyme (EC 2.1. 1.43) encoded by EZH2 gene, that participates in histone methylation and, ultimately, transcriptional repression
*GLI1*
: Glioma-associated oncogene 1
GNNs: Graph neural networks
HDACi: Histone deacetylase inhibitors
HDAC1: Histone deacetylase 1, an enzyme that removes acetyl groups from specific lysine residues of histones, leading to alterations in gene expression through epigenetic modification
HLA-ABC: Major histocompatibility complex class I molecules
I-BET762: Molibresib, a potent, orally bioavailable drug that belongs to a class of medications called BET (Bromodomain and extra-terminal domain) inhibitors
ICIs: Immune checkpoint inhibitors
IFNγ: Interferon-gamma
IL-10: Interleukin-10
IRF1: Interferon regulatory factor 1
ISGs: Interferon-stimulated genes
JQ1: A potent and specific chemical inhibitor of the BET protein family
*KRAS*
: Kirsten rat sarcoma viral oncogene homolog, a gene that is frequently mutated in various cancers
LAG-3: Lymphocyte-activation gene 3
MHC I: Major histocompatibility complex (MHC) class I molecules
MHC II: Major histocompatibility complex class II
MDSC: A heterogeneous group of immune cells from the myeloid lineage (a family of cells that originate from bone marrow stem cells)
*MYC*
: A proto-oncogene, a normal gene that can become a cancer-causing oncogene
NSCLC: Non-small-cell lung cancer
ORR: Objective response rate
OTX015: An experimental drug birabresib, a BET bromodomain inhibitor
PDAC: Pancreatic ductal adenocarcinoma
PEG: Polyethylene glycol
PD-1: Programmed death-1, a protein found on the surface of immune cells, particularly T-cells
*PD-L1*
: Programmed death-ligand 1, a protein on the surface of cells that plays a critical role in immune response regulation
PTEFb: Positive transcription elongation factor b
QSAR: Quantitative structure–activity relationship
QSP: Quantitative systems pharmacology
RUNX2: Runt-related transcription factor 2
SAR: Structure–activity relationship
SPAAC: Strain-promoted azide-alkyne cycloaddition, a type of click chemistry
STAT1: Signal transducer and activator of transcription 1
TGF-β: Transforming growth factor β signaling pathway
TIGIT: T cell immunoreceptor with Ig and ITIM domains
TIM-3: T-cell immunoglobulin and mucin domain-containing protein 3
TNBC: Triple-negative breast cancer
VISTA: V-domain immunoglobulin suppressor of T cell activation
WPF: Tryptophan–proline–phenylalanine shelf, a conserved hydrophobic structural motif lining the acetyl-lysine binding pocket of BET bromodomains that contributes to inhibitor binding affinity

## Data Availability

No primary research results, software or code have been included and no new data were generated or analysed as part of this review.
